# CD4^+^ T cells re-wire granuloma cellularity and regulatory networks to promote immunomodulation following *Mtb* reinfection

**DOI:** 10.1016/j.immuni.2025.01.001

**Published:** 2025-02-11

**Authors:** Joshua D. Bromley, Sharie Keanne C. Ganchua, Sarah K. Nyquist, Pauline Maiello, Michael Chao, H. Jacob Borish, Mark Rodgers, Jaime Tomko, Kara Kracinovsky, Douaa Mugahid, Son Nguyen, Qianchang Dennis Wang, Jacob M. Rosenberg, Edwin C. Klein, Hannah P. Gideon, Roisin Floyd-O’Sullivan, Bonnie Berger, Charles A. Scanga, Philana Ling Lin, Sarah M. Fortune, Alex K. Shalek, JoAnne L. Flynn

## Main text

(Immunity *57*, 2380–2398.e1–e6; October 8, 2024)

Following publication, we, the authors, identified three inadvertent errors. First, we realized that the data from the naive macaque control animals, specifically *Mtb* CFU and barcoding and PET-CT data, were previously published (see reference 9, Ganchua et al., in the references list). These macaques were originally part of the present study but were used as controls for another similar study that was submitted at the same time. These select data, from the control animals, were reused due to ethical and financial constraints of macaque research, and it was an oversight on our part to not mention that in the revised version of the *Immunity* article. This error does not affect the conclusions of the manuscript.

Second, the UMAP embedding depicted in Figure 3A featured mislabeled cDCs 1 and cDCs 2. This error was generated during figure compilation and does affect any analyses or conclusions.

Third, the original version of Figure 5A described “UMAP embeddings depicting monocyte-derived cell subpopulation densities, split by NHP cohort.” This density figure was removed from the final version of publication. The legend for this figure has been updated. The legends for Figures S3 and S4 were incorrectly labeled during the reformatting processes. The original version of Figure S3 only listed (D), but it should have read as (D and E). The original version of Figure S4 read as (C-D), but it should have read as (D and E). Additionally, in the Figure S4 legend, (E) should have read as (F), and (F) should have read as (G).

These unintentional errors do not affect any analyses or conclusions. The method details, Figure 3, and supplemental figure legends have now been corrected online. The authors apologize for these errors and any inconvenience they may have caused.

**Figure 3A:**
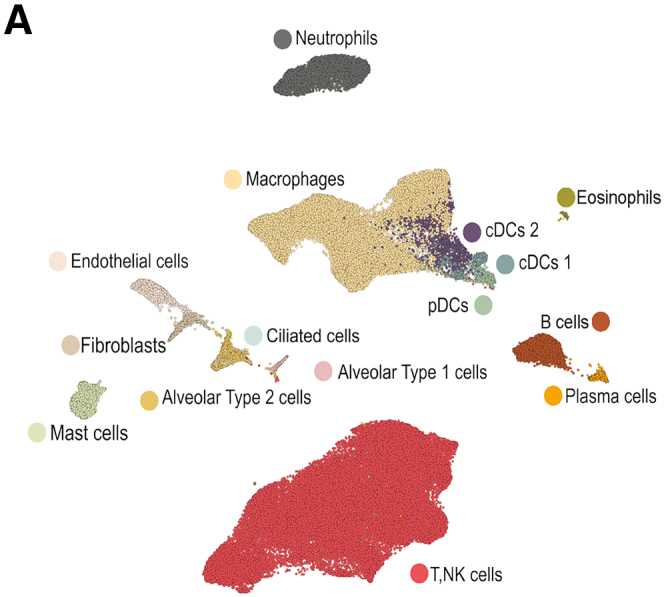
*Mtb* reinfection promoted cellular remodeling of the TB granuloma microenvironment (corrected)

**Figure 3A:**
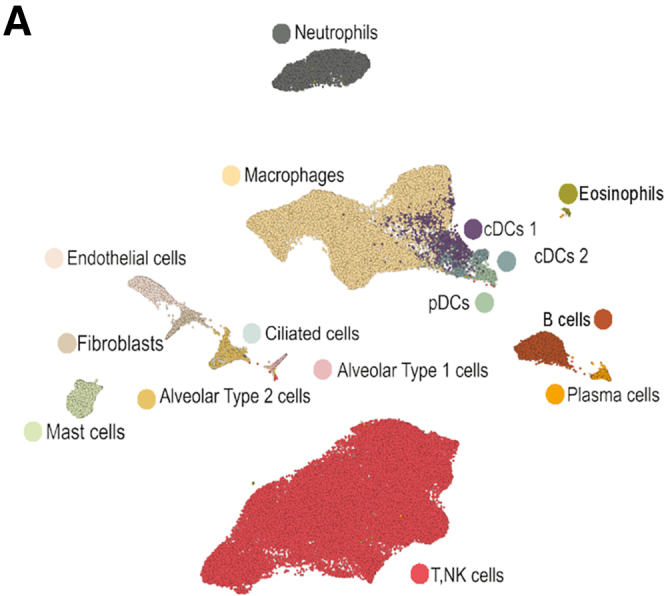
*Mtb* reinfection promoted cellular remodeling of the TB granuloma microenvironment (original)

