# Supplementary material for: CD4+ T cells re-wire granuloma cellularity and regulatory networks to promote immunomodulation following Mtb reinfection
Source: Immunity. 2024 Oct 8;57(10):2380–2398.e6. doi: 10.1016/j.immuni.2024.08.002 (PMC11466276; doi:10.1016/j.immuni.2024.08.002)
Supplement: Document S1. Figures S1–S5 [file mmc1.pdf]

Immunity, Volume *57*

Supplemental information

CD4^{+} T cells re-wire granuloma cellularity and regulatory networks to promote immunomodulation following *Mtb* reinfection

Joshua D. Bromley, Sharie Keanne C. Ganchua, Sarah K. Nyquist, Pauline Maiello, Michael Chao, H. Jacob Borish, Mark Rodgers, Jaime Tomko, Kara Kracinovsky, Douaa Mugahid, Son Nguyen, Qianchang Dennis Wang, Jacob M. Rosenberg, Edwin C. Klein, Hannah P. Gideon, Roisin Floyd-O'Sullivan, Bonnie Berger, Charles A. Scanga, Philana Ling Lin, Sarah M. Fortune, Alex K. Shalek, and JoAnne L. Flynn

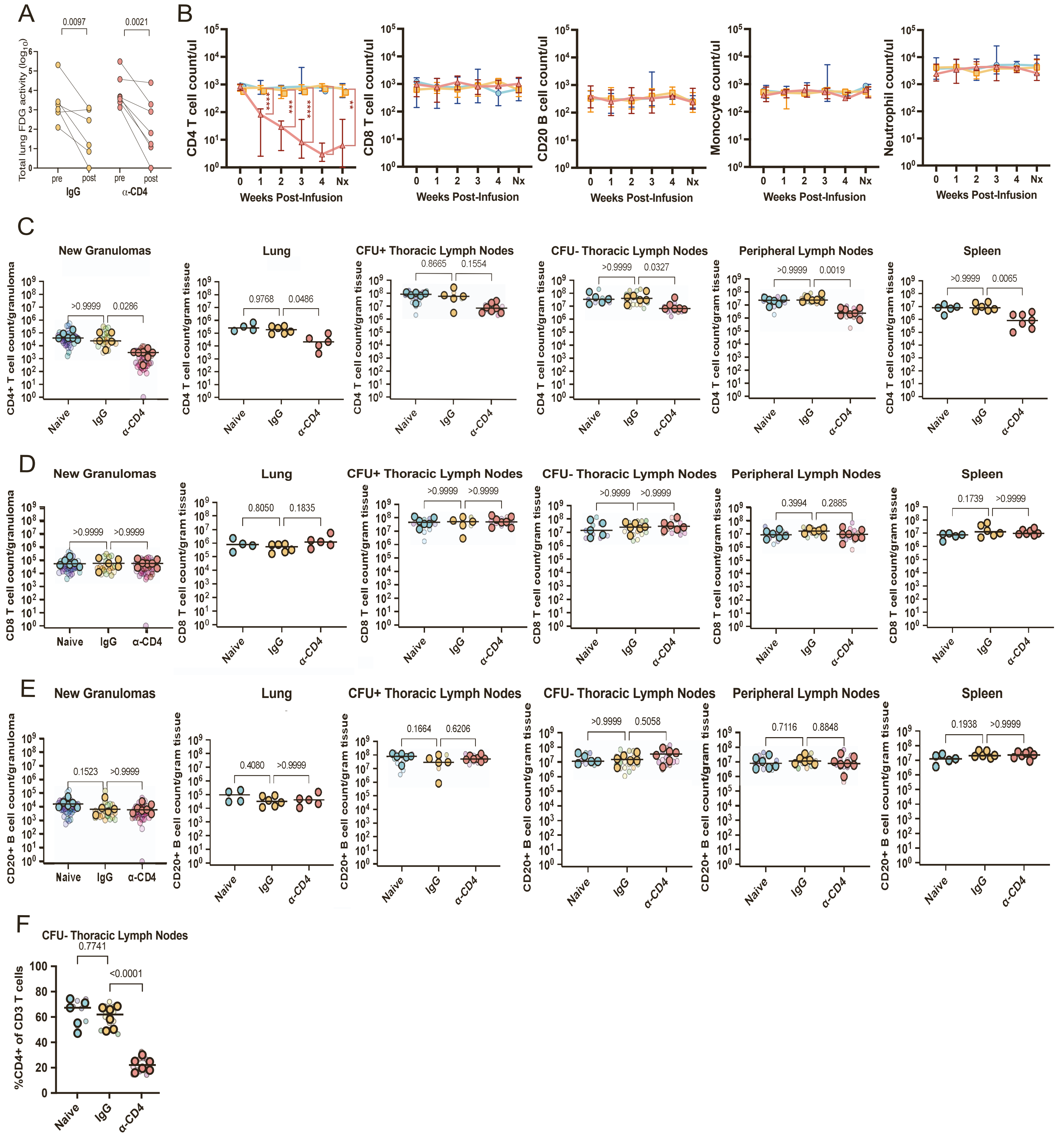
A
Total lung FDG activity (log10)
0.0097
0.0021
pre
post
IgG
α-CD4
B
CD4 T cell count/ul
CD8 T cell count/ul
CD20 B cell count/ul
Monocyte count/ul
Neutrophil count/ul
Weeks Post-Infusion
0 1 2 3 4 Nx
****
***
****
**
C
New Granulomas
Lung
CFU+ Thoracic Lymph Nodes
CFU- Thoracic Lymph Nodes
Peripheral Lymph Nodes
Spleen
CD4+ T cell count/granuloma
CD4 T cell count/gram tissue
Naive
IgG
α-CD4
>0.9999
0.0286
0.9768
0.0486
0.8665
0.1554
>0.9999
0.0327
>0.9999
0.0019
>0.9999
0.0065
D
New Granulomas
Lung
CFU+ Thoracic Lymph Nodes
CFU- Thoracic Lymph Nodes
Peripheral Lymph Nodes
Spleen
CD8 T cell count/granuloma
CD8 T cell count/gram tissue
>0.9999
>0.9999
0.8050
0.1835
>0.9999
>0.9999
>0.9999
>0.9999
0.3994
0.2885
0.1739
>0.9999
E
New Granulomas
Lung
CFU+ Thoracic Lymph Nodes
CFU- Thoracic Lymph Nodes
Peripheral Lymph Nodes
Spleen
CD20+ B cell count/granuloma
CD20+ B cell count/gram tissue
0.1523
>0.9999
0.4080
>0.9999
0.1664
0.6206
>0.9999
0.5058
0.7116
0.8848
0.1938
>0.9999
F
CFU- Thoracic Lymph Nodes
%CD4+ of CD3 T cells
0.7741
<0.0001
Naive
IgG
α-CD4

Figure S1. Cellular frequencies across anatomic compartments, related to Figure 1. (A) Total lung FDG activity pre- and post-HRZE drug treatment. Two-way ANOVA with Bonferroni's multiple comparisons test. Dots with connected lines represent individual animals. Number of animals: IgG n=6, αCD4 n=7. **(B)** Absolute counts (count/uL) of $CD4^+$ T cells, $CD8^+$ T cells, $CD20^+$ B cells, monocytes, and neutrophils post-antibody infusion in peripheral blood. Median and range shown; mixed-effects model with Dunnett's multiple comparisons test (** p<0.01, ***, p<0.001, **** p<0.0001). Number of animals: Naïve n=6, IgG n=6, αCD4 n=7. **(C)** Absolute counts (count/mL) of $CD4^+$ T cells in new granulomas (Animals: Naïve=6, IgG=5, αCD4 n=7; Granulomas: Naïve n=37, IgG n=21, αCD4 n=39), uninvolved lung (Animals: Naïve n=4, IgG n=6, αCD4 n=5), CFU^+ thoracic lymph nodes (Animals: Naïve n=6, IgG n=5, αCD4 n=7; LN: Naïve n=14, IgG n=6, αCD4 n=11), CFU^- thoracic lymph nodes (Animals: Naïve n=5, IgG n=6, αCD4 n=6; LN: Naïve n=8, IgG n=17, αCD4 n=13), peripheral lymph nodes (Animals: Naïve n=6, IgG n=6, αCD4 n=7; LN: Naïve n=11, IgG n=12, αCD4 n=13), and spleen (Animals: Naïve n=5, IgG n=6, αCD4 n=7). **(D)** Absolute counts (count/mL) of $CD8^+$ T cells in new granulomas (Animals: Naïve n=6, IgG n=5, αCD4 n=7; Granulomas: Naïve n=37 IgG n=21, αCD4 n=39), uninvolved lung (Animals: Naïve n=4, IgG n=6, αCD4 n=5), CFU^+ thoracic lymph nodes (Animals: Naïve n=6, IgG n=5, αCD4 n=7; LN: Naïve n=14, IgG n=6, αCD4 n=11) and CFU^- thoracic lymph nodes (Animals: Naïve n=5, IgG n=6, αCD4 n=6; LN: Naïve n=8, IgG n=17, αCD4 n=13), peripheral lymph nodes (Animals: Naïve n=6, IgG n=6, αCD4 n=7; LN: Naïve n=11, IgG n=12, αCD4 n=13), and spleen (Animals: Naïve n=5, IgG n=6, αCD4 n=7). **(E)** Absolute counts (count/mL) of $CD20^+$ B cells in new granulomas (Animals: Naïve n=6, IgG n=5, αCD4 n=7; Granulomas: Naïve n=37 IgG n=20, αCD4 n=39), uninvolved lung (Animals: Naïve n=4, IgG n=6, αCD4 n=5), CFU^+ thoracic lymph nodes (Animals: Naïve n=6, IgG n=5, αCD4 n=7; LN: Naïve n=14, IgG n=6, αCD4 n=11), CFU^- thoracic lymph nodes (Animals: Naïve n=5, IgG n=6, αCD4 n=6; LN: Naïve n=8, IgG n=17, αCD4 n=13), peripheral lymph nodes (Animals: Naïve n=6, IgG n=6, αCD4 n=7; LN: Naïve n=11, IgG n=12, αCD4 n=13), and spleen (Animals: Naïve n=5, IgG n=6, αCD4 n=7). **(F)** Fraction of $CD3^+$, $CD4^+$ cells from CFU^- thoracic lymph nodes resected at necropsy. (Animals: Naïve=5, IgG=6, αCD4 n=6; LN: Naïve=8, IgG=17, αCD4 n=13) One-way ANOVA with Dunnett's multiple comparisons test. (C-F) Transparent smaller dots represent granulomas or lymph nodes, colored by animal. Larger dots represent mean per animal and lines represent medians. Kruskal-Wallis with Dunn's multiple comparisons test adjusted p-values reported.

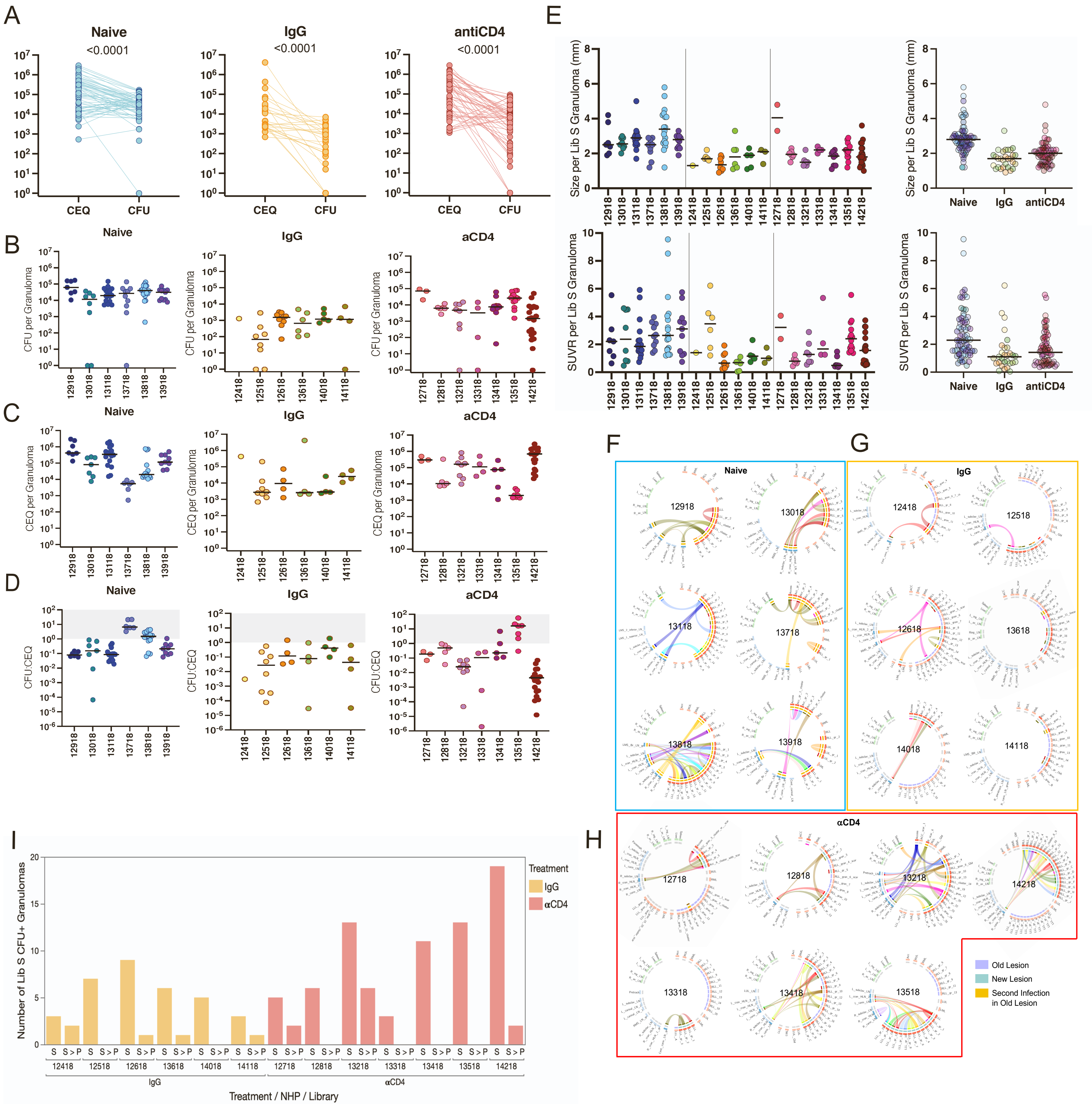

A
Naive
<0.0001
IgG
<0.0001
antiCD4
<0.0001
CEQ
CFU
B
Naive
IgG
aCD4
CFU per Granuloma
12918 13018 13118 13718 13818 13918
12418 12518 12618 13618 14018 14118
12718 12818 13218 13318 13418 13518 14218
C
Naive
IgG
aCD4
CEQ per Granuloma
D
Naive
IgG
aCD4
CFU:CEQ
E
Size per Lib S Granuloma (mm)
SUVR per Lib S Granuloma
Naive
IgG
antiCD4
F
Naive
12918
13018
13118
13718
13818
13918
G
IgG
12418
12518
12618
13618
14018
14118
H
αCD4
12718
12818
13218
14218
13318
13418
13518
Old Lesion
New Lesion
Second Infection in Old Lesion
I
Number of Lib S CFU+ Granulomas
Treatment
IgG
αCD4
S
S > P
Treatment / NHP / Library

Figure S2. *Mtb* CFU, CEQ, CFU:CEQ, and dissemination, related to Figure 2. (A) CEQ (left), and CFU (right). Individual dots represent individual TB granulomas. Linkages depict CEQ and CFU within the same lesion. Wilcoxon matched-pairs signed rank test. Number of granulomas: Naïve CEQ n=59, CFU n=70; IgG CEQ n=25, CFU n=34; αCD4 CEQ n=51, CFU n=65. **(B)** *Mtb* CFU per granuloma. Integers on the x-axis represent an individual macaque. Colored dots are individual granuloma. Lines represent median per animal. Granuloma numbers for animals (from left to right): Naïve n=(7, 8, 16, 10, 18, 11); IgG n=(1, 8, 10, 6, 5, 4); αCD4 n=(3, 6, 8, 4, 11, 14, 19). **(C)** *Mtb* CEQ per granuloma. Integers on the x-axis represent an individual macaque. Colored dots are individual granuloma. Granuloma numbers for animals (from left to right): Naïve n=(7, 7, 16, 6, 14, 9); IgG n=(1, 8, 4, 4, 4, 4); αCD4 n=(3, 5, 8, 4, 5, 7, 19). **(D)** CFU:CEQ per granuloma. Integers on the x-axis represent an individual macaque. Colored dots are individual granuloma. Lines represent median per animal. Granuloma numbers for animals (from left to right): Naïve n=(7, 7, 16, 6, 14, 9); IgG n=(1, 8, 4, 4, 4, 4); αCD4 n=(3, 5, 8, 4, 5, 7, 19). **(E)** Size in mm per library S granuloma (as measured on CT) by monkey and by treatment group. Granuloma numbers for animals (from left to right): Naïve n=70 (7, 8, 16, 10, 18, 11); IgG n=29 (1, 6, 8, 6, 5, 3); αCD4 n=61 (2, 6, 7, 4, 8, 14, 20). FDG Avidity (SUVR) per library S granuloma by monkey and by treatment group. Granuloma numbers for animals (from left to right): Naïve n=69 (7, 8, 16, 10, 17, 11); IgG n=29 (1, 6, 8, 6, 5, 3); αCD4 n=61 (2, 6, 7, 4, 8, 14, 20). Each symbol represents an individual granuloma symbols are colored by animal. Median lines are shown both within animal and treatment groups. (**F-H**) Circos plots depicting *Mtb* strains shared between anatomical sites in naïve (E), reinfected (F) or reinfected with CD4 depletion (G) animals. Individual circos plots represent a single macaque, labeled with their study ID. In the plots, each wedge represents a distinct tissue site that was sampled and/or sequenced for barcodes. There are three tracks in each plot. The outer ring defines tissue samples as being from lungs (red), thoracic lymph nodes (blue) or distal extrapulmonary sites (green). Lighter shades represent tissues that were plated for *Mtb* but were sterile. The middle ring represents lesions that were detected by PET-CT during primary infection with the first *Mtb* library ('old lesion'), new lesions detected by PET-CT after re-infection ('new lesion'), or new lesions (as defined by Library S

barcode sequencing) that were also found at sites where old lesions had previously formed (detected by PET-CT). The innermost ring represents distinct barcodes found in each tissue, where each unique barcoded *Mtb* strain (from the secondary *Mtb* library) is given a different color. Tissues that share the same *Mtb* strain by sequencing are linked by ribbons. Lung tissues are further grouped by lobe, abbreviated as follows: RUL, right upper lobe; RML, right middle lobe; RLL, right lower lobe; LUL, left upper lobe; LML, left middle lobe; LLL, left lower lobe; Acc, accessory lobe. (**I**) Bars represent either number of total CFU+ Library S granulomas ("S") or the number of the subset of library S found in old (established as library P) granulomas ("S > P") within each animal. Number of Library S in old granulomas ranged from 0 to 2 in IgG animals and 0 to 6 in CD4-depleted animals.

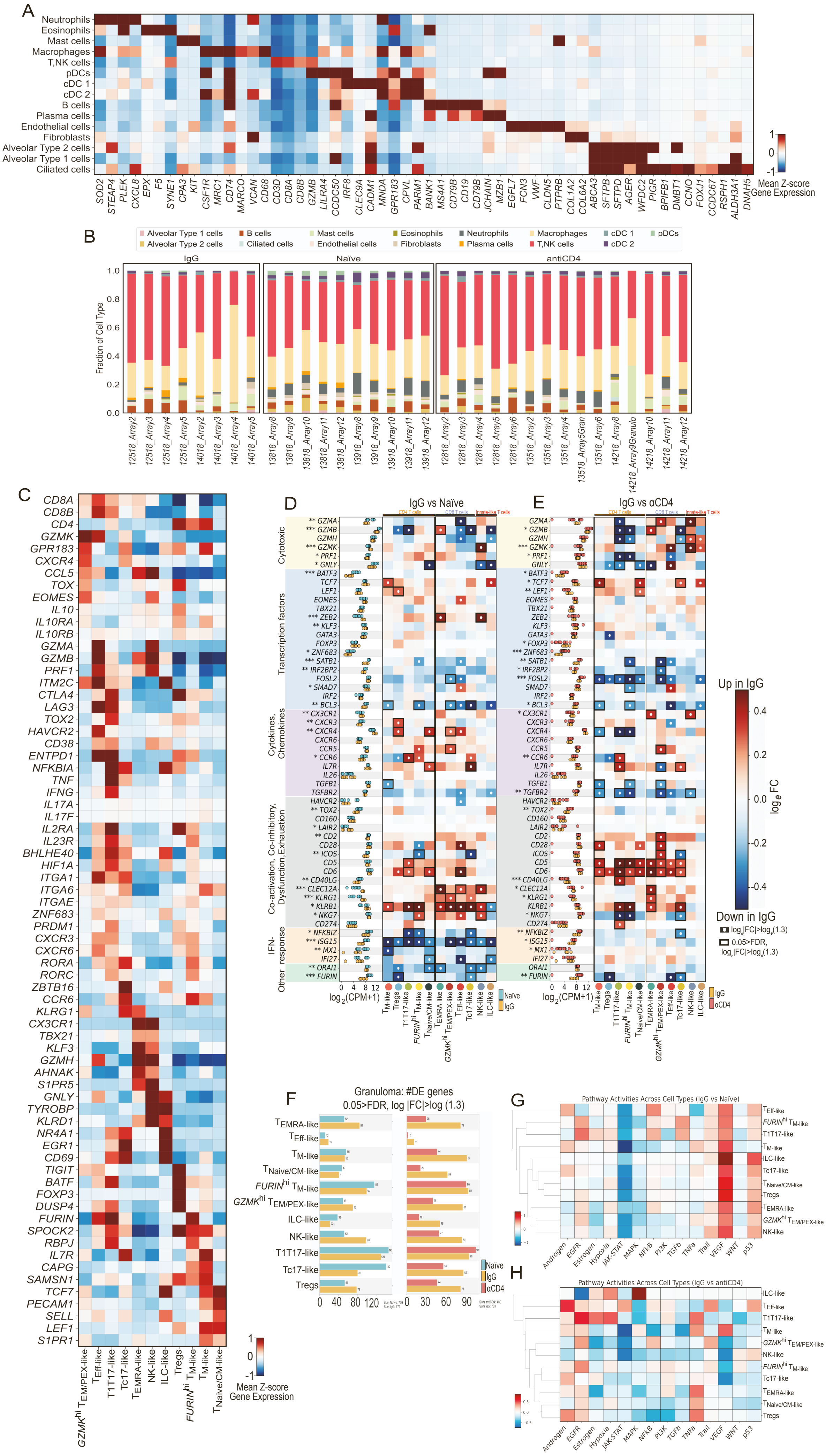
A
Neutrophils
Eosinophils
Mast cells
Macrophages
T,NK cells
pDCs
cDC 1
cDC 2
B cells
Plasma cells
Endothelial cells
Fibroblasts
Alveolar Type 2 cells
Alveolar Type 1 cells
Ciliated cells
SOD2 STEAP4 PLEK CXCL8 EPX F5 SYNE1 CPA3 KIT CSF1R MRC1 CD74 MARCO VCAN CD68 CD3D CD8A CD8B GZMB LILRA4 CCDC50 IRF8 CLEC9A CADM1 MNDA GPR183 CPVL PARM1 BANK1 MS4A1 CD79B CD19 CD79B JCHAIN MZB1 EGFL7 FCN3 VWF CLDN5 PTPRB COL1A2 COL6A2 ABCA3 SFTPB SFTPD AGER WFDC2 PIGR BPIFB1 DMBT1 CCNO FOXJ1 CCDC67 RSPH1 ALDH3A1 DNAH5
Mean Z-score Gene Expression
B
Alveolar Type 1 cells
Alveolar Type 2 cells
B cells
Ciliated cells
Mast cells
Endothelial cells
Eosinophils
Fibroblasts
Neutrophils
Plasma cells
Macrophages
T,NK cells
cDC 1
cDC 2
pDCs
IgG
Naïve
antiCD4
Fraction of Cell Type
12518_Array2 12518_Array3 12518_Array4 12518_Array5 14018_Array2 14018_Array3 14018_Array4 14018_Array5 13818_Array8 13818_Array9 13818_Array10 13818_Array11 13818_Array12 13918_Array8 13918_Array9 13918_Array10 13918_Array11 13918_Array12 12818_Array2 12818_Array3 12818_Array4 12818_Array5 12818_Array6 13518_Array2 13518_Array3 13518_Array4 13518_Array5Gran 13518_Array6 14218_Array8 14218_Array9Granulo 14218_Array10 14218_Array11 14218_Array12
C
CD8A CD8B CD4 GZMK GPR183 CXCR4 CCL5 TOX EOMES IL10 IL10RA IL10RB GZMA GZMB PRF1 ITM2C CTLA4 LAG3 TOX2 HAVCR2 CD38 ENTPD1 NFKBIA TNF IFNG IL17A IL17F IL2RA IL23R BHLHE40 HIF1A ITGA1 ITGA6 ITGAE ZNF683 PRDM1 CXCR3 CXCR6 RORA RORC ZBTB16 CCR6 KLRG1 CX3CR1 TBX21 KLF3 GZMH AHNAK S1PR5 GNLY TYROBP KLRD1 NR4A1 EGR1 CD69 TIGIT BATF FOXP3 DUSP4 FURIN SPOCK2 RBPJ IL7R CAPG SAMSN1 TCF7 PECAM1 SELL LEF1 S1PR1
GZMKhi TEM/PEX-like
TEff-like
T1T17-like
Tc17-like
TEMRA-like
NK-like
ILC-like
Tregs
FURINhi TM-like
TM-like
TNaive/CM-like
Mean Z-score Gene Expression
D
IgG vs Naïve
CD4 T cells
CD8 T cells
Innate-like T cells
Cytotoxic
Transcription factors
Cytokines, Chemokines
Co-activation, Co-inhibitory, Dysfunction,Exhaustion
IFN-response
Other
log2(CPM+1)
Naïve
IgG
E
IgG vs αCD4
IgG
αCD4
Up in IgG
Down in IgG
loge FC
|log2FC|>log2(1.3)
0.05>FDR, log2|FC|>log2(1.3)
TM-like Tregs T1T17-like FURINhi TM-like TNaive/CM-like TEMRA-like GZMKhi TEM/PEX-like TEff-like Tc17-like NK-like ILC-like
F
Granuloma: #DE genes
0.05>FDR, log |FC|>log (1.3)
TEMRA-like
TEff-like
TM-like
TNaive/CM-like
FURINhi TM-like
GZMKhi TEM/PEX-like
ILC-like
NK-like
T1T17-like
Tc17-like
Tregs
Naïve
IgG
αCD4
G
Pathway Activities Across Cell Types (IgG vs Naïve)
H
Pathway Activities Across Cell Types (IgG vs antiCD4)
Androgen EGFR Estrogen Hypoxia JAK-STAT MAPK NFkB PI3K TGFb TNFa Trail VEGF WNT p53

Figure S3. Coarse-grain cell types and T, NK cell diversity in the TB granuloma, related to Figures 3, 4. (A) Heatmap depicting gene expression profiles (mean z-score) of coarse cell-type markers. Marker genes are depicted on the x-axis, and coarse-cell types on the y-axis. **(B)** Coarse-grain cell type frequencies in the TB granuloma (naïve (left), IgG (middle), αCD4 (right)). Individual bars represent a single TB granuloma profiled using Seq-Well S^3. **(C)** Heatmap depicting gene expression profiles (mean z-score) of T,NK subpopulation markers, as well as select transcription factors, cytokine and chemokine receptors, co-inhibitory and co-activation markers. (**D**) T, NK cell pseudobulk Log_2(CPM+1) for naïve (light blue), IgG (yellow), and αCD4 (red) NHP granulomas (*** p<0.001,** p<0.01, *p<0.05; Wilcoxon rank-sum test). Heatmap depicting log_eFC of lineage markers, cytolytic molecules, select transcription factors, immunoregulatory molecules, and chemokines, and cytokines (rows) for each cell type (columns). White circles indicate log_e|FC|> log_e(1.3), relative to naïve or αCD4 granulomas. Black rectangles indicate 0.05>FDR and log_e|FC|> log_e(1.3), relative to naïve or αCD4 granulomas. **(F)** Number of differentially expressed genes among T, NK cell subpopulations with 0.05>FDR and log_e|FC|> log_e(1.3), naïve (light blue), IgG (yellow), and αCD4 (red). (**G**) Heatmap depicting PROGENy (derived from pseudobulk matrices) enrichment scores. Red indicates a pathway is enriched in IgG, whereas blue indicates it is downregulated (i.e., upregulated in naïve). (**H**) Heatmap same as (G) but depicting IgG (red) vs αCD4 (blue).

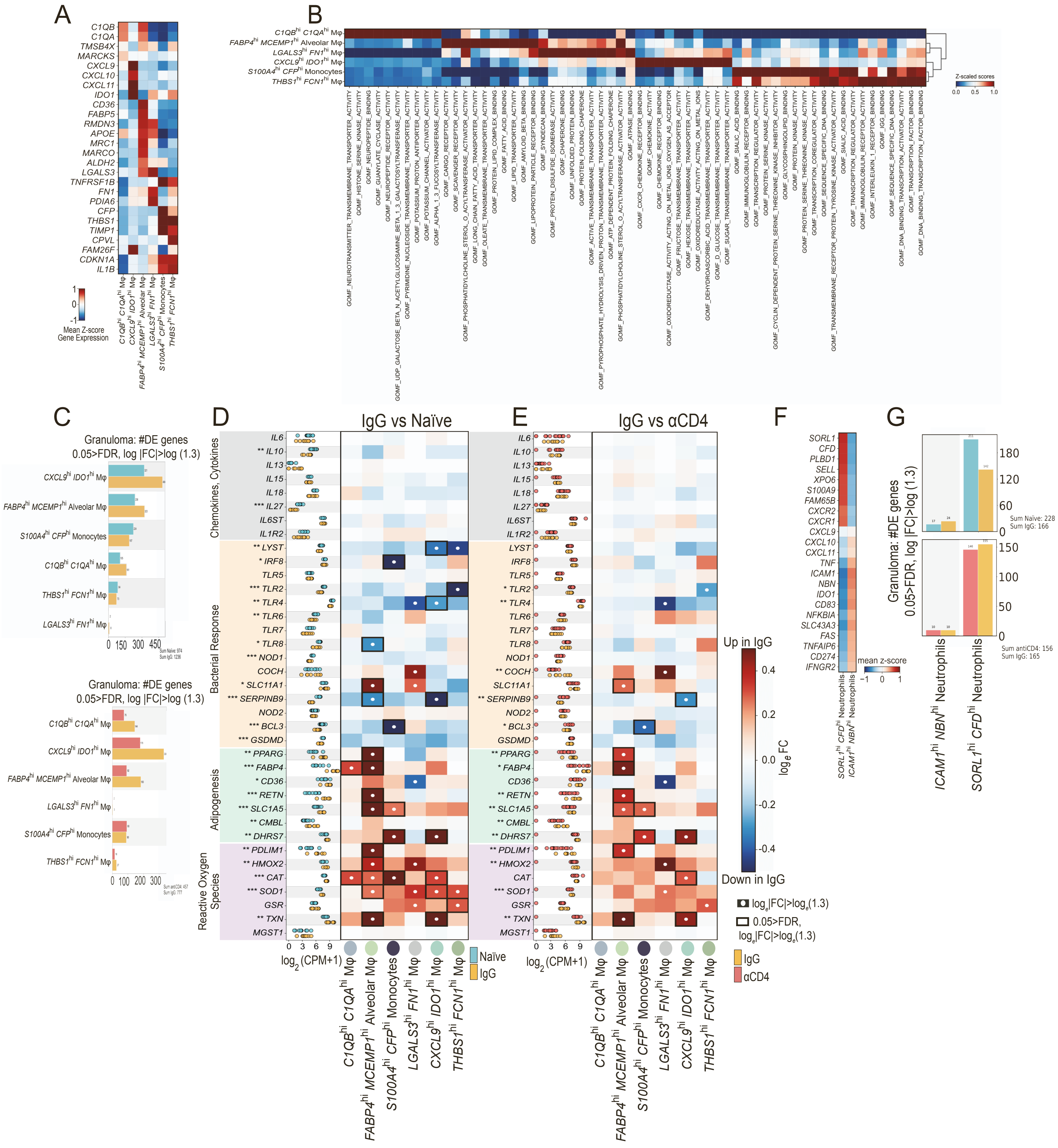

A
C1QB
C1QA
TMSB4X
MARCKS
CXCL9
CXCL10
CXCL11
IDO1
CD36
FABP5
RMDN3
APOE
MRC1
MARCO
ALDH2
LGALS3
TNFRSF1B
FN1
PDIA6
CFP
THBS1
TIMP1
CPVL
FAM26F
CDKN1A
IL1B
Mean Z-score Gene Expression
B
Z-scaled scores
C
Granuloma: #DE genes
0.05>FDR, log |FC|>log (1.3)
Sum Naïve: 974
Sum IgG: 1236
Sum antiCD4: 457
Sum IgG: 777
D
IgG vs Naïve
E
IgG vs αCD4
Chemokines, Cytokines
Bacterial Response
Adipogenesis
Reactive Oxygen Species
IL6
IL10
IL13
IL15
IL18
IL27
IL6ST
IL1R2
LYST
IRF8
TLR5
TLR2
TLR4
TLR6
TLR7
TLR8
NOD1
COCH
SLC11A1
SERPINB9
NOD2
BCL3
GSDMD
PPARG
FABP4
CD36
RETN
SLC1A5
CMBL
DHRS7
PDLIM1
HMOX2
CAT
SOD1
GSR
TXN
MGST1
log2 (CPM+1)
Naïve
IgG
αCD4
Up in IgG
Down in IgG
loge FC
loge|FC|>loge(1.3)
0.05>FDR, loge|FC|>loge(1.3)
C1QBhi C1QAhi Mφ
FABP4hi MCEMP1hi Alveolar Mφ
S100A4hi CFPhi Monocytes
LGALS3hi FN1hi Mφ
CXCL9hi IDO1hi Mφ
THBS1hi FCN1hi Mφ
F
SORL1
CFD
PLBD1
SELL
XPO6
S100A9
FAM65B
CXCR2
CXCR1
CXCL9
CXCL10
CXCL11
TNF
ICAM1
NBN
IDO1
CD83
NFKBIA
SLC43A3
FAS
TNFAIP6
CD274
IFNGR2
mean z-score
SORL1hi CFDhi Neutrophils
ICAM1hi NBNhi Neutrophils
G
Granuloma: #DE genes
0.05>FDR, log |FC|>log (1.3)
Sum Naïve: 228
Sum IgG: 166
Sum antiCD4: 156
Sum IgG: 165
ICAM1hi NBNhi Neutrophils
SORL1hi CFDhi Neutrophils

Figure S4. Monocyte-derived gene programming and neutrophil heterogeneity, related to Figures 5, 6. (A) Heatmap depicting gene expression profiles (mean z-score) of monocyte-derived subpopulation markers. Genes depicted on y axis and monocyte-derived subsets on the x axis. **(B)** Enriched GO Molecular Function (2021) pathways among monocyte-derived subsets (one macrophages subset vs rest (i.e., all other macrophage populations); Z-scaled scores). Calculated using Decoupler. (**C)** Number of differentially expressed genes (calculated using MAST) among monocyte-derived subpopulations with 0.05>FDR and $\log_e|FC|> \log_e(1.3)$, naïve (light blue), IgG (yellow), and αCD4 (red). **(C-D)** Monocyte-derived pseudobulk $\text{Log}_2(\text{CPM+1})$ for naïve (light blue), IgG (yellow), and αCD4 (red) NHP granulomas (*** $p<0.001$,** $p<0.01$, *$p<0.05$; Wilcoxon rank-sum test). Heatmap depicting $\log_e FC$ of select chemokines and cytokines, bacterial response genes[S1], adipogenesis, and reactive oxygen species (rows) for each cell type (columns) in NHP granulomas, IgG vs naïve (C) or IgG vs αCD4 lesions (D). White circles indicate $\log_e|FC|> \log_e(1.3)$, relative to naïve or αCD4 granulomas. Black rectangles indicate 0.05>FDR and $\log_e|FC|> \log_e(1.3)$, relative to naïve or αCD4 granulomas. **(E)** Heatmap depicting neutrophil subpopulation marker gene expression profiles (mean z-score). Genes depicted on y axis and monocyte-derived subsets on the x axis. **(F)** Number of differentially expressed genes among neutrophil subpopulations with 0.05>FDR and $\log_e|FC|> \log_e(1.3)$, naïve (light blue), IgG (yellow), and αCD4 (red).

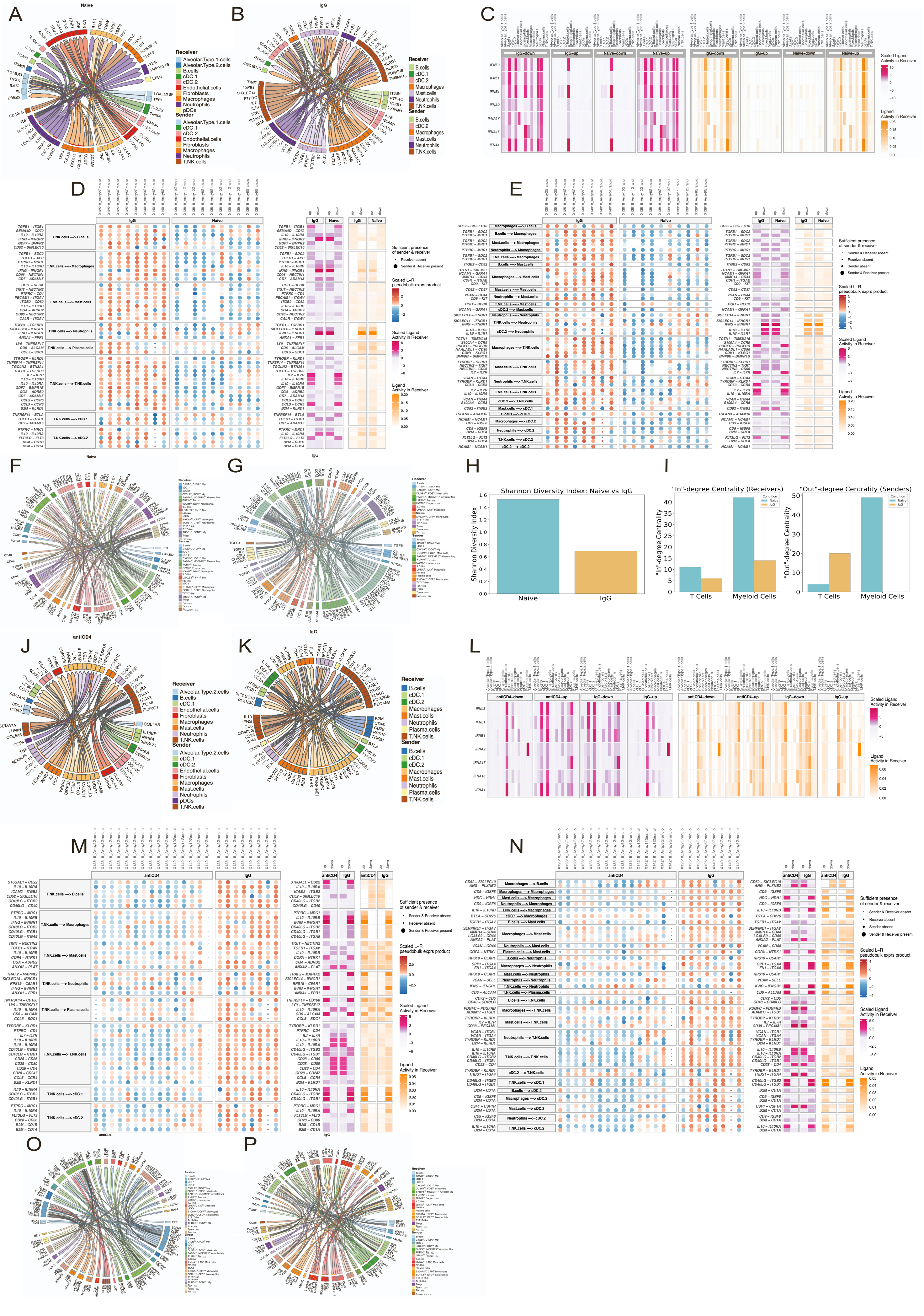

A
Naive
B
IgG
C
D
E
F
Naive
G
IgG
H
Shannon Diversity Index: Naive vs IgG
Shannon Diversity Index
Naive
IgG
I
"In"-degree Centrality (Receivers)
"In"-degree Centrality
"Out"-degree Centrality (Senders)
"Out"-degree Centrality
T Cells
Myeloid Cells
J
antiCD4
K
IgG
L
M
N
O
antiCD4
P
IgG

Figure S5. Cell-cell and ligand-receptor interactions during *Mtb* reinfection, related to Figure 7. (A) Circos plot depicting differential network interactions among coarse-grain cell type annotations in naïve granulomas (naïve vs IgG). Ribbon arrows indicate directionality (sender to receiver) populations. The outer edge color among senders denotes the sender cell type, with the inner edge color representing the receiver cell type. Circos plot depicts the 50 top-prioritized linkages. **(B)** Circos plot depicting differential cell-cell interactions among IgG lesions – same as in (A) but comparing IgG to naïve. **(C)** Heatmap depicting type 1 interferon ligand-activity among coarse cell types depicting both scaled ligand activity and ligand activity in receiver cell types **(D)** Heatmap (left) depiction of scaled ligand-receptor expression among the top 50 prioritized linkages among T, NK cells – as putative "sender" populations – from IgG granulomas, relative to naïve. Dot size represents L-R presence or absence. Columns represent individual granulomas. Heatmap split by NHP group. Heatmaps (right) are representations of the scaled ligand activity and ligand activity among putative receiver populations. **(E)** Heatmap similar to (D) but depicting the top 50 prioritized linkages among all coarse-grain cell types (all cell types depicted as "sender" or "receiver," if among top 50 DE linkages). **(F)** Circos plot showing differential network interactions among immune cell subpopulations in naïve granulomas (naïve vs IgG). Top 100 prioritized linkages are plotted. **(G)** Circos plot depicting differential cell-cell interactions among all immune cell subpopulations IgG vs naïve. **(H)** Shannon Diversity Index among *IL10* "senders" – a larger index represents increased diversity. **(I)** Degree Centrality among *IL10* "senders" and "receivers" – elevated centrality indicates more "incoming" and "outgoing" communication events. Myeloid cell centrality is among all cellular subsets of myeloid lineage. (**J**) Differential cell-cell interactions among αCD4 lesions (αCD4 vs IgG). **(K)** Differential cell-cell interactions among IgG lesions (IgG vs αCD4). **(L)** Heatmap depicting type 1 interferon ligand-activity among coarse cell types, IgG vs αCD4. **(M)** Heatmap similar to (D) but displaying the top 50 prioritized linkages among T, NK cells (only T, NK cells are depicted as "sender" populations) from IgG granulomas, relative to αCD4. **(N)** Heatmap similar to (D) but depicting the top 50 prioritized linkages among all coarsely annotated cell types from IgG granulomas relative to αCD4. **(O)** Circos plot depicting differential network interactions among

immune cell subpopulations in αCD4 granulomas (αCD4vs IgG). Top 100 prioritized linkages are plotted. **(P)** Differential cell-cell interactions among all immune cell subpopulations IgG vs αCD4.

Supplemental references

[S1]. Yao, Y., Jeyanathan, M., Haddadi, S., Barra, N.G., Vaseghi-Shanjani, M., Damjanovic, D., Lai, R., Afkhami, S., Chen, Y., Dvorkin-Gheva, A., et al. (2018). Induction of Autonomous Memory Alveolar Macrophages Requires T Cell Help and Is Critical to Trained Immunity. Cell *175*, 1634–1650.e17. https://doi.org/10.1016/j.cell.2018.09.042.
