# Supplementary material for: CD4+ T cells re-wire granuloma cellularity and regulatory networks to promote immunomodulation following Mtb reinfection
Source: Immunity. 2024 Oct 8;57(10):2380–2398.e6. doi: 10.1016/j.immuni.2024.08.002 (PMC11466276; doi:10.1016/j.immuni.2024.08.002)
Supplement: Document S2. Article plus supplemental information [file mmc11.pdf]

Article

Immunity

CD4⁺ T cells re-wire granuloma cellularity and regulatory networks to promote immunomodulation following *Mtb* reinfection

Graphical abstract

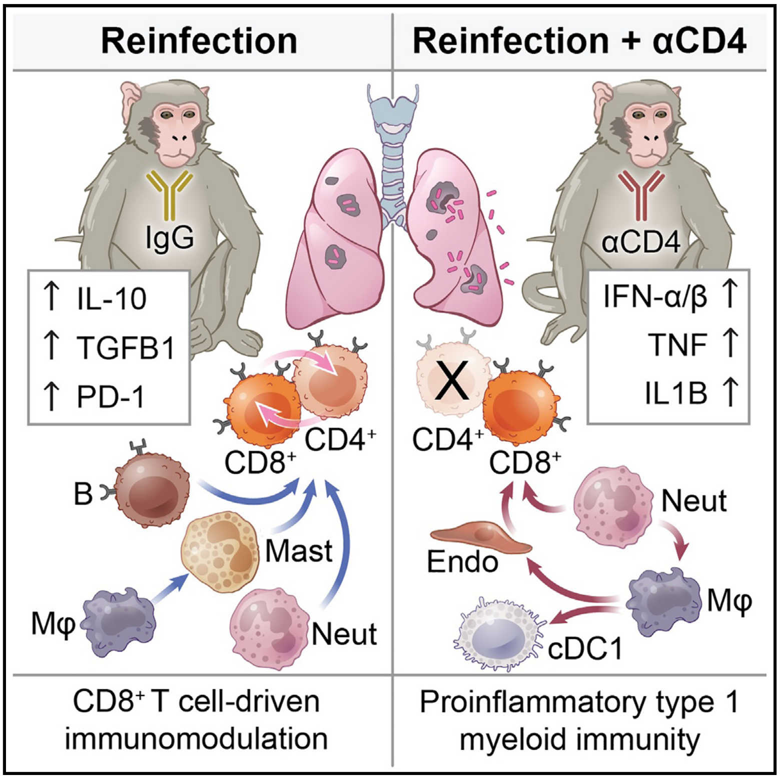

Highlights

- $CD4^+$ T cells are required for protection against *Mtb* reinfection in macaques
- *Mtb* reinfection promotes immuno-modulatory $CD8^+$ T cell-biased immunity
- Myeloid-derived cells downregulate gene networks implicated in TB susceptibility
- Self-reinforcing cellular circuits balance host immunity in reinfection granulomas

Authors

Joshua D. Bromley, Sharie Keanne C. Ganchua, Sarah K. Nyquist, ..., Sarah M. Fortune, Alex K. Shalek, JoAnne L. Flynn

Correspondence

sfortune@hsph.harvard.edu (S.M.F.), shalek@mit.edu (A.K.S.), joanne@pitt.edu (J.L.F.)

In brief

Th1 $CD4^+$ T cells mediate protective anti-*Mtb* immunity across biological systems and organisms. Bromley, Ganchua, et al. demonstrate that $CD4^+$ T cells regulate immune tone in TB granulomas and are necessary for immune recall and protection against reinfection. Following reinfection, $CD4^+$ T cells facilitate the development of a growth restrictive niche via the induction of immuno-modulatory genes and cellular interaction networks.

Bromley et al., 2024, Immunity *57*, 2380–2398
October 8, 2024 © 2024 The Authors. Published by Elsevier Inc.
https://doi.org/10.1016/j.immuni.2024.08.002

CellPress

CellPress
OPEN ACCESS

Immunity

Article

CD4⁺ T cells re-wire granuloma cellularity and regulatory networks to promote immunomodulation following *Mtb* reinfection

Joshua D. Bromley,[1,2,3,4,13] Sharie Keanne C. Ganchua,[5,6,13] Sarah K. Nyquist,[1,2,3,8] Pauline Maiello,[5] Michael Chao,[1,9] H. Jacob Borish,[5,6] Mark Rodgers,[5,6] Jaime Tomko,[5,6] Kara Kracinovsky,[5,6] Douaa Mugahid,[1,9] Son Nguyen,[1,2,3] Qianchang Dennis Wang,[2] Jacob M. Rosenberg,[1,9] Edwin C. Klein,[7] Hannah P. Gideon,[5,6] Roisin Floyd-O'Sullivan,[1,2,3] Bonnie Berger,[8] Charles A. Scanga,[5,6] Philana Ling Lin,[6,10] Sarah M. Fortune,[1,9,14,15,*] Alex K. Shalek,[1,2,3,11,12,14,15,*] and JoAnne L. Flynn[5,6,13,14,15,16,*]

[1]Ragon Institute of MGH, MIT, and Harvard, Cambridge, MA, USA
[2]Institute for Medical Engineering and Science (IMES), Massachusetts Institute of Technology, Cambridge, MA, USA
[3]Broad Institute of MIT and Harvard, Cambridge, MA, USA
[4]Graduate Program in Microbiology, Massachusetts Institute of Technology, Cambridge, MA, USA
[5]Department of Microbiology and Molecular Genetics, University of Pittsburgh School of Medicine, Pittsburgh, PA, USA
[6]Center for Vaccine Research, University of Pittsburgh, Pittsburgh, PA, USA
[7]Division of Laboratory Animal Research, University of Pittsburgh, Pittsburgh, PA, USA
[8]Computer Science and Artificial Intelligence Laboratory, Massachusetts Institute of Technology, Cambridge, MA, USA
[9]Department of Immunology and Infectious Diseases, Harvard T.H. Chan School of Public Health, Boston, MA, USA
[10]Department of Pediatrics, UPMC Children's Hospital of Pittsburgh, University of Pittsburgh School of Medicine, Pittsburgh, PA, USA
[11]Department of Chemistry, Massachusetts Institute of Technology, Cambridge, MA, USA
[12]Koch Institute for Integrative Cancer Research, Massachusetts Institute of Technology, Cambridge, MA, USA
[13]These authors contributed equally
[14]These authors contributed equally
[15]Senior author
[16]Lead contact
*Correspondence: sfortune@hsph.harvard.edu (S.M.F.), shalek@mit.edu (A.K.S.), joanne@pitt.edu (J.L.F.)
https://doi.org/10.1016/j.immuni.2024.08.002

SUMMARY

Immunological priming—in the context of either prior infection or vaccination—elicits protective responses against subsequent *Mycobacterium tuberculosis* (*Mtb*) infection. However, the changes that occur in the lung cellular milieu post-primary *Mtb* infection and their contributions to protection upon reinfection remain poorly understood. Using clinical and microbiological endpoints in a non-human primate reinfection model, we demonstrated that prior *Mtb* infection elicited a long-lasting protective response against subsequent *Mtb* exposure and was CD4⁺ T cell dependent. By analyzing data from primary infection, reinfection, and reinfection-CD4⁺ T cell-depleted granulomas, we found that the presence of CD4⁺ T cells during reinfection resulted in a less inflammatory lung milieu characterized by reprogrammed CD8⁺ T cells, reduced neutrophilia, and blunted type 1 immune signaling among myeloid cells. These results open avenues for developing vaccines and therapeutics that not only target lymphocytes but also modulate innate immune cells to limit tuberculosis (TB) disease.

INTRODUCTION

Management of the tuberculosis (TB) epidemic is limited by the lack of a robust vaccine that protects against *Mycobacterium tuberculosis* (*Mtb*) infection and disease progression. Bacillus Calmette-Guérin (BCG) remains the only licensed TB vaccine. It offers protection against severe miliary and meningeal infections in pediatric TB but fails to confer robust protection against infection or TB disease in adults.[1,2] Regardless of BCG vaccination status, the majority of infected individuals can control *Mtb* bacilli naturally and experience asymptomatic infection (clinically classified as latent TB infection [LTBI]). Only ~5%–10% experience overt clinical manifestations of disease.[3] In TB endemic regions—where people are likely repeatedly exposed—the incidence rate of recurrent TB disease, by either relapse or reinfection, after successful treatment with antibiotics is 18 (China) and 14.6 (Spain) times higher than the incidence rate of initial TB disease in the general population.[4–6] However, a retrospective epidemiological meta-analyses of healthcare professionals reports a 79% lower risk of developing active TB in LTBI individuals

 © 2024 The Authors. Published by Elsevier Inc.
This is an open access article under the CC BY license (http://creativecommons.org/licenses/by/4.0/).

CellPress
OPEN ACCESS

after re-exposure to *Mtb* compared with uninfected individuals.[7] This observation is further bolstered by findings from non-human primate (NHP) and murine models, which demonstrate that concomitant immunity (immunological memory conferred by concurrent *Mtb* infection) provides robust protection against *Mtb* reinfection and that this protection persists to some extent after drug treatment.[8–10]

Several factors could explain variation in a host's ability to control TB following *Mtb* reinfection, including intrinsic differences in host susceptibility, differences in quality of memory immune responses, TB-related structural lung disease, and pathogen characteristics.[11–14] Since prior *Mtb* infection provides protection against reinfection in NHPs, we can use this model to dissect the roles that key immune cell subsets play in protection.[8] Here, we sought to define the roles and functions of $CD4^+$ T cells in the setting of reinfection.

The importance of $CD4^+$ T cells for protection from *Mtb* infection and TB disease has been established in humans by observing the devastating effects of HIV on TB disease burden. It is further supported by studies in mice and NHPs, where loss of $CD4^+$ T cells leads to increased pathology, bacterial burden, and reactivation disease.[15–17] However, in NHPs, as in humans, the outcome of *Mtb* infection varies across sites of infection, such that there can be simultaneous sterilization and progression of infection in different granulomas within the same host.[18,19] Likewise, while $CD4^+$ T cell depletion in *Mtb* infection leads to worsened control overall, some lesions are fully sterilized and some animals do well—observations that the current paradigm of protective immunity to *Mtb* cannot explain.[16,20–24]

In this study, we sought to evaluate long-lived immunological reprogramming in pulmonary granulomas after primary and secondary infection and to elucidate the role of $CD4^+$ T cells in protection against reinfection. Using *in vivo* perturbations (reinfection and $CD4^+$ T cell depletion) and a combination of clinical, microbiologic, and high-dimensional single-cell transcriptomic analyses, we characterized intra- and inter-cellular changes associated with infection outcomes within pulmonary granulomas in cynomolgus macaques. Our analyses help to unravel the intricacies of host-pathogen dynamics in TB, providing foundational insights for advancing vaccine research and therapeutic modalities.

RESULTS

Mtb infection in cynomolgus macaques was used to study infection and reinfection

We used antibody-based depletion of $CD4^+$ T cells (hereafter, αCD4) immediately prior to and during reinfection to assess $CD4^+$ T cells effector functions in an immune-primed environment. We compared the outcomes of *Mtb*-barcoded[25] Erdman strains (designated primary [P] and secondary [S] infection libraries) in reinfection in the setting of αCD4 ($n = 7$) to those of an isotype control (immunoglobulin G [IgG] antibody infusion, $n = 6$). We also examined primary infection in naive animals (primary infection library S only, $n = 6$) (Figure 1A; STAR Methods).

Assessment of total lung ^{18}F-fluorodeoxyglucose (FDG) activity pre- and post-HRZE drug treatment indicated that response to treatment was similar in the two drug treatment groups (Figure S1A). Serial PET-computed tomography (CT) enabled identification of newly formed granulomas following *Mtb* re-challenge and antibody infusions (Figure 1B). At necropsy, individual PET-CT scan-matched granulomas, lymph nodes, and all lung lobes were resected and dissociated into single-cell suspensions for quantitative microbiology, flow cytometry, and/or single-cell RNA sequencing (scRNA-seq).

$CD4^+$ T cells were depleted post-infusion (10- to 1,000-fold compared with pre-infusion) in the blood of αCD4 animals up until necropsy; no changes in $CD4^+$ T cell numbers were observed in the naive and IgG cohorts (Figures 1C and S1B). $CD4^+$ T cell depletion in macaques also reduced the number of $CD4^+$ T cells, but not $CD8^+$ T cells or B cells, in tissues, including granulomas and lymph nodes, as compared with macaques that received IgG (Figures 1D–1G and S1C–S1F). This successful depletion enabled us to compare the outcome of reinfection to primary infection (IgG vs. naive) and then assess the impact of $CD4^+$ T cell depletion in reinfection (IgG vs. αCD4).

Reinfection with *Mtb* reduced granuloma formation, as well as bacterial burden and dissemination, in a $CD4^+$ T cell-dependent manner

Analysis of PET-CT scans after secondary infection showed that a similar number of library S^+ granulomas formed in animals receiving IgG as compared with naive and αCD4-treated animals, with a trend ($p = 0.0714$) toward fewer new granulomas in IgG compared with naive animals (Figure 2A). Consistent with our prior data,[8] IgG animals had significantly fewer viable bacteria than those in naive animals, with $CD4^+$ T cell depletion partially abrogating protection against reinfection (Figures 2B, 2E–2H, and S2A). While there was a trend toward lower cumulative bacterial burdens (chromosomal equivalents [CEQs]; an estimate of total live and dead bacilli) in granulomas from IgG NHPs, these differences did not reach statistical significance (Figures 2C and S2B). The same was true for the colony-forming unit (CFU):CEQ ratio[19]—a proxy for bacterial killing within a granuloma (Figures 2D, S2C, and S2D). Histologic analysis of granulomas did not show major differences between naive and IgG, or αCD4 and IgG macaques; however, most granulomas from IgG animals were too small to bisect or did not provide adequate sections for histology. The PET-CT data on library S granulomas supported that IgG granulomas were smaller in size and with reduced FDG avidity, on average, compared with αCD4 granulomas (Figure S2E).

Mtb barcode analysis of samples retrieved at necropsy revealed that previous infection provided enhanced protection against *Mtb* dissemination of the second infection to lymph nodes, and this protection was partially dependent on $CD4^+$ T cells (Figures S2E–S2G). We found a lower percentage of shared library S bacterial barcodes between tissues in IgG animals as compared with both naive and αCD4-treated animals, further supporting that reinfection reduced bacterial dissemination in a $CD4^+$ T cell-dependent manner (Figure 2H). As in our original reinfection study,[8] library S *Mtb* could be found in pre-existing granulomas (i.e., those from the primary infection that were cleared of *Mtb* with antibiotics). This was variable across animals but suggested that *Mtb* could traffic to sites of old disease, likely due to inflammatory signals present in healed lesions[26] (Figure S2J).

These data were most consistent with a model in which previous drug treated infection led to an immune environment that

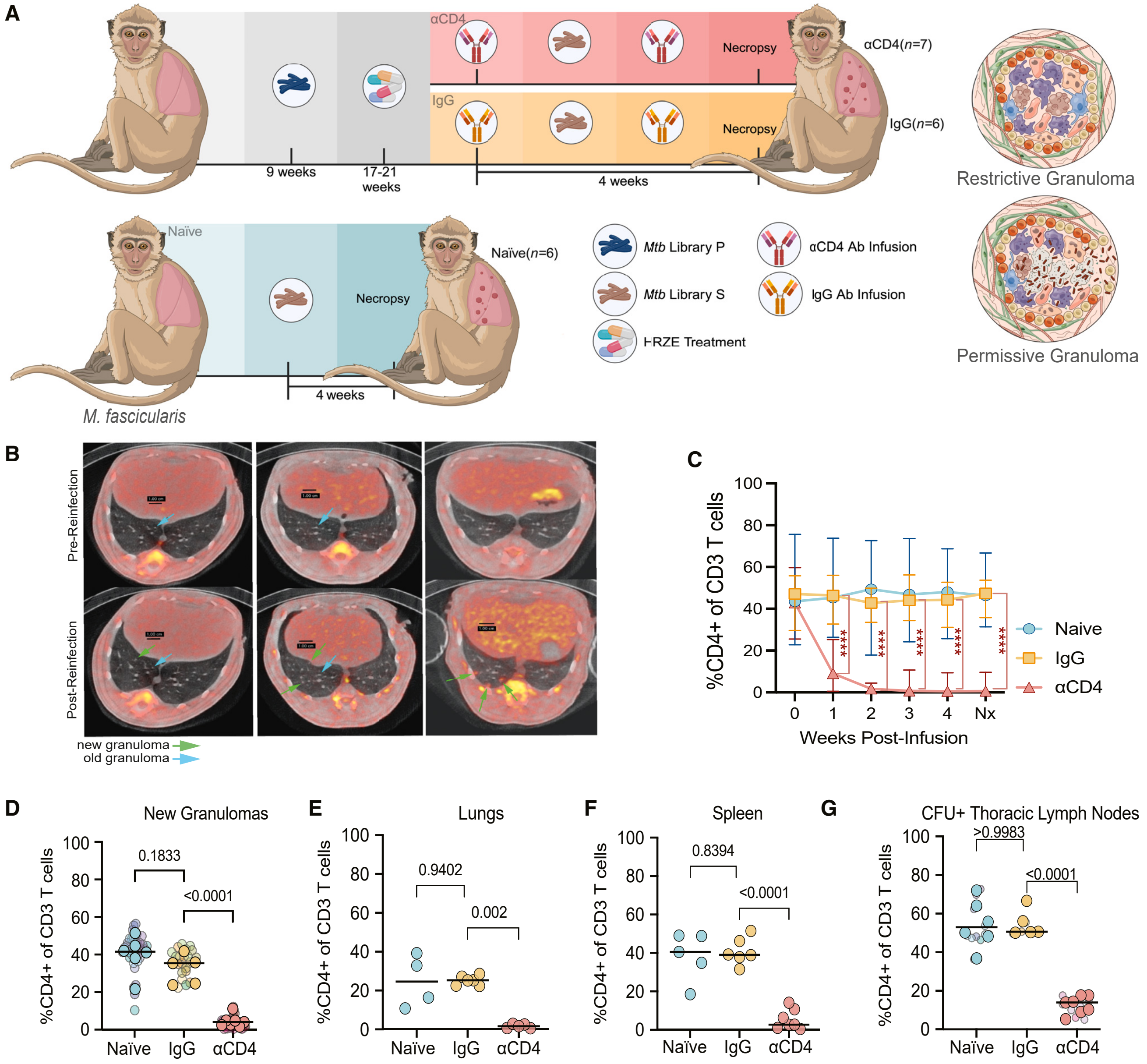

Figure 1. Anti-CD4 antibody infusion depleted CD4$^+$ T cells across anatomic compartments in cynomolgus macaques

(A) Experimental design (BioRender.com).

(B) PET-CT scan of representative NHPs pre- and post-reinfection. Old granulomas: blue arrows; new granulomas: green arrows. Left: IgG; middle: αCD4; right: naive.

(C) Fraction of CD3 that were CD4$^+$ post-antibody infusion in blood (number of animals: naive n = 6; IgG n = 6; αCD4 n = 7). Median and range shown (****p < 0.0001; mixed-effects model with Dunnett's multiple comparisons test).

(D) CD3$^+$, CD4$^+$ cells in new library S granulomas (number of granulomas: naive n = 37; IgG n = 21; aCD4 n = 38; number of animals: naive n = 6; IgG n = 5; αCD4 n = 7).

(E) CD3$^+$, CD4$^+$ cells in uninvolved lung tissue from *Mtb* infected macaques (number of animals: naive n = 4, IgG n = 6; αCD4 n = 5).

(F) CD3$^+$, CD4$^+$ cells in spleen (number of animals: naive n = 5, IgG n = 6, αCD4 n = 7).

(G) CD3$^+$, CD4$^+$ cells from CFU$^+$ LNs (number of animals: naive n = 6, IgG n = 5; αCD4 n = 7; number of LNs: naive n = 14; IgG n = 6; αCD4 n = 11).

(D–G) Transparent smaller dots represent granulomas, colored by animal. Larger dots represent mean per animal, and lines represent medians. One-way ANOVA with Dunnett's multiple comparisons test.

See also Figure S1 and Table S1. Experiment was performed once.

CellPress
OPEN ACCESS

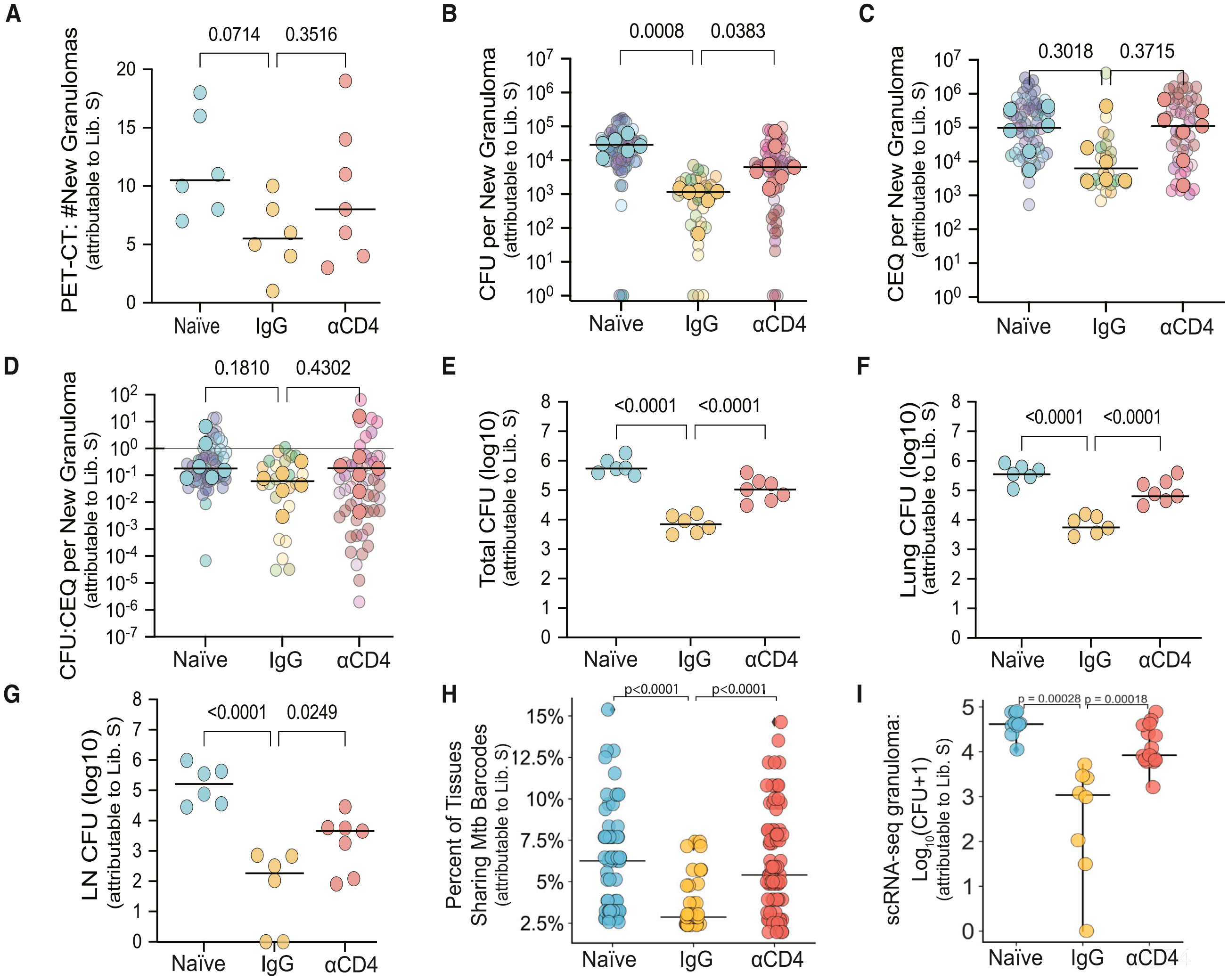

Figure 2. Reinfection with *Mtb* reduced granuloma formation, as well as bacterial burden and dissemination in a CD4$^+$ T cell-dependent manner

(A) Number of new granulomas identified using PET-CT following infection with *Mtb* library S. Lines represent medians. One-way ANOVA with Dunnett's multiple comparison test, adjusted *p* values reported.

(B) Median number of viable *Mtb* colony-forming units (CFUs) per library S granuloma (Kruskal-Wallis with Dunn's multiple correction). Solid dots represent the median CFU/granuloma per animal; lines represent medians. Transparent dots represent the median CFU of individual granulomas. Number of granulomas: naive *n* = 70, IgG *n* = 34; αCD4 *n* = 65.

(C) Median number of chromosomal equivalents (CEQs) per macaque (Kruskal-Wallis with Dunn's multiple correction). Solid dots represent the median CFU per animal; lines represent medians. Transparent dots represent the median CFU of individual granulomas.

(D) CFU:CEQ ratio, a proxy for bactericidal activity (Kruskal-Wallis with Dunn's multiple correction). Solid dots represent the median CFU per animal; lines represent medians. Transparent dots represent the median CFU of individual granulomas.

(C and D) Number of granulomas: naive *n* = 59, IgG *n* = 26; αCD4 *n* = 51.

(E) Total CFU from granulomas, uninvolved lung tissue, and thoracic lymph nodes.

(F) Lung CFU from granulomas and uninvolved lung tissue.

(G) Thoracic lymph node CFU.

(E–G) Lines represent medians. One-way ANOVA with Dunnett's multiple comparisons test.

(A–G) Number of macaques: naive *n* = 6; IgG *n* = 6; anti-CD4 *n* = 7.

(H) Percent of all sampled tissues that shared library S barcodes (Kruskal-Wallis with Dunn's multiple comparison test). Data points in each treatment arm reflect number of distinct barcodes (naive, *n* = 77; IgG, *n* = 40; anti-CD4, *n* = 87) identified by sequencing; these barcodes were aggregated across independent animals (naive, *n* = 6; IgG, *n* = 6; anti-CD4, *n* = 7).

(I) Individual granuloma *Mtb* CFU for single granulomas subjected to Seq-Well S^3 scRNA-seq (Mann-Whitney U test). Naive *n* = 10, IgG *n* = 8, αCD4 *n* = 15 granulomas from 2 naive, 2 IgG, and 3 αCD4 NHPs. Experiment was performed once.

See also Figure S2 and Table S1.

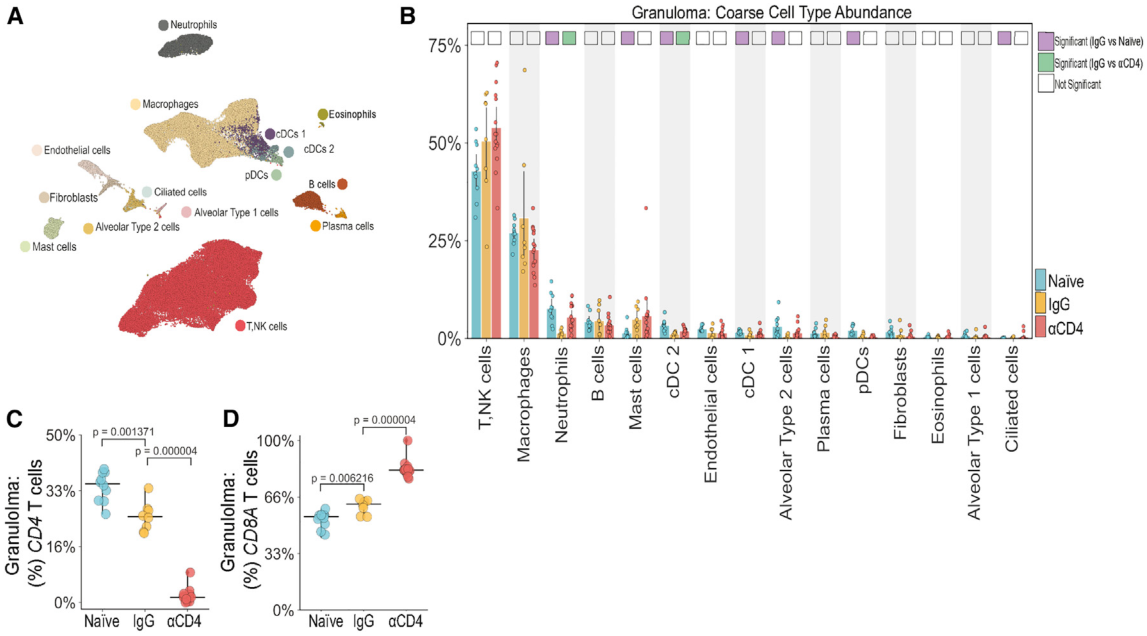

Figure 3. *Mtb* reinfection promoted cellular remodeling of the TB granuloma microenvironment

(A) UMAP embedding of Seq-Well S^3-derived granuloma transcriptomes colored by coarse cell type.

(B) Coarse cell type frequencies colored by experimental group. Differentially abundant IgG vs. naive (purple) and IgG vs. αCD4 (green) marked with colored square. Cell types were differentially abundant if significant using two of three methods: Mann-Whitney U test; scCODA,[29] and Fisher's exact test.

(C) Fraction of granuloma T, NK cells expressing *CD4* from Seq-Well S^3-derived transcriptomes (Mann-Whitney U test).

(D) Fraction of granuloma T, NK cells expressing *CD8A* from Seq-Well S^3-derived transcriptomes (Mann-Whitney U test). Individual dots in (B)–(D) represent single granulomas. Naive *n* = 10, IgG *n* = 8, αCD4 *n* = 15 granulomas from 2 naive, 2 IgG, and 3αCD4 NHPs. Experiment was performed once.

See also Figure S3 and Tables S2, S3, and S4.

restricted bacterial growth in a CD4$^+$ T cell-dependent fashion but did not prevent establishment of infection or drive substantially increased bacterial killing.

Cellular remodeling of the TB granuloma microenvironment occurred following *Mtb* reinfection

To define the cellular features associated with protection against reinfection and the effects of CD4$^+$ T cell depletion, we performed Seq-Well S^3-based[27] massively parallel scRNA-seq on granulomas isolated from the three experimental groups (Figures 2I and 3A). We analyzed 33 granulomas that were confirmed to arise from *Mtb* library S (naive = 10, IgG = 8, αCD4 = 15) from 7 cynomolgus macaques (naive = 2, IgG = 2, αCD4 = 3), yielding a total of 88,360 high-quality transcriptomes. We annotated 15 clusters corresponding to distinct immune and non-immune cell types based on known marker genes and reference signatures[28] (Figures 3A and S3A). While cellular frequencies varied among individual granulomas and experimental groups, each cluster was represented by multiple samples (Figure S3B).

We sought to identify whether there were significant changes in cell type frequencies across granulomas. We implemented multivariate (scCODA[29]), univariate (Mann-Whitney U test), and nonparametric (Fisher's exact test) tests to account for perturbation, cell type codependences, and low sample size and considered a cell type differentially abundant if significant by at least two tests.[30] We first assessed the global T cell composition of lesions from the three groups and observed a trend toward higher overall T, natural killer (NK) cell frequencies among IgG granulomas relative to naive (Figure 3B). There was a trend toward increased T, NK cell frequencies among all reinfection granulomas, so we sought to identify whether prior infection promoted CD4$^+$ or CD8$^+$ T cell recruitment to the granuloma. The total fraction of *CD4* and *CD8* T cells among *CD3D* expressors was significantly lower and higher in IgG granulomas relative to naive granulomas, respectively (Figures 3C and 3D). The former is in line with flow cytometric data demonstrating a trend toward lower frequencies of CD4$^+$ T cells in IgG granulomas relative to naive granulomas (Figure 1D). There was no difference in the frequency of total T, NK cells between IgG and αCD4 granulomas, but the latter had significantly fewer *CD4* T cells and more *CD8* T cells (Figures 3C and 3D).

IgG granulomas had lower frequencies of neutrophils relative to naive granulomas, and depletion of CD4$^+$ T cells led to increased frequencies of neutrophils. IgG granulomas also had lower frequencies of cDC2s relative to both naive and αCD4 granulomas. Mast cells were more frequent in IgG lesions compared with naive granulomas, but this was not altered by CD4$^+$ T cell depletion. IgG granulomas also had lower frequencies of cDC1s, alveolar type 2 cells, ciliated cells, and pDCs as compared with naive granulomas. Altogether, these data demonstrated a shift in granuloma cellularity following *Mtb* reinfection.

CD4$^+$ T cells regulated immune tone in reinfection granulomas

To better understand how reinfection and CD4$^+$ T cell depletion modulated T, NK cell phenotypes, we classified all T, NK cells

CellPress
OPEN ACCESS

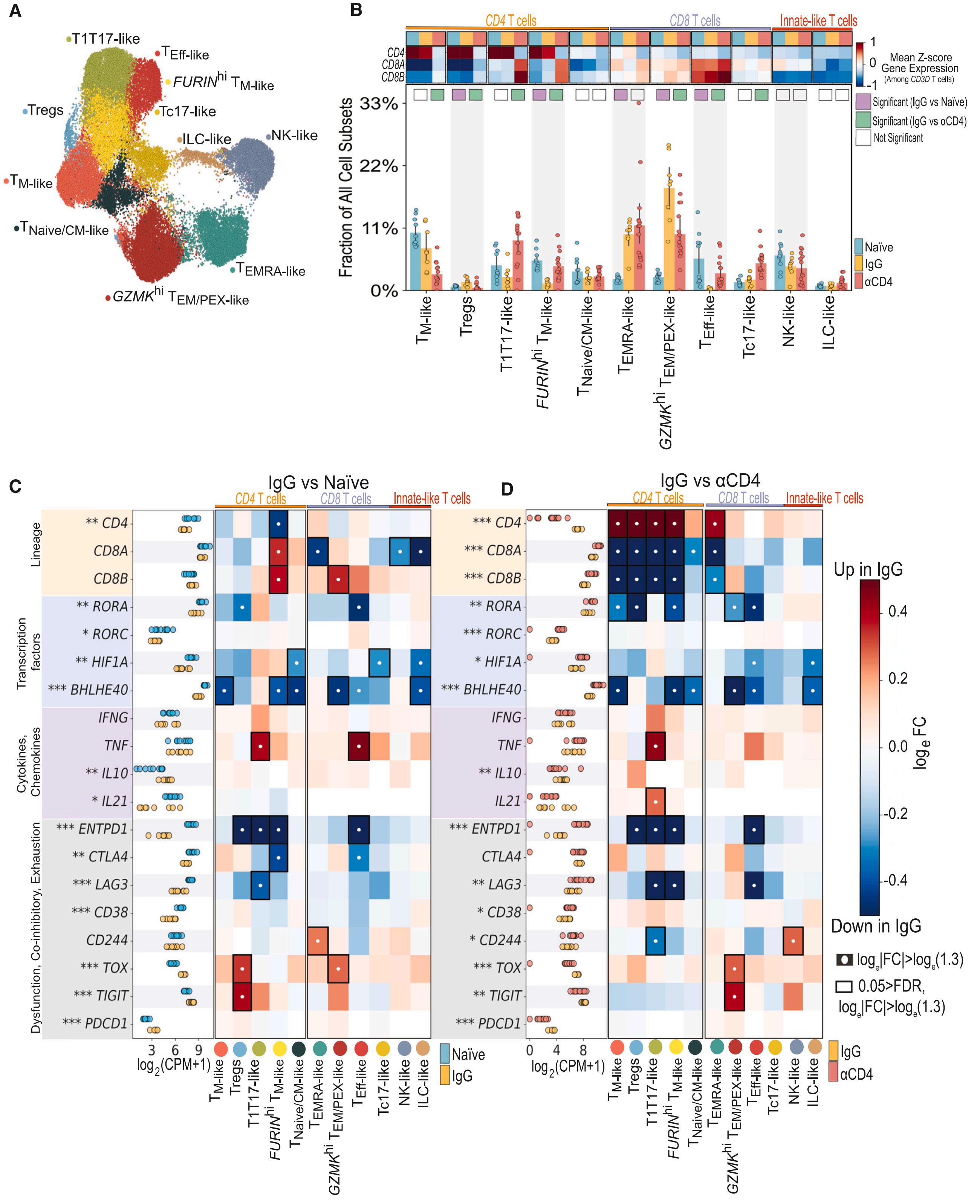

Figure 4. CD4[+] T cells regulated T cell cellularity, cytokine flux, and immune tone in the TB granulomas following *Mtb* reinfection

(A) UMAP embedding depicting T, NK cell subpopulations identified by sub-clustering.

(B) Heatmap depicting gene expression (mean *Z* score) of T cell lineage markers *CD4*, *CD8A*, and *CD8B*. Columns represent gene expression in individual NHP groups—naive (light blue), IgG (yellow), and αCD4 (red). Bar plot of T, NK subpopulation frequencies among all granuloma cellular subpopulations colored by

(legend continued on next page)

into 11 major subpopulations based on gene signatures from external single-cell datasets[31–33] (Figures 4A and S3C). The proportions of several T, NK cell subsets differed among reinfection (IgG) granulomas, particularly the *CD8* enriched (*GZMK*^{hi} $T_{EM/PEX\text{-}like}$)[34,35] and *CD8*, *CD4* co-expressing ($T_{EMRA\text{-}like}$)[36,37] subsets. We found significant enrichment of immuno-modulatory and -regulatory *CD8 GZMK*^{hi} $T_{EM/PEX\text{-}like}$ cells in IgG granulomas relative to naive ones; CD4 depletion significantly impaired recruitment (or retention) of these cells, suggesting CD4 dependence even after immune priming (Figure 4B). Frequencies of Tregs, characterized by *CD4* expression and immuno-modulatory molecules, were elevated among IgG granulomas compared with naive lesions.

Of the observed *CD4*-expressing T, NK cell subsets, only *FURIN*^{hi} T memory-like (*FURIN*^{hi} $T_{M\text{-}like}$) cells were fractionally more abundant in naive vs. IgG lesions (Figure 4B). However, type 1, type 17 (T1T17-like) cells—a T cell subset previously implicated in *Mtb* control[18]—were not differentially abundant between IgG and naive granulomas. In the absence of CD4^{+} T cells, however, we found significant T1T17-like enrichment. αCD4 T1T17-like cells expressed *CD8*, whereas IgG and naive T1T17-like cells were enriched for *CD4*, potentially suggesting that in the absence of CD4^{+} T cells, CD8^{+} T1T17-like cells coopted similar gene programming in an effort to compensate for loss of CD4^{+} T cells.

We next investigated how infection status and immune perturbation altered global T, NK cell responses, as well as those of each cell subpopulation, by performing pairwise (IgG vs. naive; IgG vs. αCD4) differential gene expression (DGE) for pseudobulked T, NK cells (i.e., all T, NK subsets aggregated) and within each T cell subpopulations. Pairwise analyses among the 11 identified T, NK cell subsets revealed a total of 1,542 differential expression (DE) genes (773 upregulated, 759 downregulated) in IgG vs. naive lesions and 1,263 DE genes (783 upregulated, 480 downregulated) in IgG vs. αCD4 granuloma, demonstrating significant shifts in T cell circuitry in immunologically primed (IgG) animals relative to naive or CD4-depleted animals (Figures 4C, 4D, and S3D–S3F).

To examine the potential functional significance of these DE genes, we queried cytokines canonically associated with protective anti-*Mtb* responses, including *TNF*, *IFNG*, and the pleiotropic cytokine *IL10*, while excluding *IL17* due to its low expression (Figure 4C). There were no significant differences in *TNF* or *IFNG* expression among IgG vs. naive granulomas for global (pseudobulk) or T, NK subset comparisons—with the exception of T1T17-like and $T_{eff\text{-}like}$ cells. We observed significant induction of *IL10* expression in IgG compared with naive granulomas globally (Figure 4C). However, there was no one T, NK subset that was significantly enriched for *IL10* expression among IgG lesions; rather, *IL10* was expressed across several subsets. IgG granulomas were also characterized by greater global expression of immunoregulatory genes (*TIGIT*, *TOX*), primarily associated with Treg and *GZMK*^{hi} $T_{EM/PEX\text{-}like}$ subsets. Relative to naive granulomas, IgG granulomas had lower expression of cytotoxic effector (*GZMA*, *GZMB*), hypoxia-induced factors (*HIF1A*, *BHLHE40*, *ENTPD1*),[40] T1T17-like transcription factors (TFs) (*RORA*, *RORC*), and interferon (IFN)-stimulated genes (*ISG15*, *MX1*) across several T, NK subsets (Figures 4C and S3D). In agreement with these analyses, pathway responsive genes (PROGENy)[41] analyses revealed that IgG (vs. naive) T, NK cells upregulate VEGF and p53 pathways, suggesting VEGF may contribute to the heightened expression of several co-inhibitory checkpoints (e.g., *PDCD1*, *CTLA4*).[42]

In the setting of CD4^{+} T cell depletion, there was a global reduction in *IL10*, *PDCD1*, *TIGIT*, and *TOX* gene expression (Figure 4D). Furthermore, in the absence of CD4^{+} T cells, there was a subset-specific reduction in costimulatory (*CD28*, *CD40LG*) and negative regulators (*CD5*, *CD6*) of T cell activation, as well as altered induction of core regulatory pathways (Figures S3E and S3H). In αCD4 (vs. IgG) granulomas, there was higher expression of T1T17-associated TFs, the PD-1-repressor *SATB1*,[43] and *BHLHE40*,[44] a putative negative regulator of *IL10* expression and hypoxia-induced factor (Figures 4D and S3E). Collectively, these changes suggested CD8^{+} T cell reprogramming following *Mtb* reinfection and that acquisition of aspects of these terminally differentiated and immunoregulatory CD8^{+} T, NK cell gene programs were CD4 dependent.

Monocyte-derived transcriptomes featured attenuated type 1 immunity in *Mtb* reinfection granulomas

Considering the established paradigm where CD4^{+} T cells orchestrate pro-inflammatory myeloid cell responses primarily through IFN-γ and TNF-mediated pathways, we explored whether the observed increase in immunoregulatory T cell phenotypes among IgG granulomas was associated with altered myeloid cellularity and transcriptional programming in reinfection. Monocyte-derived cells partitioned into 6 subpopulations and exhibited varying degrees of expansion or contraction in naive, IgG, and αCD4 granulomas (Figures 5A, 5B, and S4A). Notably, IgG lesions featured a significant decrease in "M1"- and interstitial-like *CXCL9*^{hi}, *IDO1*^{hi} macrophages (MΦs) and elevated frequencies of *FABP4*^{hi}, *MCEMP1*^{hi} Alveolar MΦs, relative to naive and αCD4 granulomas. Subset-specific gene set enrichment analyses revealed *CXCL9*^{hi} *IDO1*^{hi} MΦs engaged chemokine induction pathways, whereas *FABP4*^{hi}, *MCEMP1*^{hi} Alveolar MΦs upregulated lipid-metabolizing transcription programs—a prominent feature of "M2-like" MΦs and *Mtb*-permissive phagocytes[45] (Figure S4B).

experimental group. Differentially abundant IgG vs. naive (purple) and IgG vs. αCD4 (green) marked with colored square. Cell types are differentially abundant if significant using two of three methods: Mann-Whitney U test; scCODA, and Fisher's exact test.

(C and D) T, NK cell pseudobulk $\log_2$(CPM + 1) for naive (light blue), IgG (yellow), and αCD4 (red) NHP granulomas (***$p < 0.001$,**$p < 0.01$, *$p < 0.05$; Wilcoxon rank-sum test). Heatmap depicting $\log_e$FC (calculated using MAST[38,39]) of lineage markers, cytolytic molecules, select transcription factors, immunoregulatory molecules, and chemokines, and cytokines (rows) for each cell type (columns) in NHP granulomas IgG vs. naive (C) or IgG vs. αCD4 lesions (D). White circles indicate $\log_e|FC| > \log_e(1.3)$, relative to naive or αCD4 granulomas. Black rectangles indicate 0.05 > false discovery rate (FDR) and $\log_e|FC| > \log_e(1.3)$, relative to naive or αCD4 granulomas. Individual dots in (B)–(D) represent single granuloma. Naive n = 10, IgG n = 8, αCD4 n = 15 granulomas from 2 naive, 2 IgG, and 3 αCD4 NHPs.

See also Figure S3 and Tables S5, S6, S7, and S8.

CellPress
OPEN ACCESS

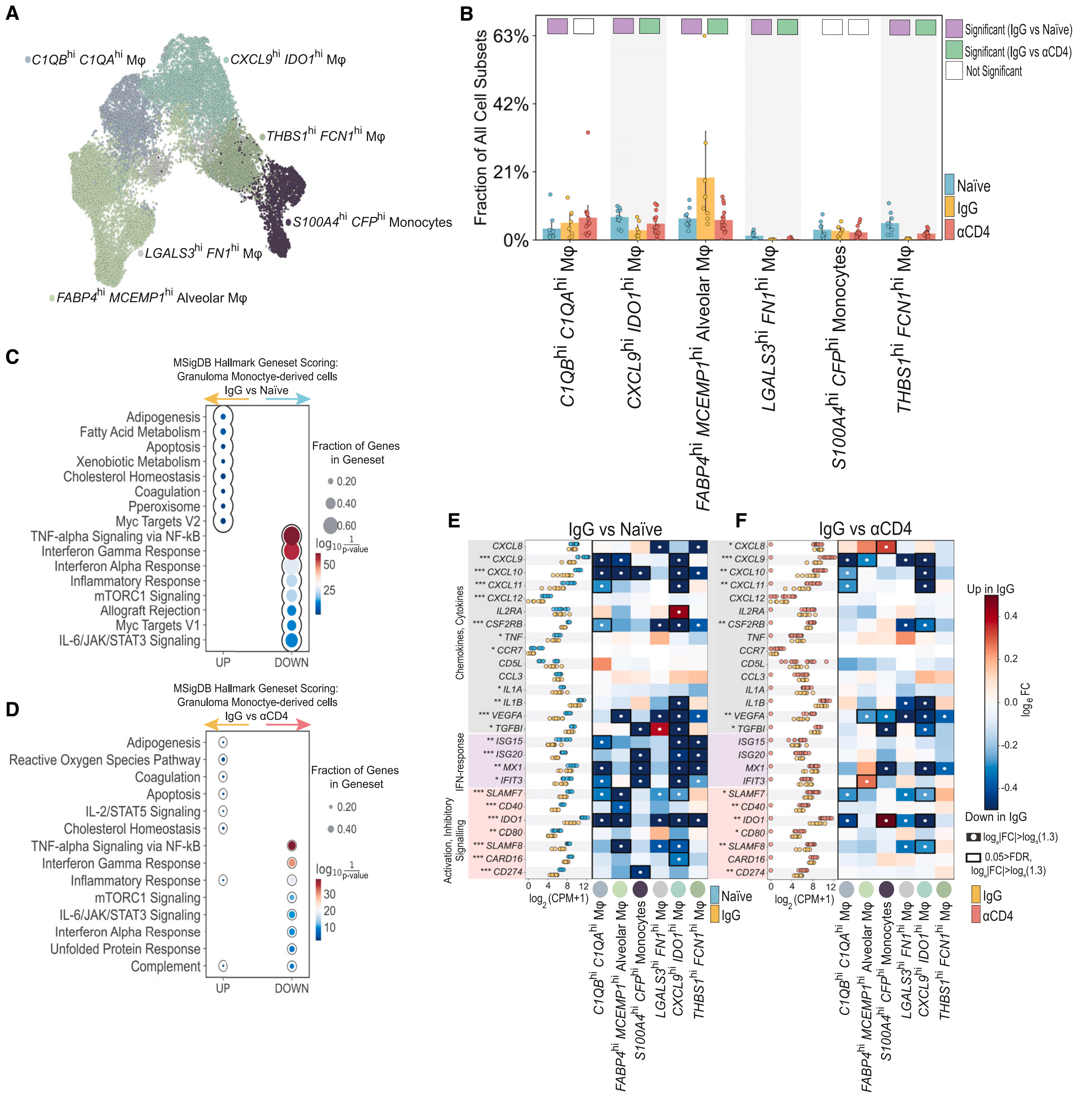

Figure 5. Monocyte-derived transcriptomes featured attenuated type 1 immunity in *Mtb* reinfection granulomas

(A) UMAP embedding depicting monocyte-derived cells identified by sub-clustering. UMAP embeddings depicting monocyte-derived cell subpopulation densities, split by NHP cohort.

(B) Bar plot of monocyte-derived progenitor frequencies, among all granuloma cell subpopulations, colored by experimental group. Individual dots represent single granulomas. Differentially abundant IgG vs. naive (purple) and IgG vs. αCD4 (green) marked with colored square. Cell types are differentially abundant if significant using two of three methods: Mann-Whitney U test; scCODA, and Fisher's exact test.

(C and D) Enriched pathways from identified using differentially expressed genes (Mann-Whitney U test (Wilcoxon rank-sum) (p value < 0.05)) from naive, IgG, and αCD4 sizes. Circle size represents the number of genes in Hallmark Geneset, and color (red-blue) represents geneset enrichment score. Genesets that are "up" (x axis) are enriched among IgG granulomas, whereas "down" genesets are enriched among naive (C) and αCD4 (D) granulomas, respectively.

(E and F) Monocyte-derived pseudobulk $\log_2$(CPM + 1) for naive (light blue), IgG (yellow), and αCD4 (red) NHP granulomas (***p < 0.001, **p < 0.01, *p < 0.05; Wilcoxon rank-sum test). Heatmap depicting $\log_e$FC of select transcription factors, immunoregulatory molecules, chemokines, and cytokines (rows) for each cell

(legend continued on next page)

To identify global changes in myeloid gene programming following *Mtb* reinfection, we scored all monocyte-derived cells against Hallmark gene sets (Figures 5C and 5D) for gene set enrichment scoring. Our analyses demonstrated a global reduction in inflammatory responses, specifically IFN-α-, interleukin (IL)-6/JAK/STAT3, and IFN-γ-responses, as well as significant enrichment of adipogenesis, fatty acid metabolism, Myc targets, and DNA repair signatures among monocyte-derived subsets in IgG relative to naive granulomas. By contrast, in αCD4 granulomas, the macrophages had increased IFN-α, IL-6/JAK/STAT3, and IFN-γ inflammatory responses relative to IgG granulomas.

Pairwise DGE analyses among monocyte-derived subpopulations revealed a total of 2,210 DE genes (1,236 upregulated, 974 downregulated) in IgG vs. naive lesions and 1,234 DE genes (777 upregulated, 457 downregulated) in IgG vs. αCD4 granuloma (Figure S4C). Myeloid cells from naive granulomas featured both global and subpopulation-specific increases in expression of IFN-stimulated genes (*ISG15*, *ISG20*), pro-inflammatory mediators (*IL1A*, *ILB*), chemokines and cytokines including the CXCR3 ligands (*CXCL9*, *CXLC10*, *CXCL11*), fibrosis-related genes (*VEGFA*, *TGFB1*), and immunoregulatory molecules (*IDO1*, *CD274* (PD-L1), *IL10*) relative to macrophages in IgG granulomas (Figures 5E, 5F, and S4D). In the absence of $CD4^+$ T cells, a pseudobulk analysis indicated increased type 1 (e.g., *CXCL9–11*) immune signaling. A subpopulation-specific DE analysis of αCD4 (vs. IgG) revealed various monocyte-derived subsets upregulated *CXCR3* ligands, *IDO1*, and *CD274*, but not *IL10*, in αCD4 granulomas (Figures 5F and S4E).

Overall, naive granulomas showed enhanced type 1 immune signaling compared with IgG granulomas. The reversion of granulomas toward a naive-like state with $CD4^+$ T cell depletion indicated a regulatory role for $CD4^+$ T cells over the myeloid-driven inflammatory response during *Mtb* reinfection.

Prior infection influenced neutrophil response and dampened induction of a type 1 IFN gene module upon reinfection

Neutrophils play a crucial role as frontline defenders against microbial infections and are quickly recruited to sites of inflammation upon *Mtb* infection. However, their role in TB disease remains enigmatic, as they promote both *Mtb* control and pathology.[46] To evaluate how prior infection and $CD4^+$ T cells modulated neutrophil recruitment and phenotype upon reinfection, we quantified differences in cellular frequencies and gene expression following sub-clustering. Our analysis identified two neutrophil subpopulations: *ICAM1*^{hi}, *NBN*^{hi} neutrophils and *SORL1*^{hi}, *CFD*^{hi} neutrophils[47] (Figures 6A and S4F). Both neutrophil subpopulations were significantly underrepresented among IgG lesions compared with naive and αCD4 granulomas, suggesting that bacterial burden and/or $CD4^+$ T cells regulate neutrophilic response and infiltration (Figure 6B).

To uncover potential differences in neutrophil transcriptional programming, we performed pairwise pseudobulk DGE analysis and pairwise DGE analyses across conditions between these neutrophil subsets (Figure S4G). Comparisons of *ICAM1*^{hi}, *NBN*^{hi} to *SORL1*^{hi}, *CFD*^{hi} transcriptomes revealed *ICAM1*^{hi}, *NBN*^{hi} neutrophils upregulate type 1 immune chemokines (*CXCL10*) and cytokines (*CCL3*, *IL1A*), whereas *SORL1*^{hi}, *CFD*^{hi} neutrophils upregulated molecules implicated in neutrophil trafficking (*CXCR1*, *CXCR2*) and netosis (*MGAM*, *MMP25*)[48–51] (Figure 6C). Pairwise DE analyses (naive vs. IgG and αCD4 vs. IgG) revealed few differences in gene programming among *ICAM1*^{hi}, *NBN*^{hi} transcriptomes; however, *SORL1*^{hi}, *CFD*^{hi} neutrophils had substantially altered transcriptomes among IgG lesions relative to naive. Neutrophils from naive granulomas featured robust expression of inflammatory response genes, type 1 immune chemokines,[52] and cytokines. CD4-depleted lesions, meanwhile, exhibited similar "naive-like" neutrophil gene programming compared with IgG lesions (Figures 6D, 6E, and S4G). We scored neutrophils against an IFN-inducible neutrophil gene signature[53] shown to be upregulated in humans with active TB (Figure 6F). In line with our DE analyses, IgG neutrophils featured significant blunting of IFN-inducible genes.

Collectively, our data delineated the diversity among granuloma-localized neutrophils and demonstrated a significant reduction in neutrophilic responses among IgG compared with naive or αCD4 granulomas, implying a regulatory role for $CD4^+$ T cells on neutrophil-driven immunity and TB disease progression. Furthermore, these data supported the model that increased neutrophilic infiltration may contribute to formation of *Mtb*-permissive niches, thus contributing to elevated bacillary loads among naive and αCD4 lesions.[54,55]

Differential cell-cell interactions occurred in immunologically primed granulomas

To assess how the aforementioned factors act together to modulate host immunity, we investigated differential cell-cell[56] interaction networks among naive, IgG, and αCD4 granulomas. First, we identified differences among coarse-grain cell-cell interactions occurring in primary infection granulomas (naive) vs. those formed in a primed immune environment (IgG). Relative to IgG lesions, naive cell-cell interaction networks were dominated by signaling from neutrophils, macrophages, and non-immune cells (endothelial cells and fibroblasts) and enriched for type 1 immune (*CXCL9–11*, *IL6 IL1B*, *TNF*) and type 1 IFN (*IFNB1*, *IFNA1*, *IFNA2*, *IFNA16*)[57] signaling—the latter implicated in TB pathogenesis and previously demonstrated to contribute to neutrophil extracellular trap formation and subsequent *Mtb* proliferation (Figures 7A, 7B, and S5A–S5E).

To identify subpopulation-specific drivers of granuloma immune tone and cytokine flux, we quantified differential cell-cell interactions among all immune cell subpopulations (Figures S5F and S5G). This analysis identified several monocyte-derived subpopulations (*S100A4*^{hi}, *CFP*^{hi} monocytes, *FABP4*^{hi},

type (columns) in NHP granulomas IgG vs. naive (E) or IgG vs. αCD4 lesions (F). White circles indicate $\log_e|FC| > \log_e(1.3)$, relative to naive or αCD4 granulomas. Black rectangles indicate 0.05 > FDR and $\log_e|FC| > \log_e(1.3)$, relative to naive or αCD4 granulomas.
Individual dots in (B), (E), and (F) represent single granulomas. Naive $n = 10$, IgG $n = 8$, αCD4 $n = 15$ granulomas from 2 naive, 2 IgG, and 3 αCD4 NHPs.
See also Figure S4 and Tables S5, S6, S7, and S8.

CellPress
OPEN ACCESS

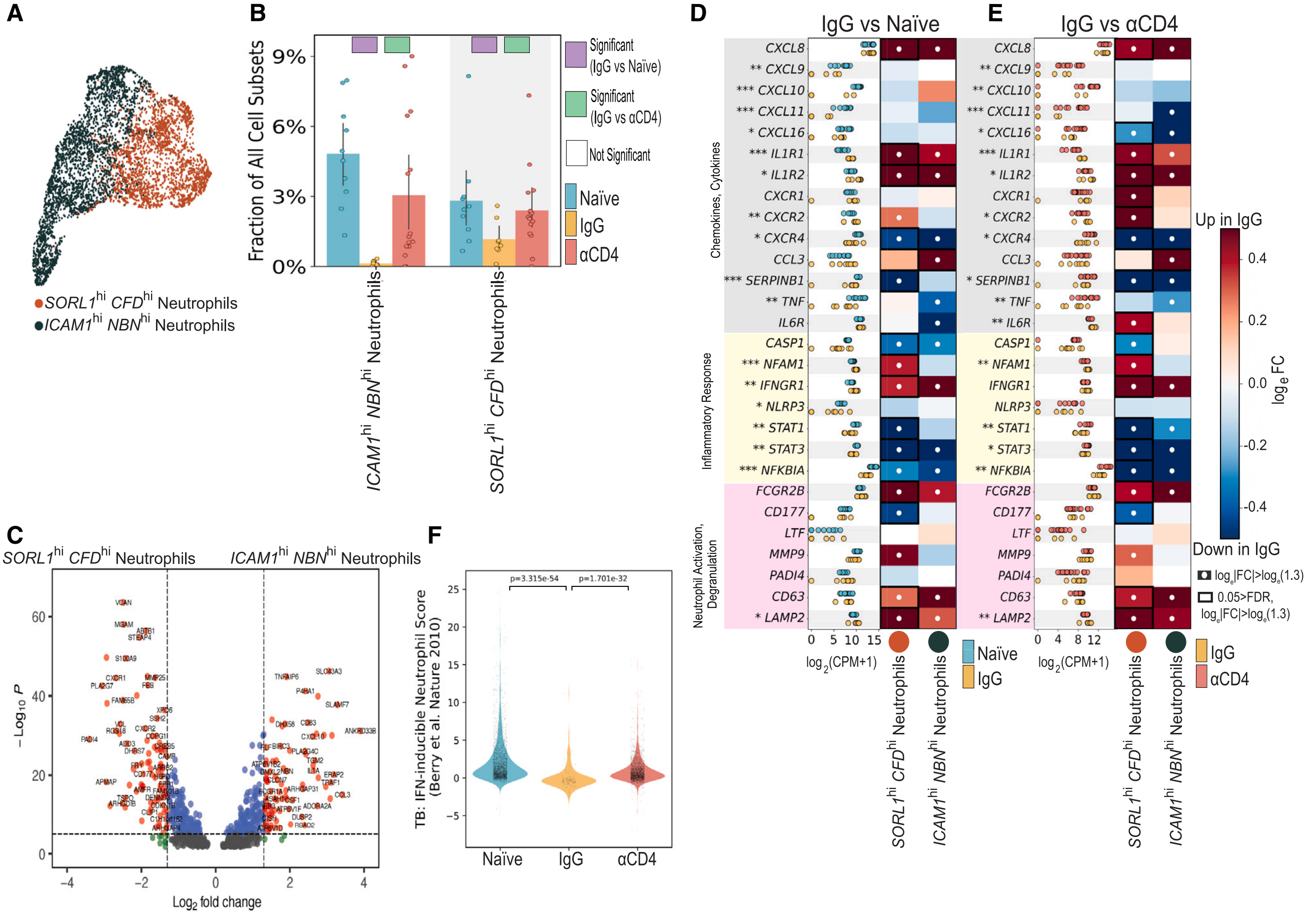

Figure 6. Prior infection influenced neutrophil response and dampened induction of a TB susceptibility type 1 interferon gene module

(A) UMAP embedding depicting neutrophil cell subpopulations identified by sub-clustering.

(B) Bar plot of neutrophil subset frequencies, among all granuloma cell subpopulations, colored by experimental group. Individual dots represent single granulomas. Differentially abundant IgG vs. naive (purple) and IgG vs. αCD4 (green) marked with colored square. Cell types are differentially abundant if significant using two of three methods: Mann-Whitney U test; scCODA, and Fisher's exact test.

(C) Volcano plot depicting pseudobulk differential gene expression (DESeq2) *ICAM1*^{hi}, *NBN*^{hi} vs. *SORL1*^{hi}, *CFD*^{hi} neutrophils (for all NHP experimental groups). Volcano plot x axis indicates the $\log_2$FC, and y axis indicates the $-\log_{10}(p$ value).

(D and E) Neutrophil pseudobulk $\log_2$(CPM + 1) for naive (light blue), IgG (yellow), and αCD4 (red) NHP granulomas (***$p < 0.001$,**$p < 0.01$, *$p < 0.05$; Wilcoxon rank-sum test). Heatmap depicting $\log_e$FC of select transcription factors, immunoregulatory molecules, chemokines, and cytokines (rows) for each cell type (columns) in NHP granulomas IgG vs. naive (D) or IgG vs. αCD4 lesions (E). White circles indicate $\log_e|FC| > \log_e(1.3)$, relative to naive or αCD4 granuloma. Black rectangles indicate $0.05 >$ FDR and $\log_e|FC| > \log_e(1.3)$, relative to naive or αCD4 granulomas.

(F) Violin plots of IFN-inducible neutrophil module scores by NHP group. Mann-Whitney U test. Individual dots in (B), (D), and (E) represent single granulomas. Naive $n = 10$, IgG $n = 8$, αCD4 $n = 15$ granulomas from 2 naive, 2 IgG, and 3 αCD4 NHPs.

See also Figure S4 and Tables S5, S6, S7, and S8.

MCEMP^{hi} alveolar MΦ, *CXCL9*^{hi} *IDO1*^{hi} MΦ) sending the type 1 immune molecules *CXCL9–10*, and neutrophils (*ICAM1*^{hi}, *NBN*^{hi} neutrophils, *SORL1*^{hi}, *CFD*^{hi} neutrophils) sending *TNF*, *CSF1*, and *CXCL10*, which targeted both innate and adaptive (e.g., *CXCR3* T1T17-like) subpopulations (Figure S5F). In addition to upregulated type 1 immune factors, our subpopulation-specific cell-cell interaction analyses identified an increased diversity of *IL10* "senders" among naive (vs. IgG) granulomas with prominent monocyte-derived (*THBS1*^{hi}, *FCN1*^{hi} MΦ; *LGALS3*^{hi}, *FN1*^{hi} MΦ; *FABP4*^{hi}, *MCEMP*^{hi} alveolar MΦ; *CXCL9*^{hi} *IDO1*^{hi} MΦ) *IL10* senders which targeted *ICAM1*^{hi}, *NBN*^{hi} neutrophils, and several monocyte-derived subpopulations as "receivers" (Figures 7C and S5F–S5I). Collectively, these data suggested type 1 immune signaling networks promoted recruitment of adaptive immune cells and induced a pro-inflammatory immune response to mount an early anti-microbial response, whereas *IL10* monocyte-derived subpopulations might mitigate this inflammatory response via self-reinforcing innate-innate immune cell circuits.

In contrast to naive lesions, our analysis of coarse-grain cell types in IgG granulomas revealed T, NK cells as the dominant receiver cell type, and macrophages as the most prolific senders, communicating not only with T, NK cells but also with mast cells (Figures 7D, 7E, S5B, S5D, and S5E). Outgoing macrophage-derived signaling was dominated by negative regulators of inflammatory response (*CD9*, *CD52*, *CDH1*), wound

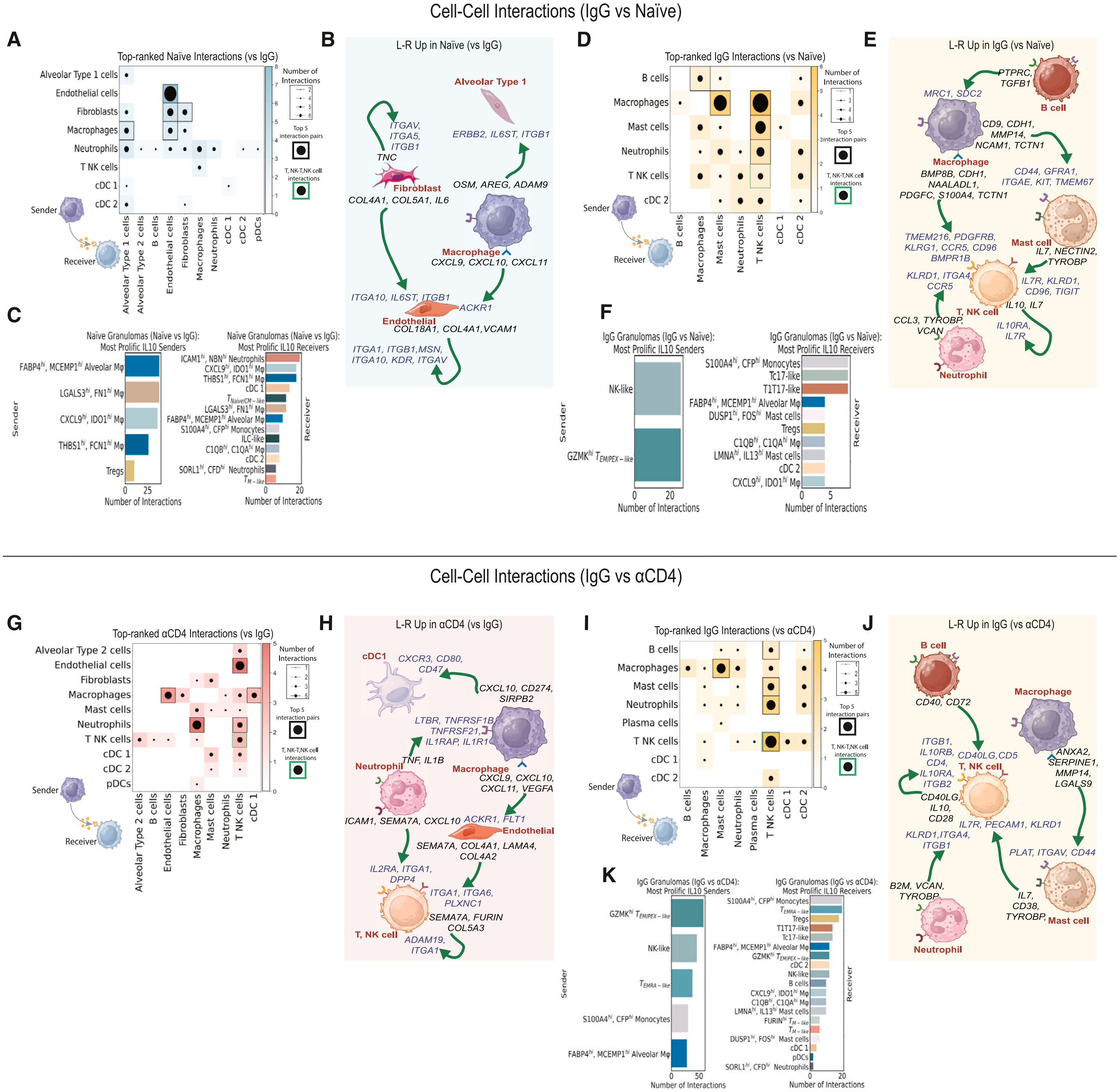

Figure 7. Differential cell-cell interactions occurred in immunologically primed granulomas

(A) Heatmap depiction of differential (naive vs. IgG) cell-cell interaction pairs among coarse cell types. Columns represent cell-cell interactions from the top-prioritized links—"sender" ligands and receptors differential L-R pairs specific to IgG or naive granulomas. Heatmap and dot size represent L-R interactions from the 50 top-prioritized links. Black rectangles indicate top 5 interactions, based on number of interactions between two cell types per NHP group. Green rectangles depict putative T, NK-T, NK interactions.

(B) Cartoon depiction of (A) with differential L-R in naive granulomas.

(C) Bar plot depiction of differential cell-cell interactions among naive granulomas. Left: *IL10* sender cellular subpopulations; right: *IL10RA/RB* cell sub-populations. Receptor-ligand and inferred interaction pairs are derived from the top 200 top-prioritized linkages.

(D) Similar heatmap to that of (A), highlighting linkages specific to IgG (vs. naive) granulomas.

(E) Schematic representation of the differential L-R pairs in IgG granulomas from (D).

(F) Bar plot representation of differential *IL10-IL10RA/RB* interactions among IgG lesions, similar to that of (C).

(G) Heatmap of αCD4 (vs. IgG) granulomas.

(H) Schematic representation of the differential L-R pairs in αCD4 granulomas from (G).

(I) Heatmap of IgG (vs. αCD4) granulomas.

(J) Schematic representation of differential L-R pairs specific to IgG granulomas from (I).

(K) Bar plot representation of differential *IL10-IL10RA/RB* interactions among IgG lesions, similar to that of (C).

See also Figure S5 and Table S9.

CellPress
OPEN ACCESS

healing (*MMP14*, *S100A4*), and pro-angiogenic (*PDGFC*) signaling[58–61] (Figures S5B and S5G). Outgoing mast cell signaling *IL7*, *TYROBP*, and *NECTIN2* largely targeted T, NK cell receivers expressing *TIGIT*, *IL7R*, *PECAM1*, *KLRD1*, suggesting immunoregulatory (*NECTIN2-TIGIT*) and homeostatic (*IL7-IL7R*) signaling axes among these cells. Our analysis also identified prominent B cell-macrophage communication via *TGFB1-SDC2* and *PTPRC-MRC1*, suggesting that B cells contributed to macrophage polarization in the granuloma.[62] Our subpopulation-specific cell-cell interaction analyses identified (1) several monocyte-derived and mast cell subpopulations contributed to these wound-healing and anti-inflammatory signaling pathways and (2) blunted type 1 immune network topologies (Figures S5F and S5G). There was also T, NK signaling to T, NK cells; these circuits were characterized by *IL10-IL10RA* and *IL7-IL7R*, suggesting that reinfection granulomas and the associated cytokine milieu and cellular composition promoted self-reinforcing immunosuppressive and homeostatic regulatory T cell interactions[63] (Figures 7D and 7E). A subpopulation-specific query of these cells revealed Tregs were the primary senders of *IL7* and targeted Tc17-like cells (Figure S5G). This analysis also demonstrated that *GZMK*^{hi} $T_{EM/PEX\text{-}like}$ were one of the putative *IL10* sender populations and primarily targeted *IL10RA S100A4*^{hi}, *CFP*^{hi} monocytes, Tc17-like, and T1T17-like cells. This analysis further defined a shift away from *IL10* innate sender cellular subsets toward *IL10* adaptive immune cell subpopulations: *GZMK*^{hi} $T_{EM/PEX\text{-}like}$ and NK-like cells, relative to naive granulomas, with these two T, NK cell subsets targeting several T, NK cells subsets (Tc17-like, T1T17-like, Tregs) (Figure 7F).

In the absence of CD4^{+} T cells, TB granulomas were again dominated by pro-inflammatory neutrophil-derived and type 1 IFN signaling (Figures 7G, 7H, S5H, and S5J–S5L). Outgoing neutrophil-derived signaling was enriched for type 1 immune signaling (*TNF*, *IL1B*, *CXCL10*), targeting macrophages and T, NK cells. There was a relative loss of mast cell signaling, including *IL7* signaling to *IL7R* expressing T, NK cells, suggesting a loss of homeostatic cycling of memory T, NK cell subsets compared with IgG (Figures 7I, 7J, S5J, and S5K). CD4-depletion lesions were further characterized by a relative loss of T, NK-T, NK signaling circuits involving sender ligands (*IL7*, *IL10*, *CD40LG*, *CD28*) and corresponding receiver receptors (*CD4*, *IL10RA*, *IL10RA,B*, *IL7R*, *ITGB1-2*) on T, NK cells (Figures 7G–7J, S5J, S5K, S5M, and S5N). Given the enrichment of *IL10* among coarse-grain T, NK cells, we queried all immune cell subpopulations to identify the putative *IL10* sender subpopulation(s), which revealed two terminally differentiated (*GZMK*^{hi} $T_{EM/PEX\text{-}like}$, $T_{EMRA\text{-}like}$) and one innate-like (NK-like) T cell subpopulation, as well as two monocyte-derived subpopulations (*S100A4*^{hi}, *CFP*^{hi} monocytes, *FABP4*^{hi}, and *MCEMP*^{hi} alveolar MΦ), which targeted nineteen immune cell subpopulations, including eight T, NK cell subpopulations, suggesting the presence of CD4^{+} T cells was necessary for *CD8* T, NK cell immunomodulation and regulation in TB granulomas (Figures 7K, S5O, and S5P). IgG granulomas also demonstrated robust B cell signaling compared with αCD4 lesions, which lacked B cell contributions to the granuloma cell-cell interactome (Figure 7I). Compared with αCD4 granulomas, IgG B cell sender ligands (*B2M*, *CD40*, *CD72*, *RPS19*, *TGFB1*) targeted four cell types, with two (*CD40*, *CD72*) of the five top-ranked ligands targeting T, NK cell receptors (*CD40LG*, *CD5*)—a potential consequence of CD4^{+} T cell depletion[64,65] (Figure 7J).

In summary, our systematic examination of the TB granuloma microenvironment following *Mtb* reinfection in the presence and absence of CD4 T cells delineated distinct cellular circuitries, presenting a spectrum of responses—from amplification to dampening of the host inflammatory response—and underscoring the balance of immune regulation associated with enhanced TB control.

DISCUSSION

The TB granuloma represents a perturbed immunological niche where tissue resident and nascently recruited cells work together against microenvironmental stressors (e.g., *Mtb*, cellular enrichment/depletion, fibrosis, necrosis, inflammation, and hypoxia) in an attempt to restore homeostasis. These responses can either promote bacterial control or dissemination and tissue damage or preservation.[66,67] The multi-modal analyses reported here revealed global shifts in cellular composition, gene programming, and *Mtb* dynamics in primary *Mtb* infection and reinfection and nominated mechanisms by which CD4^{+} T cells contributed to a restrictive immunological niche. Our study yielded insights into the cellular, molecular, and niche features that support anti-*Mtb* activity or promote maladaptive immunity following infection—most critically, that CD4^{+} T cells act as homeostatic regulators of inflammation. It also identified tissue-level cellular response mechanisms that can be targeted in future investigations for the development of improved prophylactics and cures.

Our high-dimensional examination of TB reinfection granulomas revealed underlying mechanisms governing granuloma cellularity and cytokine flux, as well as putative cell-cell interactions, providing insights into their roles in modulating anti-*Mtb* immunity. Illustratively, IgG lesions featured robust upregulation of immuno-modulatory and -regulatory genes among lymphocytes relative to those of naive granulomas, which appeared to be CD4 dependent. Cytokines canonically associated with protective TB immunity (*TNF*, *IFNG*) did not distinguish IgG granulomas from naive or αCD4 lesions. These data corroborated our previous[8] findings demonstrating that reinfected macaques show increased IL-10 and relatively lower TNF and IFN-γ production. Moreover, recent work assessing the efficacy of pulmonary mucosal BCG identifies IL-10^{+} T cells as the most robust correlate of protection.[68] In both studies,[8,68] the source of IL-10 production among T cells was unknown. Our present work expanded these findings, identifying several T, NK cell subpopulations, including terminally differentiated and cytotoxic *CD8*-enriched subpopulations as putative sources of *IL10* production in TB granulomas. Altogether, our data demonstrated a shift in reinfection granuloma cytokine flux, cellularity, and programming, with T, NK cells biasing toward *CD8*-enriched immunoregulatory phenotypes.

Our cell-cell interaction analyses identified roles for the immunoregulatory molecules *IL10* and *TIGIT*—expressed among T, NK cells—following *Mtb* reinfection. These molecules were absent among naive and CD4-depleted signaling networks, suggesting that following immune priming, CD8^{+} T, NK cells required CD4^{+}

T cell help to engage in self- and non-self-immunoregulatory circuits. The immunoregulatory molecule PD-1 and cyclophilin D (CypD)-mediated T cell metabolism promote host resistance to TB, with checkpoint inhibitors (PD-1 blockade) and CypD genetic blockade exacerbating TB disease and immunopathology via overproduction of IFN-γ and TNF-α from CD4$^+$ T cells, and elevate infiltrates of pro-inflammatory CD8$^+$ T cells.[69–72] This suggests that the immunoregulatory circuits we identified among reinfection T, NK cells may be necessary to balance granuloma equilibria and mitigate tissue damage. While PD-1, IL-10, TIGIT, and other immunosuppressive molecules may limit inflammatory pathophysiology associated with TB, they may also inadvertently foster a microenvironment conducive to *Mtb* persistence,[73–75] highlighting the importance of balanced interplay between IL-10 and other immunoregulatory molecules and type 1 immunity in anti-TB immunity.[76]

Our analyses revealed that the immunoregulatory molecules *TIGIT*, *IL10*, and *PDCD1* were upregulated in the *CD8$^+$ GZMK*^{hi} $T_{EM/PEX\text{-}like}$ T, NK cell subpopulation. A comparison of *GZMK*^{hi} $T_{EM/PEX\text{-}like}$ frequencies showed that immunologically primed (IgG) animals experience elevated recruitment (or retention) of *GZMK*^{hi} $T_{EM/PEX\text{-}like}$ cells relative to naive or αCD4 granulomas, suggesting that *GZMK*^{hi} $T_{EM/PEX\text{-}like}$ localization was CD4$^+$ T cell dependent. Although *GZMK*^{hi} $T_{EM/PEX\text{-}like}$ cells were enriched in granulomas with dampened type 1 immune cellularity and inflammatory response, scRNA-seq analyses of disparate pathologies and tissues suggest *GZMK* CD8$^+$ T cells promote and potentiate inflammatory sequelae.[33,77,78] Moreover, these scRNA-seq studies demonstrate that *GZMK* CD8$^+$ T cell receptors (TCRs) are highly clonal and restricted to sites of inflammation, suggesting that these cells differentiate at the site of disease or become differentiated before recruitment.[77,79] In TB, analyses of *GZMK* CD8$^+$ transcriptomes and their TCR repertoires demonstrated that clonally expanded *GZMK* CD8$^+$ cells are restricted to TB pleural fluid and absent in blood.[79] In our study, granuloma *GZMK*^{hi} $T_{EM/PEX\text{-}like}$ cells featured low gene expression of T cell migration factors and upregulated expression of genes canonically associated with chronic inflammation and immunoregulation potentially suggesting: (1) granuloma *GZMK*^{hi} $T_{EM/PEX\text{-}like}$ cells had intra-compartment/lesion migratory potential, and (2) *GZMK*^{hi} $T_{EM/PEX\text{-}like}$ cells acquired a terminally differentiated phenotype at the site of infection. Collectively, these data indicated that *GZMK*^{hi} $T_{EM/PEX\text{-}like}$ recruitment (or retention), differentiation, and state may be CD4$^+$ T cell dependent, further supporting a critical role for CD4$^+$ T cells in balancing pro- and anti-inflammatory immunity.

In addition to CD4$^+$ IFN-γ and TNF production, T cells secrete the immuno-modulatory cytokine IL-10, which dampens both adaptive and innate immunity.[80–82] Our cell-cell interaction analyses revealed a relative shift in the diversity of putative *IL10* senders among IgG (vs. naive) populations. These data demonstrated that naive granulomas were enriched for *IL10* senders of myeloid lineage, whereas IgG lesions were enriched for *IL10* T, NK cell senders. This suggested divergent regulatory axes of IL-10. Previously, we demonstrated *in vivo*[75] and *in silico*[83] that elevated macrophage-derived IL-10 during early *Mtb* infection in naive animals may contribute to *Mtb* persistence and modulate granuloma caseation. Concordant with these previous studies, our current data indicated that (1) elevated *IL10* expression among myeloid-derived subsets during early infection (naive animals) contributed to the formation of a multicellular niche permissive to *Mtb* growth, and (2) increased *IL10*, from T, NK cells, later during infection (IgG animals) may mitigate inflammatory sequelae.

In keeping with these findings, we observed reinfection (IgG) granulomas, enriched for *IL10* sender T, NK cells, experienced significant blunting of type 1 inflammation and type 1 IFN signaling, and decreased frequencies of M1- and interstitial-like macrophages relative to primary infection (naive) and αCD4 granulomas. Furthermore, our findings demonstrated that monocyte-derived transcriptomes in IgG granulomas showed downregulated type 1 chemokines (e.g., CXCL9–11) and IFN-γ and TNF response pathways—potentially a nuanced mechanism wherein the host attempted to achieve an equilibrium between protective immunity and tissue preservation.[84,85] This modulation, while potentially mitigating tissue damage, may have inadvertently fostered a tissue microenvironment permissive to *Mtb* growth and persistence: for example, our data demonstrated that reinfection (IgG) granulomas were enriched for M2-like (characterized by lipid-metabolizing gene programming) alveolar macrophages—a phagocyte population that has been shown to harbor *Mtb*, thus contributing to bacterial growth and dissemination.[86–88] However, our *Mtb* barcode data demonstrated reinfection (IgG) animals had reduced bacterial dissemination compared with naive animals, and αCD4 reinfection macaques had enhanced dissemination, highlighting the pivotal role of CD4$^+$ T cells in modulating an effective host response that can mitigate bacterial establishment and *Mtb* dissemination during reinfection.

Macrophage sensing and phagocytosis of *Mtb* during initial infection trigger the production of pro-inflammatory chemokines and cytokines that promote vascular permeability, upregulation of adhesion molecules, and subsequent neutrophil recruitment.[89,90] Our scRNA-seq analyses uncovered previously unappreciated neutrophil heterogeneity—cell types that have been underrepresented in droplet-based single-cell profiling of TB[91,92]—in TB granulomas, including the identification of two neutrophil subsets with differential pathway activation and phenotypic signatures. Our data demonstrated that hypoxia- and inflammatory-enriched naive and αCD4 granulomas have significant neutrophilia and significant induction of an IFN-responsive module associated with active TB, which may contribute to tissue inflammation, lung damage, and the formation of an *Mtb*-permissive niche, thus promoting *Mtb* growth and dissemination.[53,55] IgG granulomas—characterized by reduced neutrophilia—did not support the same level of bacterial growth or dissemination as naive lesions and inhibition of *Mtb* growth, and dissemination post-reinfection was at least partially CD4$^+$ T cell-dependent and independent of CD4$^+$ T cell-mediated induction of myeloid IFN-γ and TNF response pathways.

In line with our findings, which demonstrated that early (naive NHP) granulomas feature robust type 1 immune induction (e.g., *IL1B*, *CXCL9–11*) and signaling, previous research shows that the chemokines CXCL9–11 are enriched among 4 week primary granulomas and that those granulomas feature elevated CXCR3$^+$ T cell frequencies—putative sources of IFN-γ and TNF.[18,93,94] These findings highlight the critical role of

CellPress
OPEN ACCESS

CXCL9–11 during early *Mtb* infection (before immune priming), where they promote the recruitment of lymphocyte populations ($CXCR3^+$ T cells); however, overexpression may drive TB sequelae via pro-inflammatory myeloid-T, NK cell circuits, which potentiate pro-inflammatory response mechanisms and bacterial dissemination.[91,95,96] Indeed, the chemokines CXCL9–11 are potential biomarkers of TB severity.[97–101] While there is substantial evidence suggesting that excessive CXCL9–11 may contribute to the early anti-*Mtb* immunity or immunopathology, other studies suggest that CXCL9–11 may serve as markers of trained immunity following BCG vaccination and a correlate of protection.[102] Future work in vaccinated macaques could help determine whether, and possibly when, elevated type 1 immune signaling is indicative of innate training or a correlate of TB pathophysiology and chronic inflammatory stimuli.

In summary, we provided an in-depth characterization of primary infection, reinfection, and $CD4^+$ T cell-depleted reinfection macaque granulomas, identifying potential mechanisms by which $CD4^+$ T cells contribute to anti-mycobacterial immunity. Our analyses revealed cellular networks in which $CD4^+$ T cells regulated pro- and anti-inflammatory gene programming and cell-cell signaling networks to limit inflammatory sequelae, as well as bacterial establishment, growth, and dissemination. These findings expanded beyond the limited purview of the TB "central dogma," demonstrating that $CD4^+$ T cells act not only as effectors secreting IFN-γ and TNF but also as homeostatic regulators, orchestrating both pro- and anti-inflammatory immunity, thus leading to a more nuanced understanding of protective immunity against TB disease and a broader understanding of how $CD4^+$ T cells modulate the immune response in reinfection events.

Limitations of the study

We acknowledge inherent limitations associated with our design and experimental power, including: (1) 4- to 5-month drug treatment to clear primary infection, (2) limited numbers of NHPs and granulomas for scRNA-seq, and (3) few IgG granulomas available for histological analysis. The robust protection against reinfection among IgG macaques resulted in the formation of relatively few library S granulomas, limiting the number of samples available for histological analysis.

Our study design did not enable us to compare naive and αCD4 groups directly since we could not disentangle shifts due to immunological priming from those due to $CD4^+$ T cell depletion. The αCD4 cohort had significantly higher bacterial burden than the IgG group, so it is possible that some aspects of the increased pro-inflammatory milieu in αCD4 granulomas may have been a consequence of increased CFU rather than a direct effect of $CD4^+$ T cell depletion. However, an analysis of the few IgG and αCD4 granulomas with matched CFU suggested that $CD4^+$ T cell depletion drove the described features.

A final limitation of our study pertains to the putative cell-cell interactions identified from dissociated tissues. Current computational cell-cell/ligand-receptor (L-R) frameworks[56,103–105] are linear models, can only predict how cell type 1 interacts with cell type 2, and are unable to account for multicellular interactions. Thus, it is possible that one cell type (i.e., cell type 3) indirectly modulated immunoregulatory network activity and cellular recruitment or retention in granulomas.

RESOURCE AVAILABILITY

Lead contact

Further information and requests for resources and reagents should be directed to and will be fulfilled by the lead contact Dr. JoAnne Flynn (joanne@pitt.edu).

Materials availability

This study did not generate new unique reagents.

Data and code availability

scRNA-seq data are publicly available for download and visualization via the Alexandria and Broad Institute Single Cell Portal from the date of publication. Accession numbers and links are listed in the key resources table. All the data generated in support of the reported findings can be found at Fairdomhub: https://fairdomhub.org/studies/1239; this repository contains scRNA-seq, bacterial barcoding, CFU, flow cytometry, and PET-CT imaging data. All original code has been deposited at Zenodo and GitHub and is publicly available as of the date of publication – see key resources table. Any additional information required to reanalyze the data from this study is available from the lead contact upon request.

ACKNOWLEDGMENTS

We extend our appreciation to the technical and veterinary teams of the Flynn, Shalek, and Fortune laboratories for their dedication and assistance. We are indebted to the University of Pittsburgh's Division of Laboratory Animal Research and Unified Flow Core for animal husbandry and support. We are grateful to the NIH Nonhuman Primate Reagent Resource for providing the CD4-depleting mAb (ORIP P40 OD028116, U24 AI126683). This work was supported in part by the Bill and Melinda Gates Foundation BMGF INV-027498 (A.K.S.), NIH R01 AI114674 (J.L.F. and S.M.F.), Harvard University Center for AIDS Research P30 AI060354 (J.M.R.), Harvard Clinical and Translational Science Center K12 TR002542 (J.M.R.), NIH contract IMPAc-TB 75N93019C00071 (S.M.F., J.L.F., and A.K.S.), and NIH NIAID UC7AI180311 supporting operations of the University of Pittsburgh Regional Biocontainment Laboratory.

AUTHOR CONTRIBUTIONS

Conceptualization, J.L.F., S.M.F., and A.K.S.; data curation, J.D.B., S.K.C.G., M.C., H.P.G., and R.F.-O.; formal analysis, J.D.B., S.K.C.G., S.K.N., P.M., M.C., and Q.D.W.; investigation, J.D.B., S.K.C.G., S.K.N., P.M., M.C., D.M., S.N., J.M.R., and H.P.G.; visualization, J.D.B., P.M., and M.C.; funding acquisition, J.L.F., S.M.F., and A.K.S.; supervision, J.L.F., S.M.F., A.K.S., and B.B.; writing – original draft, J.D.B., D.M., J.L.F., S.M.F., and A.K.S.; writing – review & editing, J.D.B., S.K.C.G., S.K.N., M.C., D.M., S.N., J.M.R., J.L.F., S.M.F., and A.K.S.

DECLARATION OF INTERESTS

A.K.S. reports compensation for consulting and/or scientific advisory board membership from Honeycomb Biotechnologies, Cellarity, Ochre Bio, Relation Therapeutics, Fog Pharma, Passkey Therapeutics, IntrECate Biotherapeutics, Bio-Rad Laboratories, and Dahlia Biosciences unrelated to this work. S.M.F. reports compensation for board of directors' membership from Oxford Nanopore unrelated to this work. J.L.F. reports compensation for consulting for Janssen Inc. and scientific advisory board membership for the Nonhuman Primate Research Resource unrelated to this work. After submission of this publication, J.M.R. began employment with Merck & Co., Cambridge, MA, USA. He did not conduct work on this publication after his employment at Merck & Co.

STAR★METHODS

Detailed methods are provided in the online version of this paper and include the following:

- KEY RESOURCES TABLE

- EXPERIMENTAL MODEL AND STUDY PARTICIPANT DETAILS
  - Research animals
- METHOD DETAILS
  - Animals, infections, CD4 depletion, and disease tracking by PET CT
  - Necropsy procedures
  - Isolation of genomic DNA from bacteria
  - Library identification
  - Flow cytometry
  - Antibody validation
  - Single-cell RNA-sequencing (scRNA-seq) and alignment
- QUANTIFICATION AND STATISTICAL ANALYSIS
  - Statistical analysis on macaque samples (depletion and CFU)
  - Data processing and quality control
  - Dimensionality reduction and batch correction
  - Cell clustering and annotation
  - Differential abundance analysis of scRNA-seq cell type and subset frequencies
  - Differential expression analysis
  - Pseudobulk differential expression analysis
  - Pathway enrichment analysis
  - Differential cell-cell and receptor-ligand analyses

SUPPLEMENTAL INFORMATION

Supplemental information can be found online at https://doi.org/10.1016/j.immuni.2024.08.002.

Received: December 22, 2023
Revised: June 3, 2024
Accepted: August 1, 2024
Published: August 29, 2024

REFERENCES

1. Ahmed, A., Rakshit, S., Adiga, V., Dias, M., Dwarkanath, P., D'Souza, G., and Vyakarnam, A. (2021). A century of BCG: impact on tuberculosis control and beyond. Immunol. Rev. *301*, 98–121. https://doi.org/10.1111/imr.12968.
2. Lange, C., Aaby, P., Behr, M.A., Donald, P.R., Kaufmann, S.H.E., Netea, M.G., and Mandalakas, A.M. (2022). 100 years of Mycobacterium bovis bacille Calmette-Guérin. Lancet Infect. Dis. *22*, e2–e12. https://doi.org/10.1016/S1473-3099(21)00403-5.
3. Lawn, S.D., and Zumla, A.I. (2011). Tuberculosis. Lancet *378*, 57–72. https://doi.org/10.1016/S0140-6736(10)62173-3.
4. Millet, J.P., Orcau, A., de Olalla, P.G., Casals, M., Rius, C., and Caylà, J.A. (2009). Tuberculosis recurrence and its associated risk factors among successfully treated patients. J. Epidemiol. Community Health *63*, 799–804. https://doi.org/10.1136/jech.2008.077560.
5. Shen, X., Yang, C., Wu, J., Lin, S., Gao, X., Wu, Z., Tian, J., Gan, M., Luo, T., Wang, L., et al. (2017). Recurrent tuberculosis in an urban area in China: Relapse or exogenous reinfection? Tuberculosis (Edinb) *103*, 97–104. https://doi.org/10.1016/j.tube.2017.01.007.
6. Verver, S., Warren, R.M., Beyers, N., Richardson, M., Van Der Spuy, G.D., Borgdorff, M.W., Enarson, D.A., Behr, M.A., and Van Helden, P.D. (2005). Rate of Reinfection Tuberculosis after Successful Treatment Is Higher than Rate of New Tuberculosis. Am. J. Respir. Crit. Care Med. *171*, 1430–1435. https://doi.org/10.1164/rccm.200409-1200OC.
7. Andrews, J.R., Noubary, F., Walensky, R.P., Cerda, R., Losina, E., and Horsburgh, C.R. (2012). Risk of Progression to Active Tuberculosis Following Reinfection With Mycobacterium tuberculosis. Clin. Infect. Dis. *54*, 784–791. https://doi.org/10.1093/cid/cir951.
8. Cadena, A.M., Hopkins, F.F., Maiello, P., Carey, A.F., Wong, E.A., Martin, C.J., Gideon, H.P., DiFazio, R.M., Andersen, P., Lin, P.L., et al. (2018). Concurrent infection with Mycobacterium tuberculosis confers robust protection against secondary infection in macaques. PLoS Pathog. *14*, e1007305. https://doi.org/10.1371/journal.ppat.1007305.
9. Ganchua, S.K., Maiello, P., Chao, M., Hopkins, F., Mugahid, D., Lin, P.L., Fortune, S.M., and Flynn, J.L. (2024). Antibiotic treatment modestly reduces protection against *Mycobacterium tuberculosis* reinfection in macaques. Infect. Immun. *92*, e0053523. https://doi.org/10.1128/iai.00535-23.
10. Nemeth, J., Olson, G.S., Rothchild, A.C., Jahn, A.N., Mai, D., Duffy, F.J., Delahaye, J.L., Srivatsan, S., Plumlee, C.R., Urdahl, K.B., et al. (2020). Contained Mycobacterium tuberculosis infection induces concomitant and heterologous protection. PLoS Pathog. *16*, e1008655. https://doi.org/10.1371/journal.ppat.1008655.
11. Abel, L., Fellay, J., Haas, D.W., Schurr, E., Srikrishna, G., Urbanowski, M., Chaturvedi, N., Srinivasan, S., Johnson, D.H., and Bishai, W.R. (2018). Genetics of human susceptibility to active and latent tuberculosis: present knowledge and future perspectives. Lancet Infect. Dis. *18*, e64–e75. https://doi.org/10.1016/S1473-3099(17)30623-0.
12. Cohen, S.B., Gern, B.H., and Urdahl, K.B. (2022). The Tuberculous Granuloma and Preexisting Immunity. Annu. Rev. Immunol. *40*, 589–614. https://doi.org/10.1146/annurev-immunol-093019-125148.
13. Coscolla, M., and Gagneux, S. (2014). Consequences of genomic diversity in Mycobacterium tuberculosis. Semin. Immunol. *26*, 431–444. https://doi.org/10.1016/j.smim.2014.09.012.
14. Galagan, J.E. (2014). Genomic insights into tuberculosis. Nat. Rev. Genet. *15*, 307–320. https://doi.org/10.1038/nrg3664.
15. Kwan, C.K., and Ernst, J.D. (2011). HIV and Tuberculosis: a Deadly Human Syndemic. Clin. Microbiol. Rev. *24*, 351–376. https://doi.org/10.1128/CMR.00042-10.
16. Lin, P.L., Rutledge, T., Green, A.M., Bigbee, M., Fuhrman, C., Klein, E., and Flynn, J.L. (2012). CD4 T Cell Depletion Exacerbates Acute *Mycobacterium tuberculosis* While Reactivation of Latent Infection Is Dependent on Severity of Tissue Depletion in Cynomolgus Macaques. AIDS Res. Hum. Retroviruses *28*, 1693–1702. https://doi.org/10.1089/AID.2012.0028.
17. Scanga, C.A., Mohan, V.P., Yu, K., Joseph, H., Tanaka, K., Chan, J., and Flynn, J.L. (2000). Depletion of CD4(+) T Cells Causes Reactivation of Murine Persistent Tuberculosis Despite Continued Expression of Interferon Gamma and Nitric Oxide Synthase 2. J. Exp. Med. *192*, 347–358. https://doi.org/10.1084/jem.192.3.347.
18. Gideon, H.P., Hughes, T.K., Tzouanas, C.N., Wadsworth, M.H., Tu, A.A., Gierahn, T.M., Peters, J.M., Hopkins, F.F., Wei, J.-R., Kummerlowe, C., et al. (2022). Multimodal profiling of lung granulomas in macaques reveals cellular correlates of tuberculosis control. Immunity *55*, 827–846.e10. https://doi.org/10.1016/j.immuni.2022.04.004.
19. Lin, P.L., Ford, C.B., Coleman, M.T., Myers, A.J., Gawande, R., Ioerger, T., Sacchettini, J., Fortune, S.M., and Flynn, J.L. (2014). Sterilization of granulomas is common in active and latent tuberculosis despite within-host variability in bacterial killing. Nat. Med. *20*, 75–79. https://doi.org/10.1038/nm.3412.
20. Diedrich, C.R., Rutledge, T., Maiello, P., Baranowski, T.M., White, A.G., Borish, H.J., Karell, P., Hopkins, F., Brown, J., Fortune, S.M., et al. (2020). SIV and Mycobacterium tuberculosis synergy within the granuloma accelerates the reactivation pattern of latent tuberculosis. PLoS Pathog. *16*, e1008413. https://doi.org/10.1371/journal.ppat.1008413.
21. Esmail, H., Riou, C., Du Bruyn, E.D., Lai, R.P.-J., Harley, Y.X.R., Meintjes, G., Wilkinson, K.A., and Wilkinson, R.J. (2018). The Immune Response to *Mycobacterium tuberculosis* in HIV-1-Coinfected Persons. Annu. Rev. Immunol. *36*, 603–638. https://doi.org/10.1146/annurev-immunol-042617-053420.
22. Foreman, T.W., Mehra, S., LoBato, D.N., Malek, A., Alvarez, X., Golden, N.A., Bucşan, A.N., Didier, P.J., Doyle-Meyers, L.A., Russell-Lodrigue, K.E., et al. (2016). $CD4^+$ T-cell-independent mechanisms suppress reactivation of latent tuberculosis in a macaque model of HIV coinfection. Proc. Natl. Acad. Sci. USA *113*, E5636–E5644. https://doi.org/10.1073/pnas.1611987113.

CellPress
OPEN ACCESS

23. Foreman, T.W., Nelson, C.E., Kauffman, K.D., Lora, N.E., Vinhaes, C.L., Dorosky, D.E., Sakai, S., Gomez, F., Fleegle, J.D., Parham, M., et al. (2022). CD4 T cells are rapidly depleted from tuberculosis granulomas following acute SIV co-infection. Cell Rep. *39*, 110896. https://doi.org/10.1016/j.celrep.2022.110896.

24. Larson, E.C., Ellis-Connell, A.L., Rodgers, M.A., Gubernat, A.K., Gleim, J.L., Moriarty, R.V., Balgeman, A.J., Ameel, C.L., Jauro, S., Tomko, J.A., et al. (2023). Intravenous Bacille Calmette–Guérin vaccination protects simian immunodeficiency virus-infected macaques from tuberculosis. Nat. Microbiol. *8*, 2080–2092. https://doi.org/10.1038/s41564-023-01503-x.

25. Martin, C.J., Cadena, A.M., Leung, V.W., Lin, P.L., Maiello, P., Hicks, N., Chase, M.R., Flynn, J.L., and Fortune, S.M. (2017). Digitally Barcoding *Mycobacterium* tuberculosis Reveals In Vivo Infection Dynamics in the Macaque Model of Tuberculosis. mBio *8*, e00312-17. https://doi.org/10.1128/mBio.00312-17.

26. Cosma, C.L., Humbert, O., and Ramakrishnan, L. (2004). Superinfecting mycobacteria home to established tuberculous granulomas. Nat. Immunol. *5*, 828–835. https://doi.org/10.1038/ni1091.

27. Hughes, T.K., Wadsworth, M.H., Gierahn, T.M., Do, T., Weiss, D., Andrade, P.R., Ma, F., De Andrade Silva, B.J., Shao, S., Tsoi, L.C., et al. (2020). Second-Strand Synthesis-Based Massively Parallel scRNA-Seq Reveals Cellular States and Molecular Features of Human Inflammatory Skin Pathologies. Immunity *53*, 878–894.e7. https://doi.org/10.1016/j.immuni.2020.09.015.

28. Sikkema, L., Ramírez-Suástegui, C., Strobl, D.C., Gillett, T.E., Zappia, L., Madissoon, E., Markov, N.S., Zaragosi, L.-E., Ji, Y., Ansari, M., et al. (2023). An integrated cell atlas of the lung in health and disease. Nat. Med. *29*, 1563–1577. https://doi.org/10.1038/s41591-023-02327-2.

29. Büttner, M., Ostner, J., Müller, C.L., Theis, F.J., and Schubert, B. (2021). scCODA is a Bayesian model for compositional single-cell data analysis. Nat. Commun. *12*, 6876. https://doi.org/10.1038/s41467-021-27150-6.

30. Smillie, C.S., Biton, M., Ordovas-Montanes, J., Sullivan, K.M., Burgin, G., Graham, D.B., Herbst, R.H., Rogel, N., Slyper, M., Waldman, J., et al. (2019). Intra- and Inter-cellular Rewiring of the Human Colon during Ulcerative Colitis. Cell *178*, 714–730.e22. https://doi.org/10.1016/j.cell.2019.06.029.

31. Tabula Muris Consortium (2020). A single-cell transcriptomic atlas characterizes ageing tissues in the mouse. Nature *583*, 590–595. https://doi.org/10.1038/s41586-020-2496-1.

32. Szabo, P.A., Levitin, H.M., Miron, M., Snyder, M.E., Senda, T., Yuan, J., Cheng, Y.L., Bush, E.C., Dogra, P., Thapa, P., et al. (2019). Single-cell transcriptomics of human T cells reveals tissue and activation signatures in health and disease. Nat. Commun. *10*, 4706. https://doi.org/10.1038/s41467-019-12464-3.

33. Zheng, L., Qin, S., Si, W., Wang, A., Xing, B., Gao, R., Ren, X., Wang, L., Wu, X., Zhang, J., et al. (2021). Pan-cancer single-cell landscape of tumor-infiltrating T cells. Science *374*, abe6474. https://doi.org/10.1126/science.abe6474.

34. Galletti, G., De Simone, G., Mazza, E.M.C., Puccio, S., Mezzanotte, C., Bi, T.M., Davydov, A.N., Metsger, M., Scamardella, E., Alvisi, G., et al. (2020). Two subsets of stem-like CD8+ memory T cell progenitors with distinct fate commitments in humans. Nat. Immunol. *21*, 1552–1562. https://doi.org/10.1038/s41590-020-0791-5.

35. Mogilenko, D.A., Shpynov, O., Andhey, P.S., Arthur, L., Swain, A., Esaulova, E., Brioschi, S., Shchukina, I., Kerndl, M., Bambouskova, M., et al. (2021). Comprehensive Profiling of an Aging Immune System Reveals Clonal GZMK+ CD8+ T Cells as Conserved Hallmark of Inflammaging. Immunity *54*, 99–115.e12. https://doi.org/10.1016/j.immuni.2020.11.005.

36. Buggert, M., Vella, L.A., Nguyen, S., Wu, V.H., Chen, Z., Sekine, T., Perez-Potti, A., Maldini, C.R., Manne, S., Darko, S., et al. (2020). The Identity of Human Tissue-Emigrant CD8+ T Cells. Cell *183*, 1946–1961.e15. https://doi.org/10.1016/j.cell.2020.11.019.

37. Gerlach, C., Moseman, E.A., Loughhead, S.M., Alvarez, D., Zwijnenburg, A.J., Waanders, L., Garg, R., De La Torre, J.C., and Von Andrian, U.H. (2016). The Chemokine Receptor CX3CR1 Defines Three Antigen-Experienced CD8 T Cell Subsets with Distinct Roles in Immune Surveillance and Homeostasis. Immunity *45*, 1270–1284. https://doi.org/10.1016/j.immuni.2016.10.018.

38. Finak, G., McDavid, A., Yajima, M., Deng, J., Gersuk, V., Shalek, A.K., Slichter, C.K., Miller, H.W., McElrath, M.J., Prlic, M., et al. (2015). MAST: a flexible statistical framework for assessing transcriptional changes and characterizing heterogeneity in single-cell RNA sequencing data. Genome Biol. *16*, 278. https://doi.org/10.1186/s13059-015-0844-5.

39. Kotliar, D., Lin, A.E., Logue, J., Hughes, T.K., Khoury, N.M., Raju, S.S., Wadsworth, M.H., Chen, H., Kurtz, J.R., Dighero-Kemp, B., et al. (2020). Single-Cell Profiling of Ebola Virus Disease In Vivo Reveals Viral and Host Dynamics. Cell *183*, 1383–1401.e19. https://doi.org/10.1016/j.cell.2020.10.002.

40. Vignali, P.D.A., DePeaux, K., Watson, M.J., Ye, C., Ford, B.R., Lontos, K., McGaa, N.K., Scharping, N.E., Menk, A.V., Robson, S.C., et al. (2023). Hypoxia drives CD39-dependent suppressor function in exhausted T cells to limit antitumor immunity. Nat. Immunol. *24*, 267–279. https://doi.org/10.1038/s41590-022-01379-9.

41. Schubert, M., Klinger, B., Klünemann, M., Sieber, A., Uhlitz, F., Sauer, S., Garnett, M.J., Blüthgen, N., and Saez-Rodriguez, J. (2018). Perturbation-response genes reveal signaling footprints in cancer gene expression. Nat. Commun. *9*, 20. https://doi.org/10.1038/s41467-017-02391-6.

42. Voron, T., Colussi, O., Marcheteau, E., Pernot, S., Nizard, M., Pointet, A.-L., Latreche, S., Bergaya, S., Benhamouda, N., Tanchot, C., et al. (2015). VEGF-A modulates expression of inhibitory checkpoints on CD8+ T cells in tumors. J. Exp. Med. *212*, 139–148. https://doi.org/10.1084/jem.20140559.

43. Stephen, T.L., Payne, K.K., Chaurio, R.A., Allegrezza, M.J., Zhu, H., Perez-Sanz, J., Perales-Puchalt, A., Nguyen, J.M., Vara-Ailor, A.E., Eruslanov, E.B., et al. (2017). SATB1 Expression Governs Epigenetic Repression of PD-1 in Tumor-Reactive T Cells. Immunity *46*, 51–64. https://doi.org/10.1016/j.immuni.2016.12.015.

44. Huynh, J.P., Lin, C.-C., Kimmey, J.M., Jarjour, N.N., Schwarzkopf, E.A., Bradstreet, T.R., Shchukina, I., Shpynov, O., Weaver, C.T., Taneja, R., et al. (2018). Bhlhe40 is an essential repressor of IL-10 during *Mycobacterium tuberculosis* infection. J. Exp. Med. *215*, 1823–1838. https://doi.org/10.1084/jem.20171704.

45. Gago, G., Diacovich, L., and Gramajo, H. (2018). Lipid metabolism and its implication in mycobacteria–host interaction. Curr. Opin. Microbiol. *41*, 36–42. https://doi.org/10.1016/j.mib.2017.11.020.

46. Ravesloot-Chávez, M.M., Van Dis, E., and Stanley, S.A. (2021). The Innate Immune Response to *Mycobacterium tuberculosis* Infection. Annu. Rev. Immunol. *39*, 611–637. https://doi.org/10.1146/annurev-immunol-093019-010426.

47. Montaldo, E., Lusito, E., Bianchessi, V., Caronni, N., Scala, S., Basso-Ricci, L., Cantaffa, C., Masserdotti, A., Barilaro, M., Barresi, S., et al. (2022). Cellular and transcriptional dynamics of human neutrophils at steady state and upon stress. Nat. Immunol. *23*, 1470–1483. https://doi.org/10.1038/s41590-022-01311-1.

48. Carmona-Rivera, C., Zhao, W., Yalavarthi, S., and Kaplan, M.J. (2015). Neutrophil extracellular traps induce endothelial dysfunction in systemic lupus erythematosus through the activation of matrix metalloproteinase-2. Ann. Rheum. Dis. *74*, 1417–1424. https://doi.org/10.1136/annrheumdis-2013-204837.

49. Gasperini, S., Marchi, M., Calzetti, F., Laudanna, C., Vicentini, L., Olsen, H., Murphy, M., Liao, F., Farber, J., and Cassatella, M.A. (1999). Gene Expression and Production of the Monokine Induced by IFN-γ (MIG), IFN-Inducible T Cell α Chemoattractant (I-TAC), and IFN-γ-Inducible Protein-10 (IP-10) Chemokines by Human Neutrophils. J. Immunol. *162*, 4928–4937. https://doi.org/10.4049/jimmunol.162.8.4928.

50. Seiler, P., Aichele, P., Bandermann, S., Hauser, A.E., Lu, B., Gerard, N.P., Gerard, C., Ehlers, S., Mollenkopf, H.J., and Kaufmann, S.H.E. (2003). Early granuloma formation after aerosol Mycobacterium tuberculosis infection is regulated by neutrophils via CXCR3-signaling chemokines. Eur. J. Immunol. *33*, 2676–2686. https://doi.org/10.1002/eji.200323956.

51. Xie, X., Shi, Q., Wu, P., Zhang, X., Kambara, H., Su, J., Yu, H., Park, S.-Y., Guo, R., Ren, Q., et al. (2020). Single-cell transcriptome profiling reveals neutrophil heterogeneity in homeostasis and infection. Nat. Immunol. *21*, 1119–1133. https://doi.org/10.1038/s41590-020-0736-z.

52. Ichikawa, A., Kuba, K., Morita, M., Chida, S., Tezuka, H., Hara, H., Sasaki, T., Ohteki, T., Ranieri, V.M., Dos Santos, C.C., et al. (2013). CXCL10-CXCR3 Enhances the Development of Neutrophil-mediated Fulminant Lung Injury of Viral and Nonviral Origin. Am. J. Respir. Crit. Care Med. *187*, 65–77. https://doi.org/10.1164/rccm.201203-0508OC.

53. Berry, M.P.R., Graham, C.M., McNab, F.W., Xu, Z., Bloch, S.A.A., Oni, T., Wilkinson, K.A., Banchereau, R., Skinner, J., Wilkinson, R.J., et al. (2010). An interferon-inducible neutrophil-driven blood transcriptional signature in human tuberculosis. Nature *466*, 973–977. https://doi.org/10.1038/nature09247.

54. Dallenga, T., Repnik, U., Corleis, B., Eich, J., Reimer, R., Griffiths, G.W., and Schaible, U.E. (2017). M. tuberculosis-Induced Necrosis of Infected Neutrophils Promotes Bacterial Growth Following Phagocytosis by Macrophages. Cell Host Microbe *22*, 519–530.e3. https://doi.org/10.1016/j.chom.2017.09.003.

55. Lovewell, R.R., Baer, C.E., Mishra, B.B., Smith, C.M., and Sassetti, C.M. (2021). Granulocytes act as a niche for Mycobacterium tuberculosis growth. Mucosal Immunol. *14*, 229–241. https://doi.org/10.1038/s41385-020-0300-z.

56. Browaeys, R., Gilis, J., Sang-Aram, C., De Bleser, P., Hoste, L., Tavernier, S., Lambrechts, D., Seurinck, R., and Saeys, Y. (2023). MultiNicheNet: a flexible framework for differential cell-cell communication analysis from multi-sample multi-condition single-cell transcriptomics data. Preprint at bioRxiv. https://doi.org/10.1101/2023.06.13.544751.

57. Moreira-Teixeira, L., Stimpson, P.J., Stavropoulos, E., Hadebe, S., Chakravarty, P., Ioannou, M., Aramburu, I.V., Herbert, E., Priestnall, S.L., Suarez-Bonnet, A., et al. (2020). Type I IFN exacerbates disease in tuberculosis-susceptible mice by inducing neutrophil-mediated lung inflammation and NETosis. Nat. Commun. *11*, 5566. https://doi.org/10.1038/s41467-020-19412-6.

58. Ackerman, J.E., Nichols, A.E., Studentsova, V., Best, K.T., Knapp, E., and Loiselle, A.E. (2019). Cell non-autonomous functions of S100a4 drive fibrotic tendon healing. eLife *8*, e45342. https://doi.org/10.7554/eLife.45342.

59. Atkinson, J.J., Toennies, H.M., Holmbeck, K., and Senior, R.M. (2007). Membrane type 1 matrix metalloproteinase is necessary for distal airway epithelial repair and keratinocyte growth factor receptor expression after acute injury. Am. J. Physiol. Lung Cell. Mol. Physiol. *293*, L600–L610. https://doi.org/10.1152/ajplung.00028.2007.

60. Rashidi, M., Bandala-Sanchez, E., Lawlor, K.E., Zhang, Y., Neale, A.M., Vijayaraj, S.L., O'Donoghue, R., Wentworth, J.M., Adams, T.E., Vince, J.E., et al. (2018). CD52 inhibits Toll-like receptor activation of NF-κB and triggers apoptosis to suppress inflammation. Cell Death Differ. *25*, 392–405. https://doi.org/10.1038/cdd.2017.173.

61. Suzuki, M., Tachibana, I., Takeda, Y., He, P., Minami, S., Iwasaki, T., Kida, H., Goya, S., Kijima, T., Yoshida, M., et al. (2009). Tetraspanin CD9 Negatively Regulates Lipopolysaccharide-Induced Macrophage Activation and Lung Inflammation. J. Immunol. *182*, 6485–6493. https://doi.org/10.4049/jimmunol.0802797.

62. Gong, D., Shi, W., Yi, S.J., Chen, H., Groffen, J., and Heisterkamp, N. (2012). TGFβ signaling plays a critical role in promoting alternative macrophage activation. BMC Immunol. *13*, 31. https://doi.org/10.1186/1471-2172-13-31.

63. Sun, J., Cardani, A., Sharma, A.K., Laubach, V.E., Jack, R.S., Müller, W., and Braciale, T.J. (2011). Autocrine Regulation of Pulmonary Inflammation by Effector T-Cell Derived IL-10 during Infection with Respiratory Syncytial Virus. PLoS Pathog. *7*, e1002173. https://doi.org/10.1371/journal.ppat.1002173.

64. Crotty, S. (2011). Follicular Helper CD4 T Cells (T_{FH}). Annu. Rev. Immunol. *29*, 621–663. https://doi.org/10.1146/annurev-immunol-031210-101400.

65. Sangster, M.Y., Riberdy, J.M., Gonzalez, M., Topham, D.J., Baumgarth, N., and Doherty, P.C. (2003). An Early CD4+ T Cell–dependent Immunoglobulin A Response to Influenza Infection in the Absence of Key Cognate T–B Interactions. J. Exp. Med. *198*, 1011–1021. https://doi.org/10.1084/jem.20021745.

66. Pagán, A.J., and Ramakrishnan, L. (2014). Immunity and Immunopathology in the Tuberculous Granuloma. Cold Spring Harb. Perspect. Med. *5*, a018499. https://doi.org/10.1101/cshperspect.a018499.

67. Pagán, A.J., and Ramakrishnan, L. (2018). The Formation and Function of Granulomas. Annu. Rev. Immunol. *36*, 639–665. https://doi.org/10.1146/annurev-immunol-032712-100022.

68. Dijkman, K., Sombroek, C.C., Vervenne, R.A.W., Hofman, S.O., Boot, C., Remarque, E.J., Kocken, C.H.M., Ottenhoff, T.H.M., Kondova, I., Khayum, M.A., et al. (2019). Prevention of tuberculosis infection and disease by local BCG in repeatedly exposed rhesus macaques. Nat. Med. *25*, 255–262. https://doi.org/10.1038/s41591-018-0319-9.

69. Barber, D.L., Sakai, S., Kudchadkar, R.R., Fling, S.P., Day, T.A., Vergara, J.A., Ashkin, D., Cheng, J.H., Lundgren, L.M., Raabe, V.N., et al. (2019). Tuberculosis following PD-1 blockade for cancer immunotherapy. Sci. Transl. Med. *11*, eaat2702. https://doi.org/10.1126/scitranslmed.aat2702.

70. Kauffman, K.D., Sakai, S., Lora, N.E., Namasivayam, S., Baker, P.J., Kamenyeva, O., Foreman, T.W., Nelson, C.E., Oliveira-de-Souza, D., Vinhaes, C.L., et al. (2021). PD-1 blockade exacerbates *Mycobacterium tuberculosis* infection in rhesus macaques. Sci. Immunol. *6*, eabf3861. https://doi.org/10.1126/sciimmunol.abf3861.

71. Sakai, S., Kauffman, K.D., Sallin, M.A., Sharpe, A.H., Young, H.A., Ganusov, V.V., and Barber, D.L. (2016). CD4 T Cell-Derived IFN-γ Plays a Minimal Role in Control of Pulmonary Mycobacterium tuberculosis Infection and Must Be Actively Repressed by PD-1 to Prevent Lethal Disease. PLoS Pathog. *12*, e1005667. https://doi.org/10.1371/journal.ppat.1005667.

72. Tzelepis, F., Blagih, J., Khan, N., Gillard, J., Mendonca, L., Roy, D.G., Ma, E.H., Joubert, P., Jones, R.G., and Divangahi, M. (2018). Mitochondrial cyclophilin D regulates T cell metabolic responses and disease tolerance to tuberculosis. Sci. Immunol. *3*, eaar4135. https://doi.org/10.1126/sciimmunol.aar4135.

73. Redford, P.S., Boonstra, A., Read, S., Pitt, J., Graham, C., Stavropoulos, E., Bancroft, G.J., and O'Garra, A. (2010). Enhanced protection to Mycobacterium tuberculosis infection in IL-10-deficient mice is accompanied by early and enhanced Th1 responses in the lung. Eur. J. Immunol. *40*, 2200–2210. https://doi.org/10.1002/eji.201040433.

74. Redford, P.S., Murray, P.J., and O'Garra, A. (2011). The role of IL-10 in immune regulation during M. tuberculosis infection. Mucosal Immunol. *4*, 261–270. https://doi.org/10.1038/mi.2011.7.

75. Wong, E.A., Evans, S., Kraus, C.R., Engelman, K.D., Maiello, P., Flores, W.J., Cadena, A.M., Klein, E., Thomas, K., White, A.G., et al. (2020). IL-10 Impairs Local Immune Response in Lung Granulomas and Lymph Nodes during Early *Mycobacterium tuberculosis* Infection. J. Immunol. *204*, 644–659. https://doi.org/10.4049/jimmunol.1901211.

76. Gideon, H.P., Phuah, J., Myers, A.J., Bryson, B.D., Rodgers, M.A., Coleman, M.T., Maiello, P., Rutledge, T., Marino, S., Fortune, S.M., et al. (2015). Variability in Tuberculosis Granuloma T Cell Responses Exists, but a Balance of Pro- and Anti-inflammatory Cytokines Is Associated with Sterilization. PLoS Pathog. *11*, e1004603. https://doi.org/10.1371/journal.ppat.1004603.

77. Jonsson, A.H., Zhang, F., Dunlap, G., Gomez-Rivas, E., Watts, G.F.M., Faust, H.J., Rupani, K.V., Mears, J.R., Meednu, N., Wang, R., et al. (2022). Granzyme K^+ CD8 T cells form a core population in inflamed

CellPress
OPEN ACCESS

human tissue. Sci. Transl. Med. *14*, eabo0686. https://doi.org/10.1126/scitranslmed.abo0686.

78. Thomas, M.F., Slowikowski, K., Manakongtreecheep, K., Sen, P., Tantivit, J., Nasrallah, M., Smith, N.P., Ramesh, S., Zubiri, L., Tirard, A., et al. (2021). Altered interactions between circulating and tissue-resident CD8 T cells with the colonic mucosa define colitis associated with immune checkpoint inhibitors. Preprint at bioRxiv. https://doi.org/10.1101/2021.09.17.460868.

79. Cai, Y., Wang, Y., Shi, C., Dai, Y., Li, F., Xu, Y., Zhang, P., Kong, F., Deng, G., Wen, Z., et al. (2022). Single-cell immune profiling reveals functional diversity of T cells in tuberculous pleural effusion. J. Exp. Med. *219*, e20211777. https://doi.org/10.1084/jem.20211777.

80. Kessler, B., Rinchai, D., Kewcharoenwong, C., Nithichanon, A., Biggart, R., Hawrylowicz, C.M., Bancroft, G.J., and Lertmemongkolchai, G. (2017). Interleukin 10 inhibits pro-inflammatory cytokine responses and killing of Burkholderia pseudomallei. Sci. Rep. *7*, 42791. https://doi.org/10.1038/srep42791.

81. O'Leary, S., O'Sullivan, M.P., and Keane, J. (2011). IL-10 Blocks Phagosome Maturation in *Mycobacterium tuberculosis*–Infected Human Macrophages. Am. J. Respir. Cell Mol. Biol. *45*, 172–180. https://doi.org/10.1165/rcmb.2010-0319OC.

82. de Waal Malefy, R., Haanen, J., Spits, H., Koncarolo, M.G., te Velde, A., Figdor, C., Johnson, K., Kastelein, R., Ysse, H., and de Vries, J.E. (1991). Interleukin 10 (IL-10) and Viral IL-10 Strongly Reduce Antigen-specific Human T Cell Proliferation by Diminishing the Antigen-presenting Capacity of Monocytes via downregulation of Class II Major Histocompatibility Complex Expression. J. Exp. Med. *174*, 915–924.

83. Cilfone, N.A., Ford, C.B., Marino, S., Mattila, J.T., Gideon, H.P., Flynn, J.L., Kirschner, D.E., and Linderman, J.J. (2015). Computational Modeling Predicts IL-10 Control of Lesion Sterilization by Balancing Early Host Immunity-Mediated Antimicrobial Responses with Caseation during *Mycobacterium tuberculosis* Infection. J. Immunol. *194*, 664–677. https://doi.org/10.4049/jimmunol.1400734.

84. Gazzinelli, R.T., Wysocka, M., Hieny, S., Scharton-Kersten, T., Cheever, A., Kühn, R., Müller, W., Trinchieri, G., and Sher, A. (1996). In the absence of endogenous IL-10, mice acutely infected with Toxoplasma gondii succumb to a lethal immune response dependent on CD4+ T cells and accompanied by overproduction of IL-12, IFN-gamma and TNF-alpha. J. Immunol. *157*, 798–805. https://doi.org/10.4049/jimmunol.157.2.798.

85. Sun, J., Madan, R., Karp, C.L., and Braciale, T.J. (2009). Effector T cells control lung inflammation during acute influenza virus infection by producing IL-10. Nat. Med. *15*, 277–284. https://doi.org/10.1038/nm.1929.

86. Cohen, S.B., Gern, B.H., Delahaye, J.L., Adams, K.N., Plumlee, C.R., Winkler, J.K., Sherman, D.R., Gerner, M.Y., and Urdahl, K.B. (2018). Alveolar Macrophages Provide an Early Mycobacterium tuberculosis Niche and Initiate Dissemination. Cell Host Microbe *24*, 439–446.e4. https://doi.org/10.1016/j.chom.2018.08.001.

87. Huang, L., Nazarova, E.V., Tan, S., Liu, Y., and Russell, D.G. (2018). Growth of *Mycobacterium tuberculosis* in vivo segregates with host macrophage metabolism and ontogeny. J. Exp. Med. *215*, 1135–1152. https://doi.org/10.1084/jem.20172020.

88. Gibbings, S.L., Thomas, S.M., Atif, S.M., McCubbrey, A.L., Desch, A.N., Danhorn, T., Leach, S.M., Bratton, D.L., Henson, P.M., Janssen, W.J., et al. (2017). Three Unique Interstitial Macrophages in the Murine Lung at Steady State. Am. J. Respir. Cell Mol. Biol. *57*, 66–76. https://doi.org/10.1165/rcmb.2016-0361OC.

89. Cai, S., Batra, S., Lira, S.A., Kolls, J.K., and Jeyaseelan, S. (2010). CXCL1 Regulates Pulmonary Host Defense to Klebsiella Infection via CXCL2, CXCL5, NF-κB, and MAPKs. J. Immunol. *185*, 6214–6225. https://doi.org/10.4049/jimmunol.0903843.

90. Phillipson, M., and Kubes, P. (2011). The neutrophil in vascular inflammation. Nat. Med. *17*, 1381–1390. https://doi.org/10.1038/nm.2514.

91. Esaulova, E., Das, S., Singh, D.K., Choreño-Parra, J.A., Swain, A., Arthur, L., Rangel-Moreno, J., Ahmed, M., Singh, B., Gupta, A., et al. (2021). The immune landscape in tuberculosis reveals populations linked to disease and latency. Cell Host Microbe *29*, 165–178.e8. https://doi.org/10.1016/j.chom.2020.11.013.

92. Pisu, D., Huang, L., Narang, V., Theriault, M., Lê-Bury, G., Lee, B., Lakudzala, A.E., Mzinza, D.T., Mhango, D.V., Mitini-Nkhoma, S.C., et al. (2021). Single cell analysis of *M. tuberculosis* phenotype and macrophage lineages in the infected lung. J. Exp. Med. *218*, e20210615. https://doi.org/10.1084/jem.20210615.

93. Lin, P.L., Pawar, S., Myers, A., Pegu, A., Fuhrman, C., Reinhart, T.A., Capuano, S.V., Klein, E., and Flynn, J.L. (2006). Early Events in *Mycobacterium tuberculosis* Infection in Cynomolgus Macaques. Infect. Immun. *74*, 3790–3803. https://doi.org/10.1128/IAI.00064-06.

94. Mehra, S., Pahar, B., Dutta, N.K., Conerly, C.N., Philippi-Falkenstein, K., Alvarez, X., and Kaushal, D. (2010). Transcriptional Reprogramming in Nonhuman Primate (Rhesus Macaque) Tuberculosis Granulomas. PLoS One *5*, e12266. https://doi.org/10.1371/journal.pone.0012266.

95. Miyamoto, M., Prause, O., Sjöstrand, M., Laan, M., Lötvall, J., and Lindén, A. (2003). Endogenous IL-17 as a Mediator of Neutrophil Recruitment Caused by Endotoxin Exposure in Mouse Airways. J. Immunol. *170*, 4665–4672. https://doi.org/10.4049/jimmunol.170.9.4665.

96. Pelletier, M., Maggi, L., Micheletti, A., Lazzeri, E., Tamassia, N., Costantini, C., Cosmi, L., Lunardi, C., Annunziato, F., Romagnani, S., et al. (2010). Evidence for a cross-talk between human neutrophils and Th17 cells. Blood *115*, 335–343. https://doi.org/10.1182/blood-2009-04-216085.

97. Bhattacharyya, C., Majumder, P.P., and Pandit, B. (2018). CXCL10 is overexpressed in active tuberculosis patients compared to M. tuberculosis-exposed household contacts. Tuberculosis (Edinb) *109*, 8–16. https://doi.org/10.1016/j.tube.2018.01.005.

98. Kumar, N.P., Hissar, S., Thiruvengadam, K., Banurekha, V.V., Balaji, S., Elilarasi, S., Gomathi, N.S., Ganesh, J., Aravind, M.A., Baskaran, D., et al. (2021). Plasma chemokines as immune biomarkers for diagnosis of pediatric tuberculosis. BMC Infect. Dis. *21*, 1055. https://doi.org/10.1186/s12879-021-06749-6.

99. Lee, K., Chung, W., Jung, Y., Kim, Y., Park, J., Sheen, S., and Park, K. (2015). CXCR3 ligands as clinical markers for pulmonary tuberculosis. Int. J. Tuberc. Lung Dis. *19*, 191–199. https://doi.org/10.5588/ijtld.14.0525.

100. Nonghanphithak, D., Reechaipichitkul, W., Namwat, W., Naranbhai, V., and Faksri, K. (2017). Chemokines additional to IFN-γ can be used to differentiate among Mycobacterium tuberculosis infection possibilities and provide evidence of an early clearance phenotype. Tuberculosis (Edinb) *105*, 28–34. https://doi.org/10.1016/j.tube.2017.04.005.

101. Ruhwald, M., Bjerregaard-Andersen, M., Rabna, P., Kofoed, K., Eugen-Olsen, J., and Ravn, P. (2007). CXCL10/IP-10 release is induced by incubation of whole blood from tuberculosis patients with ESAT-6, CFP10 and TB7.7. Microbes Infect. *9*, 806–812. https://doi.org/10.1016/j.micinf.2007.02.021.

102. Joosten, S.A., Van Meijgaarden, K.E., Arend, S.M., Prins, C., Oftung, F., Korsvold, G.E., Kik, S.V., Arts, R.J.W., Van Crevel, R., Netea, M.G., et al. (2018). Mycobacterial growth inhibition is associated with trained innate immunity. J. Clin. Invest. *128*, 1837–1851. https://doi.org/10.1172/JCI97508.

103. Jin, S., Guerrero-Juarez, C.F., Zhang, L., Chang, I., Ramos, R., Kuan, C.-H., Myung, P., Plikus, M.V., and Nie, Q. (2021). Inference and analysis of cell-cell communication using CellChat. Nat. Commun. *12*, 1088. https://doi.org/10.1038/s41467-021-21246-9.

104. Efremova, M., Vento-Tormo, M., Teichmann, S.A., and Vento-Tormo, R. (2020). CellPhoneDB: inferring cell–cell communication from combined expression of multi-subunit ligand–receptor complexes. Nat. Protoc. *15*, 1484–1506. https://doi.org/10.1038/s41596-020-0292-x.

105. Dimitrov, D., Türei, D., Garrido-Rodriguez, M., Burmedi, P.L., Nagai, J.S., Boys, C., Ramirez Flores, R.O., Kim, H., Szalai, B., Costa, I.G., et al. (2022). Comparison of methods and resources for cell-cell

communication inference from single-cell RNA-Seq data. Nat. Commun. *13*, 3224. https://doi.org/10.1038/s41467-022-30755-0.

106. Wolf, F.A., Angerer, P., and Theis, F.J. (2018). SCANPY: large-scale single-cell gene expression data analysis. Genome Biol. *19*, 15. https://doi.org/10.1186/s13059-017-1382-0.

107. Fang, Z., Liu, X., and Peltz, G. (2023). GSEApy: a comprehensive package for performing gene set enrichment analysis in Python. Bioinformatics *39*, btac757. https://doi.org/10.1093/bioinformatics/btac757.

108. Fleming, S.J., Chaffin, M.D., Arduini, A., Akkad, A.-D., Banks, E., Marioni, J.C., Philippakis, A.A., Ellinor, P.T., and Babadi, M. (2023). Unsupervised removal of systematic background noise from droplet-based single-cell experiments using CellBender. Nat. Methods *20*, 1323–1335. https://doi.org/10.1038/s41592-023-01943-7.

109. Badia-i-Mompel, P., Vélez Santiago, J., Braunger, J., Geiss, C., Dimitrov, D., Müller-Dott, S., Taus, P., Dugourd, A., Holland, C.H., Ramirez Flores, R.O., et al. (2022). decoupleR: ensemble of computational methods to infer biological activities from omics data. Bioinform. Adv. *2*, vbac016. https://doi.org/10.1093/bioadv/vbac016.

110. Muzellec, B., Teleńczuk, M., Cabeli, V., and Andreux, M. (2023). PyDESeq2: a python package for bulk RNA-seq differential expression analysis. Bioinformatics *39*, btad547. https://doi.org/10.1093/bioinformatics/btad547.

111. Capuano, S.V., Croix, D.A., Pawar, S., Zinovik, A., Myers, A., Lin, P.L., Bissel, S., Fuhrman, C., Klein, E., and Flynn, J.L. (2003). Experimental *Mycobacterium tuberculosis* Infection of Cynomolgus Macaques Closely Resembles the Various Manifestations of Human *M. tuberculosis* Infection. Infect. Immun. *71*, 5831–5844. https://doi.org/10.1128/IAI.71.10.5831-5844.2003.

112. Lin, P.L., Rodgers, M., Smith, L., Bigbee, M., Myers, A., Bigbee, C., Chiosea, I., Capuano, S.V., Fuhrman, C., Klein, E., et al. (2009). Quantitative Comparison of Active and Latent Tuberculosis in the Cynomolgus Macaque Model. Infect. Immun. *77*, 4631–4642. https://doi.org/10.1128/IAI.00592-09.

113. White, A.G., Maiello, P., Coleman, M.T., Tomko, J.A., Frye, L.J., Scanga, C.A., Lin, P.L., and Flynn, J.L. (2017). Analysis of ^{18}FDG PET/CT Imaging as a Tool for Studying Mycobacterium tuberculosis Infection and Treatment in Non-human Primates. J. Vis. Exp. 56375 Published online September 5, 2017. https://doi.org/10.3791/56375.

114. Lin, P.L., Dartois, V., Johnston, P.J., Janssen, C., Via, L., Goodwin, M.B., Klein, E., Barry, C.E., and Flynn, J.L. (2012). Metronidazole prevents reactivation of latent *Mycobacterium tuberculosis* infection in macaques. Proc. Natl. Acad. Sci. USA *109*, 14188–14193. https://doi.org/10.1073/pnas.1121497109.

115. Gierahn, T.M., Wadsworth, M.H., Hughes, T.K., Bryson, B.D., Butler, A., Satija, R., Fortune, S., Love, J.C., and Shalek, A.K. (2017). Seq-Well: portable, low-cost RNA sequencing of single cells at high throughput. Nat. Methods *14*, 395–398. https://doi.org/10.1038/nmeth.4179.

116. Macosko, E.Z., Basu, A., Satija, R., Nemesh, J., Shekhar, K., Goldman, M., Tirosh, I., Bialas, A.R., Kamitaki, N., Martersteck, E.M., et al. (2015). Highly Parallel Genome-wide Expression Profiling of Individual Cells Using Nanoliter Droplets. Cell *161*, 1202–1214. https://doi.org/10.1016/j.cell.2015.05.002.

117. Love, M.I., Huber, W., and Anders, S. (2014). Moderated estimation of fold change and dispersion for RNA-seq data with DESeq2. Genome Biol. *15*, 550. https://doi.org/10.1186/s13059-014-0550-8.

CellPress
OPEN ACCESS

STAR★METHODS

KEY RESOURCES TABLE

REAGENT or RESOURCE	SOURCE	IDENTIFIER
Antibodies		
Mouse anti-human CD3, clone SP34-2	BD Biosciences	N/A
Mouse anti-human CD4, clone L200	BD Biosciences	N/A
Rabbit anti-human CD20, polyclonal	ThermoFisher	N/A
Bacterial and virus strains		
Mycobacterium Tuberculosis: Erdman strain	Flynn Lab	N/A
Biological samples		
Cynomolgus macaque granulomas	This study	N/A
Chemicals, peptides, and recombinant proteins		
2-Mercaptoethanol	Sigma	Cat# M3148-25ML
RLT Buffer	QIAGEN	Cat#79216
dNTP	New England BioLabs	Cat#N0447L
RNase Inhibitor	Fisher Scientific	Cat#AM2696
Maxima RNaseH-minus RT Enzyme	Fisher Scientific	Cat#EP0753
AMPure RNAClean XP RNA-SPRI beads	Beckman Coulter	Cat#A63987
AMPure XP SPRI beads	Beckman Coulter	Cat#A63881
Guanidinium thiocynate	Sigma	Cat#AM9422
Sarkosyl	Sigma	Cat#L7414
Exonuclease I	New England BioLabs	Cat#M0293S
Klenow Fragment	New England BioLabs	Cat# M0212L
Accutase	Sigma	Cat#A6964
Dithiothreitol (DTT)	Sigma	Cat#43816
Polycarbonate membrane filters 62x22	Fisher Scientific/Sterlitech Corporation	Cat#NC0927472
Critical commercial assays		
Nextera XT DNA Library Prepartation Kit	Illumina	Cat#FC-131-1096
Kapa HiFi HotStart ReadyMix	Kapa Biosystems	Cat#KK2602
MACOSKO-2011-10 mRNA Capture Beads	ChemGenes	Cat#NC0927472
High Sensitivity D5000 ScreenTape	Agilent	Cat#5067-5592
Qubit dsDNA High-Sensitivity kit	Thermo Fisher	Cat#Q32854
Deposited data		
Processed and raw scRNA-seq data from primary infection, reinfection, and CD4-depletion animals	This study	Broad Single Cell Portal: https://singlecell.broadinstitute.org/single_cell/study/SCP2689/immunomodulatory-re-wiring-of-granuloma-cellularity-and-regulatory-networks-by-cd4-t-cells-following-mtb-reinfection#study-summary; SRA: PRJNA900256
Experimental models: Organisms/strains		
Cynomolgus macaques	Valley Biosystems	N/A
Oligonucleotides		
Seq-Well ISPCR: AAG CAG TGG TAT CAA CGC AGA GT	Integrated DNA Technologies	N/A
Custom Read 1 Primer: GCC TGT CCG CGG AAG CAG TGG TAT CAA CGC AGA GTA C	Integrated DNA Technologies	N/A

(*Continued on next page*)

Continued		
REAGENT or RESOURCE	SOURCE	IDENTIFIER
Seq-Well 5' TSO: AAG CAG TGG TAT CAA CGC AGA GTG AAT rGrGrG	Integrated DNA Technologies	N/A
Seq-Well Custom P5-SMART PCR hybrid: AAT GAT ACG GCG ACC ACC GAG ATC TAC ACG CCT GTC CGC GGA AGC AGT GGT ATC AAC GCA GAG TAC	Integrated DNA Technologies	N/A
Seq-Well dN-SMRT oligo: AAG CAG TGG TAT CAA CGC AGA GTG ANN NGG NNN B	Integrated DNA Technologies	N/A
Software and algorithms		
Python package – Scanpy v1.9.5	Wolf et al.[106]	https://github.com/scverse/scanpy
Python package – gseapy	Fang et al.[107]	https://gseapy.readthedocs.io/en/latest/introduction.html
R package – multinichenet	Browaeys et al.[56]	https://github.com/saeyslab/multinichenetr
R package – Seurat v4.1.1	Github	https://github.com/satijalab/seurat
CellBender	Fleming et al.[108]	https://github.com/broadinstitute/CellBender
Source code	This study	https://github.com/bromleyjd/scRNA_Reinfection_CD4_Tcell_depletion https://zenodo.org/records/11389877
R package – DESeq2 v1.30	Bioconductor	https://bioconductor.org/packages/release/bioc/html/DESeq2
Prism	GraphPad Software	https://www.graphpad.com/scientific-software/prism/E/
Python package – decoupleR	Badia-i-Mompel et al.[109]	https://decoupler-py.readthedocs.io/en/latest/notebooks/usage.html
Python package – PyDESeq2	Muzellec et al.[110]	https://github.com/owkin/PyDESeq2

EXPERIMENTAL MODEL AND STUDY PARTICIPANT DETAILS

Research animals

All experimental manipulations, protocols, and care of the animals were approved by the University of Pittsburgh School of Medicine Institutional Animal Care and Use Committee (IACUC). The protocol assurance number for our IACUC is A3187-01. Our specific protocol approval number for this project is 15066174. The IACUC adheres to national guidelines established in the Animal Welfare Act (7 U.S.C. Sections 2131–2159) and the Guide for the Care and Use of Laboratory Animals (8th Edition) as mandated by the U.S. Public Health Service Policy.

All macaques used in this study were housed at the University of Pittsburgh in rooms with autonomously controlled temperature, humidity, and lighting. Animals were singly housed in caging at least 2 square meters apart that allowed visual and tactile contact with neighboring conspecifics. The macaques were fed twice daily with biscuits formulated for nonhuman primates, supplemented at least 4 days/week with large pieces of fresh fruits or vegetables. Animals had access to water *ad libitum*. Because our macaques were singly housed due to the infectious nature of these studies, an enhanced enrichment plan was designed and overseen by our nonhuman primate enrichment specialist. This plan has 3 components. First, species-specific behaviors are encouraged. All animals have access to toys and other manipulata, some of which will be filled with food treats (e.g., frozen fruit, peanut butter, etc.). These are rotated on a regular basis. Puzzle feeders foraging boards, and cardboard tubes containing small food items also are placed in the cage to stimulate foraging behaviors. Adjustable mirrors accessible to the animals stimulate interaction between animals. Second, routine interaction between humans and macaques are encouraged. These interactions occur daily and consist mainly of small food objects offered as enrichment and adhere to established safety protocols. Animal caretakers are encouraged to interact with the animals (by talking or with facial expressions) while performing tasks in the housing area. Routine procedures (e.g., feeding, cage cleaning, etc) are done on a strict schedule to allow the animals to acclimate to a routine daily schedule. Third, all macaques are provided with a variety of visual and auditory stimulation. Housing areas contain either radios or TV/video equipment that play cartoons or other formats designed for children for at least 3 hours each day. The videos and radios are rotated between animal rooms so that the same enrichment is not played repetitively for the same group of animals.

All animals are checked at least twice daily to assess appetite, attitude, activity level, hydration status, etc. Following *M. tuberculosis* infection, the animals are monitored closely for evidence of disease (e.g., anorexia, weight loss, tachypnea, dyspnea, coughing). Physical exams, including weights, are performed on a regular basis. Animals are sedated prior to all veterinary procedures (e.g., blood draws, etc.) using ketamine or other approved drugs. Regular PET CT imaging is conducted on most of our

macaques following infection and has proved very useful for monitoring disease progression. Our veterinary technicians monitor animals especially closely for any signs of pain or distress. If any are noted, appropriate supportive care (e.g. dietary supplementation, rehydration) and clinical treatments (analgesics) are given. Any animal considered to have advanced disease or intractable pain or distress from any cause is sedated with ketamine and then humanely euthanatized using sodium pentobarbital.

METHOD DETAILS

Animals, infections, CD4 depletion, and disease tracking by PET CT

Nineteen male cynomolgus macaques (*Macaca fasicularis*) with age range of 5.1-8.4 years were obtained from Valley Biosystems (Sacramento, California). Obtaining female adult macaques is difficult due to animal shortages and reserving females for breeding. Animals were placed in quarantine for 1 month where they were monitored to ensure good physical health and no prior *Mtb* infection. All animal data are in Table S1. All animals were infected with Library P DNA-tagged *Mtb* Erdman via bronchoscopic instillation as previously described.[111,112] Thirteen macaques received *Mtb* library P as the first infection. Granuloma formation, lung inflammation and overall disease were tracked using ^{18}F-fluorodeoxyglucose (FDG) PET-CT every 4 weeks throughout infection. PET-CT scans were analyzed using OsiriX viewer as previously described with a detection limit of 1mm.[113] The first infection was followed for 9 weeks before drug-treating all 13 macaques. Based on our previous study,[16] exacerbation of TB disease occurs after CD4$^+$ T cell depletion, thus to facilitate identification of new granulomas arising from the second infection we opted to treat all macaques with anti-TB drugs. Macaques were given anti-TB drugs orally once daily for 4-5 months (RIF 20mg/kg; INH 15mg/kg; ETH 50mg/kg; PZA 150mg/kg, HRZE).[114] Compliance ranged from 97-100%. Our previous data support that drug treatment of primary infection reduces but does not abolish protection against reinfection.[9] The 13 macaques were matched by PET CT for disease status and randomized into 2 cohorts: CD4$^+$ T cell depletion (n=7) and IgG control (n=6). After resting for 4 weeks after drug treatment, CD4R1 (50mg/kg, NHP Reagent Resource), a rhesus recombinant CD4$^+$ T cell-depleting antibody, was administered subcutaneously in 4 animals and intravenously in 3 animals 1 week before the second infection with *Mtb* Library S and then was administered intravenously every 2 weeks until necropsy. CD4$^+$ T cell depletion was monitored by flow cytometry in the blood and complete blood count weekly. To measure CD4$^+$ T cell depletion in tissues, a peripheral lymph node biopsy was performed before CD4$^+$ T cell depletion and the CD4$^+$ T cell frequency was compared with a peripheral lymph node from the same macaque obtained at necropsy. Macaques from the IgG control group received rhesus recombinant IgG1 control antibody (50mg/kg, NHP Reagent Resource) following the same timeline of the CD4$^+$ T cell-depletion group. Six macaques were included as naïve controls infected with *Mtb* Library S only. Macaques received 4-12 CFU of *Mtb* Library P for the first infection and 8-22 CFU of *Mtb* Library S for the second infection (or the first infection for the naïve monkeys). Dose was calculated from colony counts after plating an aliquot of the infection inoculum on 7H11 agar plates and incubating for 3 weeks at 37°C/5% CO_2.

Necropsy procedures

Procedures done during necropsy have been previously described. Briefly, 1-3 days prior to necropsy, a PET CT scan was taken and used to identify the location and metabolic activity (FDG avidity) of granulomas and lymph nodes; this scan was used as a map to aid in the individual identification and excision of these samples during necropsy. On the day of necropsy, macaques were humanely sacrificed with sodium pentobarbital and terminally bled. Individual granulomas, thoracic and peripheral lymph nodes, lung tissue, spleen and liver were all excised and homogenized separately into single cell suspensions. New granulomas determined by PET-CT and uninvolved lung lobes (no granuloma present in the lobe) were enzymatically homogenized using a human tumor dissociation kit (Miltenyi Biotec) and a gentleMACS Dissociator (MiltenyiBiotec) following manufacturer's protocols. Homogenates were aliquoted for plating on 7H11 agar for bacterial burden, freezing for DNA extraction and staining for flow cytometry analysis. Any remaining samples were frozen for future use. Homogenates were plated in serial dilutions on 7H11 medium and incubated at 37°C/5% CO_2 for 3 weeks before enumeration of CFU.

Isolation of genomic DNA from bacteria

DNA extraction was performed on granuloma and lymph node homogenates, as well as their scrapates (scraped colonies that grew on 7H11 agar plates) for library identification as described previously.[19] Briefly, a small aliquot of the homogenate or scrapate were vortexed with 0.1mm zirconia-silica beads (BioSpec Products, Inc.) and subsequently extracted twice with phenol chloroform isoamyl alcohol (25:24:1, Sigma-Aldrich) before precipitating DNA with molecular grade 100% isopropanol (Sigma-Aldrich) and 3M sodium acetate (Sigma-Aldrich) and resuspending in nuclease-free water (Invitrogen).

Library identification

Identification of library DNA tags have been previously described.[8] Briefly, DNA was amplified by PCR for 24-36 cycles before using in the NanoString nCounter assay (NanoString Technologies) with custom designed probes.[25] New granulomas after reinfection were identified by PET-CT scan comparing pre- and post-reinfection scans and verified by presence of Library S barcodes.

Flow cytometry

Cells were stained with a viability marker (LIVE/DEAD fixable blue dead cell stain kit, Invitrogen) and surface markers which include CD3 (clone SP34-2, BD Pharmingen), CD4 (Clone L200, BD Horizon), CD8 (Clone SK1, BD Biosciences) and CD20 (Clone 2H7, eBioscience).

Antibody validation

To test whether the anti-CD4+ depletion antibody masks CD4 receptors, peripheral blood mononuclear cells (PBMC) were incubated with 1X (0.77 mg/ml, the calculated concentration of αCD4 antibodies in blood of macaques given a dose of 50mg/kg), 0.25X and 4X concentration of CD4 T cell-depleting antibody for 30 minutes at 37°C before surface staining with CD3 (clone SP34-2, BD Pharmingen), CD4 (Clone L200, BD Horizon) and CD8 (Clone RPA-T8, BD Biosciences) surface markers. PBMCs that were not incubated with the αCD4 antibody were included as a control. Data was acquired using the LSR II (BD) and analyzed using FlowJo software v10.6.1 (BD).

Single-cell RNA-sequencing (scRNA-seq) and alignment

Massively parallel scRNA-seq was performed using the Seq-Well S^3 platform, as previously described.[27,115] Approximately 15,000-20,000 cells were loaded onto Seq-Well arrays equipped with uniquely barcoded mRNA capture beads (ChemGenes). Cells were allowed to settle by gravity into wells for 10 minutes, after which the arrays were washed with PBS and serum-free RPMI. Arrays were then sealed with a semi-permiable polycarbonate membrane and incubated at 37°C for 30 minutes. Cell lysis was achieved by incubating the sealed arrays in a lysis buffer (5 M guanidine thiocyanate, 10 mM EDTA, 0.1% BME, and 0.1% sarkosyl) for 20 minutes. Subsequently, the arrays were rocked in hybridization buffer (2M NaCl, 8% v/v PEG8000) for 40 minutes. After membrane removal, the arrays were washed with in Seq-Well wash buffer (2M NaCl, 3 mM MgCl2, 20 mM Tris-HCl, and 8% v/v PEG8000) to collect the mRNA capture beads. Reverse transcription was conducted at 52°C using Maxima H Minus Reverse Transcriptase (ThermoFisher), and excess primers were removed with an Exonuclease I digestion (New England Biolabs). Whole transcriptome amplification (WTA) was achieved via PCR using KAPA Hifi PCR Mastermix (Kapa Biosystems). The WTA product was purified using Agencourt Ampure beads (Beckman Coulter). For sequencing, dual-indexed 3' DGE libraries were prepared using Nextera XT (Illumina) and sequenced to depth on the NovaseqS4 platform with a paired-end read structure (R1: 20 bases; I1: 8 bases; I2: 8 bases; R2: 50 bases) using custom sequencing primers. Transcript reads were tagged for cell barcode and UMI utilizing DropSeqTools v1.12.[116] These tagged sequencing reads were subsequently aligned to the *Macaca fascicularis* v5 genome (https://useast.ensembl.org/Macaca_fascicularis/Info/Index) using the Dropseq-tools pipeline on the Terra platform (app.terra.bio). By collapsing the aligned reads based on barcode and UMI sequences, we derived digital gene expression matrices for each array, covering 10,000 barcodes.

QUANTIFICATION AND STATISTICAL ANALYSIS

Statistical analysis on macaque samples (depletion and CFU)

Test for normality was conducted with a Shapiro-Wilk test. For assessment of depletion over time, mixed effects model with Dunnett's multiple comparison test adjusted for the comparison of IgG vs anti-CD4 and IgG vs naïve. For pre- and post-PET data, two-way ANOVA with Bonferroni multiple comparisons test. For paired analyses, Wilcoxon matched-pairs signed rank tests were used. For comparison of three groups (IgG vs naïve and IgG vs αCD4), either one-way ANOVA with Dunnett's multiple comparisons or Kruskal-Wallis with Dunn's multiple comparisons were used dependent on normality.

Data processing and quality control

For individual granuloma, collapsed gene expression matrices containing 10,000 barcodes were subject to CellBender[108] to estimate the fraction of ambient RNA contaminating cell transcriptomes. The CellBender "remove-background" function was then applied using default parameters. Individual CellBender "corrected" matrices were then subject to Scrublet, using default parameters, to identify putative doublets. Transcriptomes with a doublet_score >0.30 were removed from downstream analyses. Sample-specific gene expression matrices were then combined and analyzed using Scanpy[106] (version 1.8.2). Transcriptomes were filtered using min_genes > 300, min_counts>500, mitochondrial_threshold=0.05, and genes expressed in fewer than 10 cells were removed. Gene expression counts were normalized using default Scanpy parameters (i.e., log_2(TP10K+1)).

Dimensionality reduction and batch correction

Following preliminary filtering processes, we performed coarse-level cell type clustering and iterative sub-clustering to annotate cell types and identify low-quality transcriptomes (e.g., doublets) not identified or removed during preliminary quality control processing, respectively. The top 2,000 highly-variable genes – identified using the Scanpy "highly_variable_genes function" – were used for dimensionality reduction. Following variable gene selection, these data were subject to scaling, principal component analysis (PCA), integration to mitigate sample-specific batch effects, and Leiden-based clustering. More specifically, data were scaled to 10, and the top 19 principal components (PCs) were used for dimensionality reduction. PCs were used to construct a neighborhood graph using the scanpy.pp.neighbors function, setting n_neighbors=40 and using the top 19 PCs. Leiden-based clustering was then implemented, setting the resolution= 1.0, which identified 26 distinct clusters.

Cell clustering and annotation

From these 26 clusters we identified 14 coarse cell types. The Leiden resolution=1.0 failed to distinguish between several cell types (e.g., cDCs 1 and pDCs; alveolar type-1 and alveolar type 2 cells). As a result, these preliminary coarse-level cell types were not used as the final reference but instead used to inform sub-clustering. All coarse-level cell types (e.g., T, NK cells, macrophages) were

subject to sub-clustering to remove low-quality cells. Transcriptomes classified as doublets featured elevated expression of genes derived from distinct cell ontologies. These doublets were excluded from downstream analyses.

Following quality control processing, our data set comprised 88,360 high-quality transcriptomes, which were annotated as 15 distinct cell types, including: alveolar type-1 cells, alveolar type 2 cells, B cells, ciliated cells, endothelial cells, eosinophils, fibroblasts, macrophages, mast cells, neutrophils, plasma cells, T, NK cells, cDCs 1, cDCs 2, and pDCs. Among cDCs 1, cDCs 2, pDCs, and plasma cells additionally subset diversity was not found, as such these coarse-level annotations are equivalent to cellular subsets. The major cell populations alveolar type-1 cells, alveolar type 2 cells, B cells, ciliated cells, endothelial cells, eosinophils, fibroblasts, macrophages, mast cells, neutrophils, and T, NK cells underwent further sub-clustering to discern cellular subtypes. Sub-clustering resolution was determined by selecting the most stable/robust silhouette score that uncovered biologically relevant and/or known cell subsets (e.g., Tregs). As paired protein expression (i.e., CITE-seq) data was unavailable NK-like cells were annotated as "NK-like" due to low-level expression of *CD3D*, and elevated expression of genes canonically associated with an NK cell transcriptome.

Differential abundance analysis of scRNA-seq cell type and subset frequencies

To identify differential cell type frequencies across naïve, IgG, and αCD4 granuloma, we implemented three distinct statistical frameworks: (1) scCODA,[29] (2) the Mann-Whitney U-test, and (3) Fischer's exact test.

One inherent challenge in scRNA-seq data is the compositional nature of cell proportions – they are not mutually exclusive. Illustratively, the elevation of one cell subset's proportion inherently diminishes the proportions of others due to the requirement that all proportions sum to one (e.g., antibody-mediated $CD4^+$ T cell depletion results in elevated frequencies of $CD8A^+$ T cells among T, NK cells). To address these limitations, we implemented scCODA, a statistical framework rooted in a Bayesian hierarchical model, which is adept at dissecting cell type co-dependencies and the low inputs typically associated with scRNA-seq data, thus ensuring that observed shifts in cell type or subset frequencies are biologically significant. In addition to scCODA, we employed the Mann-Whitney U-test and Fischer's exact test. Differentially abundant cell types and subset had to be identified as significant by at least two of the aforementioned methods.[29,30]

Differential expression analysis

Pairwise (i.e., naïve vs IgG; αCD4 vs IgG) differential expression (DE) analyses were conducted using MAST, on $\log_2$(TP10K+1) normalized gene expression data[38,39] (Figures 4, 5, and 6). The covariates mitochondrial reads and number of genes we included when performing DE.

Pseudobulk differential expression analysis

To robustly identify DE genes among cell subsets, we performed pseudobulk DE analysis. For cell subsets of interest, we generated pseudobulk counts from scRNA-seq gene expression matrices. Psuedobulk counts and associated metadata (e.g., sample, NHP identity) were imported into R and subject to DE analysis using the DESeq2 package.[117] DE was performed using the Wald statistical test and highlighted genes where selected using the threshold $pvalue<0.05$ and $\log_2(|\text{fold change}|)>0.3785$.

For cell type-specific (e.g., T, NK cells) pseudobulk analyses the Wilcoxon rank sum statistical test was employed.

Pathway enrichment analysis

All monocyte derived transcriptomes were subject to Gene Set Enrichment Analysis (GSEA) and scored against the Hallmark gene sets to identify differentially activated pathways following *Mtb* reinfection (IgG vs naïve; IgG vs αCD4). This analysis was performed using GSEApy.[107]

We further sought to identify molecular pathways that were enriched among monocyte-derived subsets (one macrophage subset vs rest (i.e., all other macrophage populations) using GOMF gene sets and the python package decoupleR.[109]

Pathway RespOnsive GENes (PROGENy)[41] analyses were performed on T, NK cell subsets to identify how reinfection modulates changes in core regulatory programs. Pseudobulk matrices were created using Decoupler and used and input for DESeq2 (pydeseq2[110]; to identify differentially expressed genes (IgG vs naïve; IgG vs αCD4). These differentially expressed genes were used as input for performing PROGENy – this was implemented from the decoupleR[109] package.

Differential cell-cell and receptor-ligand analyses

To discern putative differential cell-cell interactions from our scRNA-seq dataset, we adopted MultiNicheNet.[56] Distinct from conventional interaction cell-cell interaction methods, MultiNicheNet can identify differential, context-dependent cellular communications, leveraging 'pseudobulk' profiles from scRNA-seq data.

Using MultiNicheNet, we assessed the interaction strength between cell types and identified putative differential cell-cell and ligand-receptor (L-R) pairs – derived from MultiNicheNet's 50 top-prioritized links (i.e., top 50 predictions across contrasts, senders, and receivers). To highlight highly interconnected cellular populations, we focused on the top 10 (five per experimental group) – as identified in MultiNicheNet's 50 top-prioritized links – differential interactions per experimental group. To identify cellular subsets underlying differential coarse-grain cell-cell L-R among reinfection granulomas (IgG vs naïve, and IgG vs αCD4), we removed all nonimmune cell subsets to identify putative "senders" and "receiver" subsets modulating immune tone in the reinfection granuloma. The top 50 prioritized links among all *IL10*+ T, NK cell sender subsets were queried to identify putative receivers (among all immune cell

subsets). The same strategy was employed in determining putative receivers of neutrophil- and monocyte-senders. Interaction matrices were visualized in Python.

Leveraging the top 200 *IL10-IL10RA* prioritized linkages we quantified the diversity (Shannon diversity) of putative intercellular signaling networks in IgG vs naïve granulomas. We additionally quantified the "In"-Degree centrality and "Out"-Degree centrality to identify the relative contribution of myeloid (all cellular subsets of myeloid lineage, e.g., mast cells, neutrophils) and T, NK cells.

Immunity, Volume *57*

Supplemental information

CD4$^+$ T cells re-wire granuloma cellularity and regulatory networks to promote immunomodulation following *Mtb* reinfection

Joshua D. Bromley, Sharie Keanne C. Ganchua, Sarah K. Nyquist, Pauline Maiello, Michael Chao, H. Jacob Borish, Mark Rodgers, Jaime Tomko, Kara Kracinovsky, Douaa Mugahid, Son Nguyen, Qianchang Dennis Wang, Jacob M. Rosenberg, Edwin C. Klein, Hannah P. Gideon, Roisin Floyd-O'Sullivan, Bonnie Berger, Charles A. Scanga, Philana Ling Lin, Sarah M. Fortune, Alex K. Shalek, and JoAnne L. Flynn

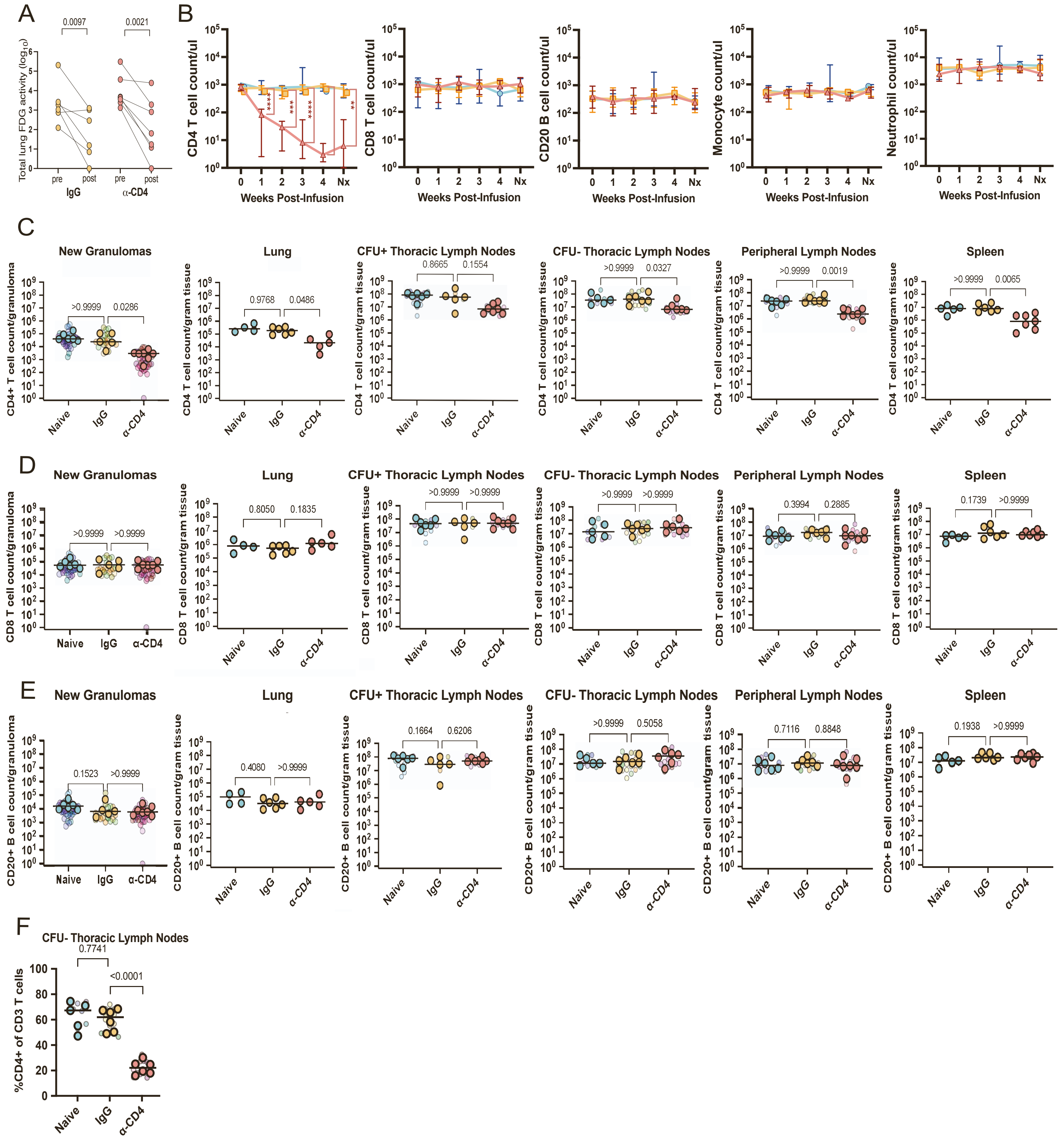

A
B
C
D
E
F
Total lung FDG activity (log10)
IgG
α-CD4
pre
post
0.0097
0.0021
CD4 T cell count/ul
CD8 T cell count/ul
CD20 B cell count/ul
Monocyte count/ul
Neutrophil count/ul
Weeks Post-Infusion
Nx
New Granulomas
Lung
CFU+ Thoracic Lymph Nodes
CFU- Thoracic Lymph Nodes
Peripheral Lymph Nodes
Spleen
CD4+ T cell count/granuloma
CD4 T cell count/gram tissue
CD8 T cell count/granuloma
CD8 T cell count/gram tissue
CD20+ B cell count/granuloma
CD20+ B cell count/gram tissue
Naive
IgG
α-CD4
%CD4+ of CD3 T cells

Figure S1. Cellular frequencies across anatomic compartments, related to Figure 1. (A) Total lung FDG activity pre- and post-HRZE drug treatment. Two-way ANOVA with Bonferroni's multiple comparisons test. Dots with connected lines represent individual animals. Number of animals: IgG n=6, αCD4 n=7. **(B)** Absolute counts (count/uL) of CD4$^+$ T cells, CD8$^+$ T cells, CD20$^+$ B cells, monocytes, and neutrophils post-antibody infusion in peripheral blood. Median and range shown; mixed-effects model with Dunnett's multiple comparisons test (** p<0.01, ***, p<0.001, **** p<0.0001). Number of animals: Naïve n=6, IgG n=6, αCD4 n=7. **(C)** Absolute counts (count/mL) of CD4$^+$ T cells in new granulomas (Animals: Naïve=6, IgG=5, αCD4 n=7; Granulomas: Naïve n=37, IgG n=21, αCD4 n=39), uninvolved lung (Animals: Naïve n=4, IgG n=6, αCD4 n=5), CFU$^+$ thoracic lymph nodes (Animals: Naïve n=6, IgG n=5, αCD4 n=7; LN: Naïve n=14, IgG n=6, αCD4 n=11), CFU$^-$ thoracic lymph nodes (Animals: Naïve n=5, IgG n=6, αCD4 n=6; LN: Naïve n=8, IgG n=17, αCD4 n=13), peripheral lymph nodes (Animals: Naïve n=6, IgG n=6, αCD4 n=7; LN: Naïve n=11, IgG n=12, αCD4 n=13), and spleen (Animals: Naïve n=5, IgG n=6, αCD4 n=7). **(D)** Absolute counts (count/mL) of CD8$^+$ T cells in new granulomas (Animals: Naïve n=6, IgG n=5, αCD4 n=7; Granulomas: Naïve n=37 IgG n=21, αCD4 n=39), uninvolved lung (Animals: Naïve n=4, IgG n=6, αCD4 n=5), CFU$^+$ thoracic lymph nodes (Animals: Naïve n=6, IgG n=5, αCD4 n=7; LN: Naïve n=14, IgG n=6, αCD4 n=11) and CFU$^-$ thoracic lymph nodes (Animals: Naïve n=5, IgG n=6, αCD4 n=6; LN: Naïve n=8, IgG n=17, αCD4 n=13), peripheral lymph nodes (Animals: Naïve n=6, IgG n=6, αCD4 n=7; LN: Naïve n=11, IgG n=12, αCD4 n=13), and spleen (Animals: Naïve n=5, IgG n=6, αCD4 n=7). **(E)** Absolute counts (count/mL) of CD20$^+$ B cells in new granulomas (Animals: Naïve n=6, IgG n=5, αCD4 n=7; Granulomas: Naïve n=37 IgG n=20, αCD4 n=39), uninvolved lung (Animals: Naïve n=4, IgG n=6, αCD4 n=5), CFU$^+$ thoracic lymph nodes (Animals: Naïve n=6, IgG n=5, αCD4 n=7; LN: Naïve n=14, IgG n=6, αCD4 n=11), CFU$^-$ thoracic lymph nodes (Animals: Naïve n=5, IgG n=6, αCD4 n=6; LN: Naïve n=8, IgG n=17, αCD4 n=13), peripheral lymph nodes (Animals: Naïve n=6, IgG n=6, αCD4 n=7; LN: Naïve n=11, IgG n=12, αCD4 n=13), and spleen (Animals: Naïve n=5, IgG n=6, αCD4 n=7). **(F)** Fraction of CD3$^+$, CD4$^+$ cells from CFU$^-$ thoracic lymph nodes resected at necropsy. (Animals: Naïve=5, IgG=6, αCD4 n=6; LN: Naïve=8, IgG=17, αCD4 n=13) One-way ANOVA with Dunnett's multiple comparisons test. (C-F) Transparent smaller dots represent granulomas or lymph nodes, colored by animal. Larger dots represent mean per animal and lines represent medians. Kruskal-Wallis with Dunn's multiple comparisons test adjusted p-values reported.

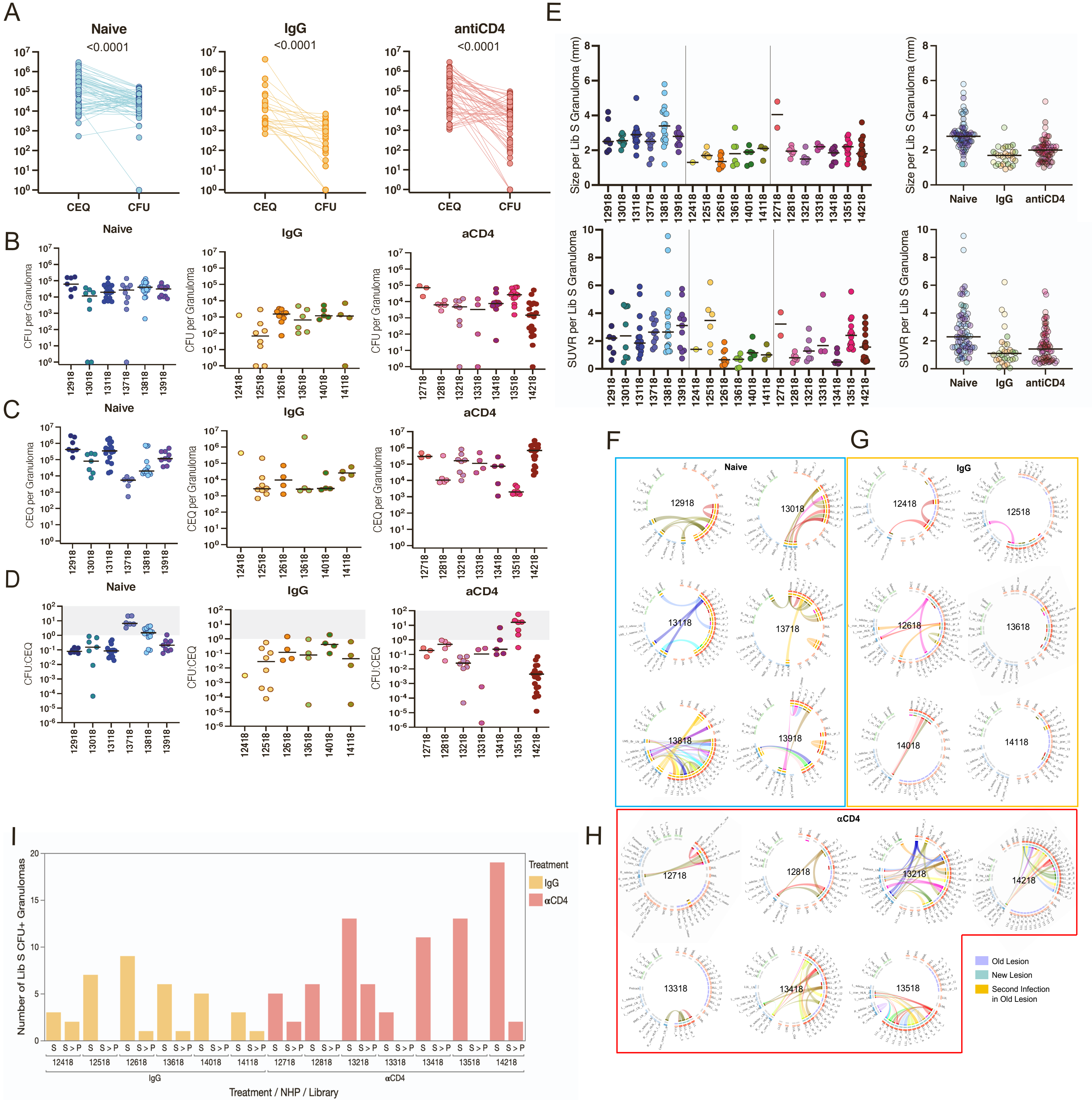
A
Naive
<0.0001
IgG
<0.0001
antiCD4
<0.0001
CEQ
CFU
B
Naive
IgG
aCD4
CFU per Granuloma
12918
13018
13118
13718
13818
13918
12418
12518
12618
13618
14018
14118
12718
12818
13218
13318
13418
13518
14218
C
CEQ per Granuloma
D
CFU:CEQ
E
Size per Lib S Granuloma (mm)
SUVR per Lib S Granuloma
Naive
IgG
antiCD4
F
Naive
G
IgG
H
αCD4
Old Lesion
New Lesion
Second Infection
in Old Lesion
I
Number of Lib S CFU+ Granulomas
Treatment
IgG
αCD4
S
S > P
Treatment / NHP / Library

Figure S2. *Mtb* CFU, CEQ, CFU:CEQ, and dissemination, related to Figure 2. (A) CEQ (left), and CFU (right). Individual dots represent individual TB granulomas. Linkages depict CEQ and CFU within the same lesion. Wilcoxon matched-pairs signed rank test. Number of granulomas: Naïve CEQ n=59, CFU n=70; IgG CEQ n=25, CFU n=34; αCD4 CEQ n=51, CFU n=65. **(B)** *Mtb* CFU per granuloma. Integers on the x-axis represent an individual macaque. Colored dots are individual granuloma. Lines represent median per animal. Granuloma numbers for animals (from left to right): Naïve n=(7, 8, 16, 10, 18, 11); IgG n=(1, 8, 10, 6, 5, 4); αCD4 n=(3, 6, 8, 4, 11, 14, 19). **(C)** *Mtb* CEQ per granuloma. Integers on the x-axis represent an individual macaque. Colored dots are individual granuloma. Granuloma numbers for animals (from left to right): Naïve n=(7, 7, 16, 6, 14, 9); IgG n=(1, 8, 4, 4, 4, 4); αCD4 n=(3, 5, 8, 4, 5, 7, 19). **(D)** CFU:CEQ per granuloma. Integers on the x-axis represent an individual macaque. Colored dots are individual granuloma. Lines represent median per animal. Granuloma numbers for animals (from left to right): Naïve n=(7, 7, 16, 6, 14, 9); IgG n=(1, 8, 4, 4, 4, 4); αCD4 n=(3, 5, 8, 4, 5, 7, 19). **(E)** Size in mm per library S granuloma (as measured on CT) by monkey and by treatment group. Granuloma numbers for animals (from left to right): Naïve n=70 (7, 8, 16, 10, 18, 11); IgG n=29 (1, 6, 8, 6, 5, 3); αCD4 n=61 (2, 6, 7, 4, 8, 14, 20). FDG Avidity (SUVR) per library S granuloma by monkey and by treatment group. Granuloma numbers for animals (from left to right): Naïve n=69 (7, 8, 16, 10, 17, 11); IgG n=29 (1, 6, 8, 6, 5, 3); αCD4 n=61 (2, 6, 7, 4, 8, 14, 20). Each symbol represents an individual granuloma symbols are colored by animal. Median lines are shown both within animal and treatment groups. (**F-H**) Circos plots depicting *Mtb* strains shared between anatomical sites in naïve (E), reinfected (F) or reinfected with CD4 depletion (G) animals. Individual circos plots represent a single macaque, labeled with their study ID. In the plots, each wedge represents a distinct tissue site that was sampled and/or sequenced for barcodes. There are three tracks in each plot. The outer ring defines tissue samples as being from lungs (red), thoracic lymph nodes (blue) or distal extrapulmonary sites (green). Lighter shades represent tissues that were plated for *Mtb* but were sterile. The middle ring represents lesions that were detected by PET-CT during primary infection with the first *Mtb* library ('old lesion'), new lesions detected by PET-CT after re-infection ('new lesion'), or new lesions (as defined by Library S

barcode sequencing) that were also found at sites where old lesions had previously formed (detected by PET-CT). The innermost ring represents distinct barcodes found in each tissue, where each unique barcoded *Mtb* strain (from the secondary *Mtb* library) is given a different color. Tissues that share the same *Mtb* strain by sequencing are linked by ribbons. Lung tissues are further grouped by lobe, abbreviated as follows: RUL, right upper lobe; RML, right middle lobe; RLL, right lower lobe; LUL, left upper lobe; LML, left middle lobe; LLL, left lower lobe; Acc, accessory lobe. (**I**) Bars represent either number of total CFU+ Library S granulomas ("S") or the number of the subset of library S found in old (established as library P) granulomas ("S > P") within each animal. Number of Library S in old granulomas ranged from 0 to 2 in IgG animals and 0 to 6 in CD4-depleted animals.

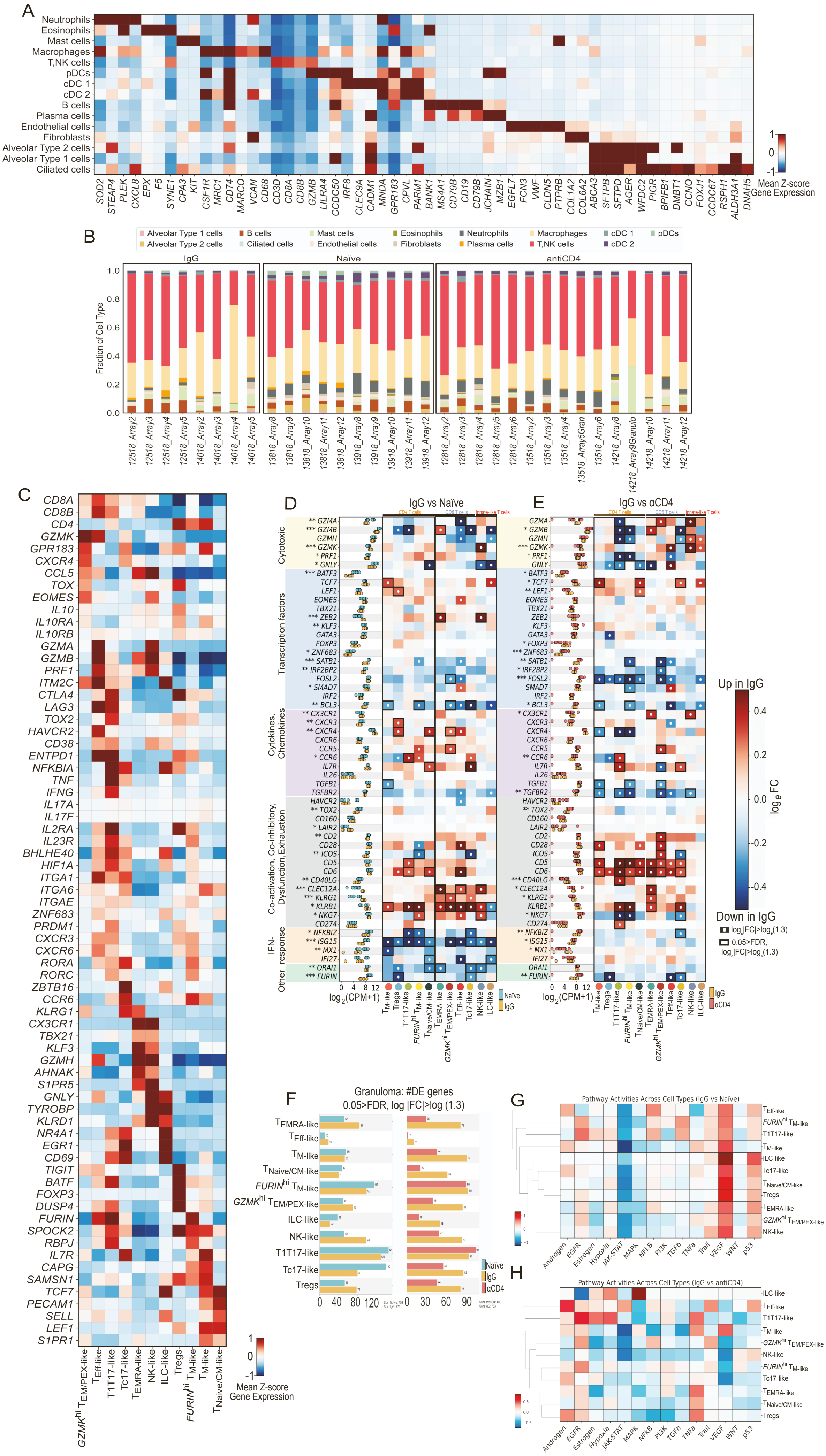
A
Neutrophils
Eosinophils
Mast cells
Macrophages
T,NK cells
pDCs
cDC 1
cDC 2
B cells
Plasma cells
Endothelial cells
Fibroblasts
Alveolar Type 2 cells
Alveolar Type 1 cells
Ciliated cells
SOD2 STEAP4 PLEK CXCL8 EPX F5 SYNE1 CPA3 KIT CSF1R MRC1 CD74 MARCO VCAN CD68 CD3D CD8A CD8B GZMB LILRA4 CCDC50 IRF8 CLEC9A CADM1 MNDA GPR183 CPVL PARM1 BANK1 MS4A1 CD79B CD19 CD79B JCHAIN MZB1 EGFL7 FCN3 VWF CLDN5 PTPRB COL1A2 COL6A2 ABCA3 SFTPB SFTPD AGER WFDC2 PIGR BPIFB1 DMBT1 CCNO FOXJ1 CCDC67 RSPH1 ALDH3A1 DNAH5
Mean Z-score Gene Expression
B
Alveolar Type 1 cells
Alveolar Type 2 cells
B cells
Ciliated cells
Mast cells
Endothelial cells
Eosinophils
Fibroblasts
Neutrophils
Plasma cells
Macrophages
T,NK cells
cDC 1
cDC 2
pDCs
IgG
Naïve
antiCD4
Fraction of Cell Type
12518_Array2 12518_Array3 12518_Array4 12518_Array5 14018_Array2 14018_Array3 14018_Array4 14018_Array5
13818_Array8 13818_Array9 13818_Array10 13818_Array11 13818_Array12 13918_Array8 13918_Array9 13918_Array10 13918_Array11 13918_Array12
12818_Array2 12818_Array3 12818_Array4 12818_Array5 12818_Array6 13518_Array2 13518_Array3 13518_Array4 13518_Array5Gran 13518_Array6 14218_Array8 14218_Array9Granulo 14218_Array10 14218_Array11 14218_Array12
C
CD8A CD8B CD4 GZMK GPR183 CXCR4 CCL5 TOX EOMES IL10 IL10RA IL10RB GZMA GZMB PRF1 ITM2C CTLA4 LAG3 TOX2 HAVCR2 CD38 ENTPD1 NFKBIA TNF IFNG IL17A IL17F IL2RA IL23R BHLHE40 HIF1A ITGA1 ITGA6 ITGAE ZNF683 PRDM1 CXCR3 CXCR6 RORA RORC ZBTB16 CCR6 KLRG1 CX3CR1 TBX21 KLF3 GZMH AHNAK S1PR5 GNLY TYROBP KLRD1 NR4A1 EGR1 CD69 TIGIT BATF FOXP3 DUSP4 FURIN SPOCK2 RBPJ IL7R CAPG SAMSN1 TCF7 PECAM1 SELL LEF1 S1PR1
GZMK hi TEM/PEX-like
TEff-like
T1T17-like
Tc17-like
TEMRA-like
NK-like
ILC-like
Tregs
FURIN hi TM-like
TM-like
TNaive/CM-like
Mean Z-score Gene Expression
D
IgG vs Naïve
E
IgG vs αCD4
CD4 T cells
CD8 T cells
Innate-like T cells
Cytotoxic
Transcription factors
Cytokines, Chemokines
Co-activation, Co-inhibitory, Dysfunction, Exhaustion
IFN-response
Other
log2(CPM+1)
Naïve
IgG
αCD4
Up in IgG
Down in IgG
loge FC
log2|FC|>log2(1.3)
0.05>FDR, log2|FC|>log2(1.3)
F
Granuloma: #DE genes
0.05>FDR, log |FC|>log (1.3)
TEMRA-like
TEff-like
TM-like
TNaive/CM-like
FURIN hi TM-like
GZMK hi TEM/PEX-like
ILC-like
NK-like
T1T17-like
Tc17-like
Tregs
Naïve
IgG
αCD4
G
Pathway Activities Across Cell Types (IgG vs Naïve)
Androgen EGFR Estrogen Hypoxia JAK-STAT MAPK NFkB PI3K TGFb TNFa Trail VEGF WNT p53
H
Pathway Activities Across Cell Types (IgG vs antiCD4)

Figure S3. Coarse-grain cell types and T, NK cell diversity in the TB granuloma, related to Figures 3, 4. (A) Heatmap depicting gene expression profiles (mean z-score) of coarse cell-type markers. Marker genes are depicted on the x-axis, and coarse-cell types on the y-axis. **(B)** Coarse-grain cell type frequencies in the TB granuloma (naïve (left), IgG (middle), αCD4 (right)). Individual bars represent a single TB granuloma profiled using Seq-Well S^3. **(C)** Heatmap depicting gene expression profiles (mean z-score) of T,NK subpopulation markers, as well as select transcription factors, cytokine and chemokine receptors, co-inhibitory and co-activation markers. (**D**) T, NK cell pseudobulk Log_2(CPM+1) for naïve (light blue), IgG (yellow), and αCD4 (red) NHP granulomas (*** p<0.001,** p<0.01, *p<0.05; Wilcoxon rank-sum test). Heatmap depicting log_eFC of lineage markers, cytolytic molecules, select transcription factors, immunoregulatory molecules, and chemokines, and cytokines (rows) for each cell type (columns). White circles indicate log_e|FC|> log_e(1.3), relative to naïve or αCD4 granulomas. Black rectangles indicate 0.05>FDR and log_e|FC|> log_e(1.3), relative to naïve or αCD4 granulomas. **(F)** Number of differentially expressed genes among T, NK cell subpopulations with 0.05>FDR and log_e|FC|> log_e(1.3), naïve (light blue), IgG (yellow), and αCD4 (red). (**G**) Heatmap depicting PROGENy (derived from pseudobulk matrices) enrichment scores. Red indicates a pathway is enriched in IgG, whereas blue indicates it is downregulated (i.e., upregulated in naïve). (**H**) Heatmap same as (G) but depicting IgG (red) vs αCD4 (blue).

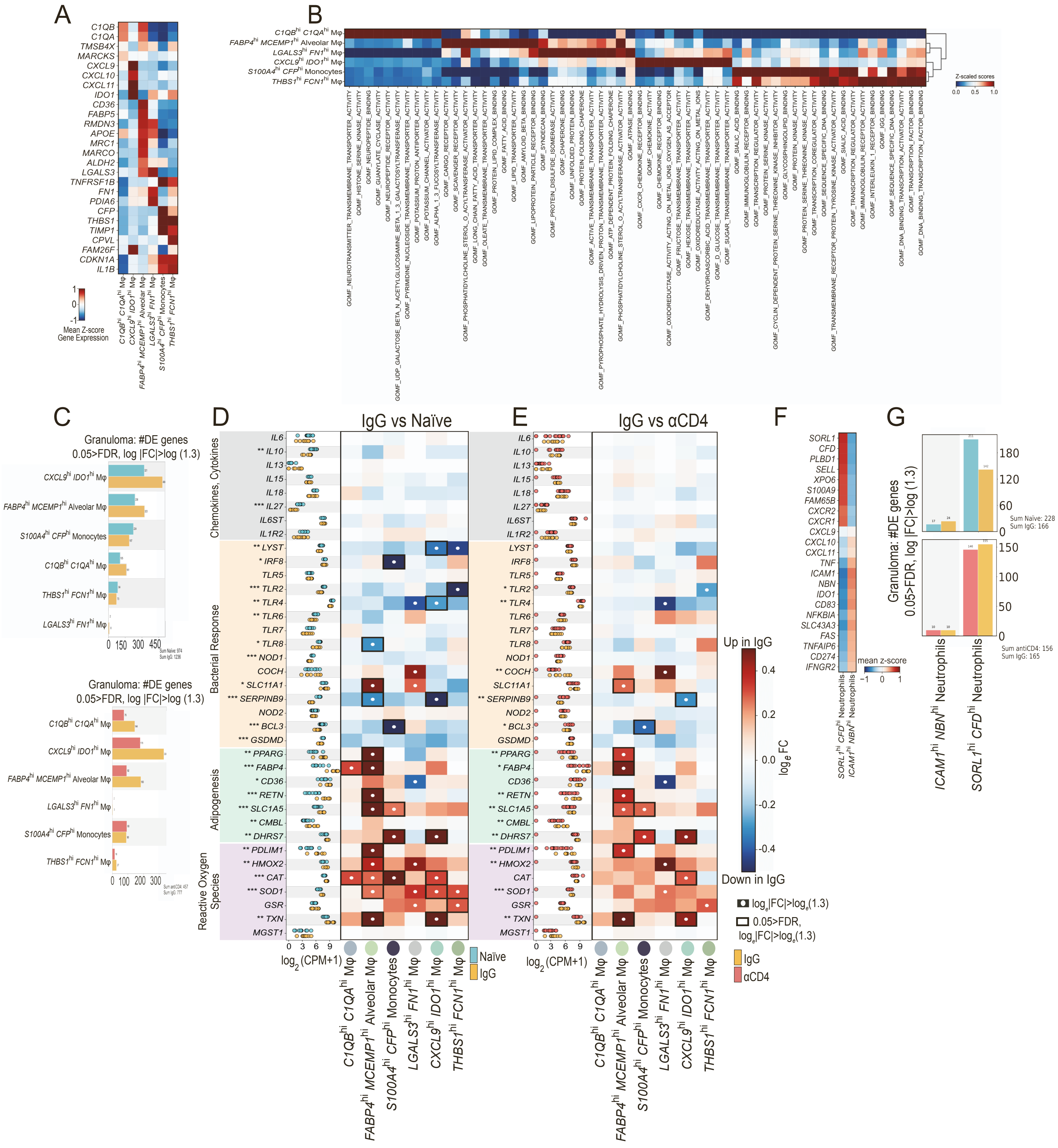

A
C1QB
C1QA
TMSB4X
MARCKS
CXCL9
CXCL10
CXCL11
IDO1
CD36
FABP5
RMDN3
APOE
MRC1
MARCO
ALDH2
LGALS3
TNFRSF1B
FN1
PDIA6
CFP
THBS1
TIMP1
CPVL
FAM26F
CDKN1A
IL1B
Mean Z-score Gene Expression
B
Z-scaled scores
C
Granuloma: #DE genes 0.05>FDR, log |FC|>log (1.3)
D
IgG vs Naïve
Chemokines, Cytokines
Bacterial Response
Adipogenesis
Reactive Oxygen Species
log2 (CPM+1)
Naïve
IgG
E
IgG vs αCD4
Up in IgG
Down in IgG
log_e FC
log_e|FC|>log_e(1.3)
0.05>FDR, log_e|FC|>log_e(1.3)
IgG
αCD4
F
SORL1
CFD
PLBD1
SELL
XPO6
S100A9
FAM65B
CXCR2
CXCR1
CXCL9
CXCL10
CXCL11
TNF
ICAM1
NBN
IDO1
CD83
NFKBIA
SLC43A3
FAS
TNFAIP6
CD274
IFNGR2
mean z-score
G
Granuloma: #DE genes 0.05>FDR, log |FC|>log (1.3)
Sum Naïve: 228
Sum IgG: 166
Sum antiCD4: 156
Sum IgG: 165

Figure S4. Monocyte-derived gene programming and neutrophil heterogeneity, related to Figures 5, 6. (A) Heatmap depicting gene expression profiles (mean z-score) of monocyte-derived subpopulation markers. Genes depicted on y axis and monocyte-derived subsets on the x axis. **(B)** Enriched GO Molecular Function (2021) pathways among monocyte-derived subsets (one macrophages subset vs rest (i.e., all other macrophage populations); Z-scaled scores). Calculated using Decoupler. (**C)** Number of differentially expressed genes (calculated using MAST) among monocyte-derived subpopulations with 0.05>FDR and log_e|FC|> log_e(1.3), naïve (light blue), IgG (yellow), and αCD4 (red). **(C-D)** Monocyte-derived pseudobulk Log_2(CPM+1) for naïve (light blue), IgG (yellow), and αCD4 (red) NHP granulomas (*** p<0.001,** p<0.01, *p<0.05; Wilcoxon rank-sum test). Heatmap depicting log_eFC of select chemokines and cytokines, bacterial response genes[S1], adipogenesis, and reactive oxygen species (rows) for each cell type (columns) in NHP granulomas, IgG vs naïve (C) or IgG vs αCD4 lesions (D). White circles indicate log_e|FC|> log_e(1.3), relative to naïve or αCD4 granulomas. Black rectangles indicate 0.05>FDR and log_e|FC|> log_e(1.3), relative to naïve or αCD4 granulomas. **(E)** Heatmap depicting neutrophil subpopulation marker gene expression profiles (mean z-score). Genes depicted on y axis and monocyte-derived subsets on the x axis. **(F)** Number of differentially expressed genes among neutrophil subpopulations with 0.05>FDR and log_e|FC|> log_e(1.3), naïve (light blue), IgG (yellow), and αCD4 (red).

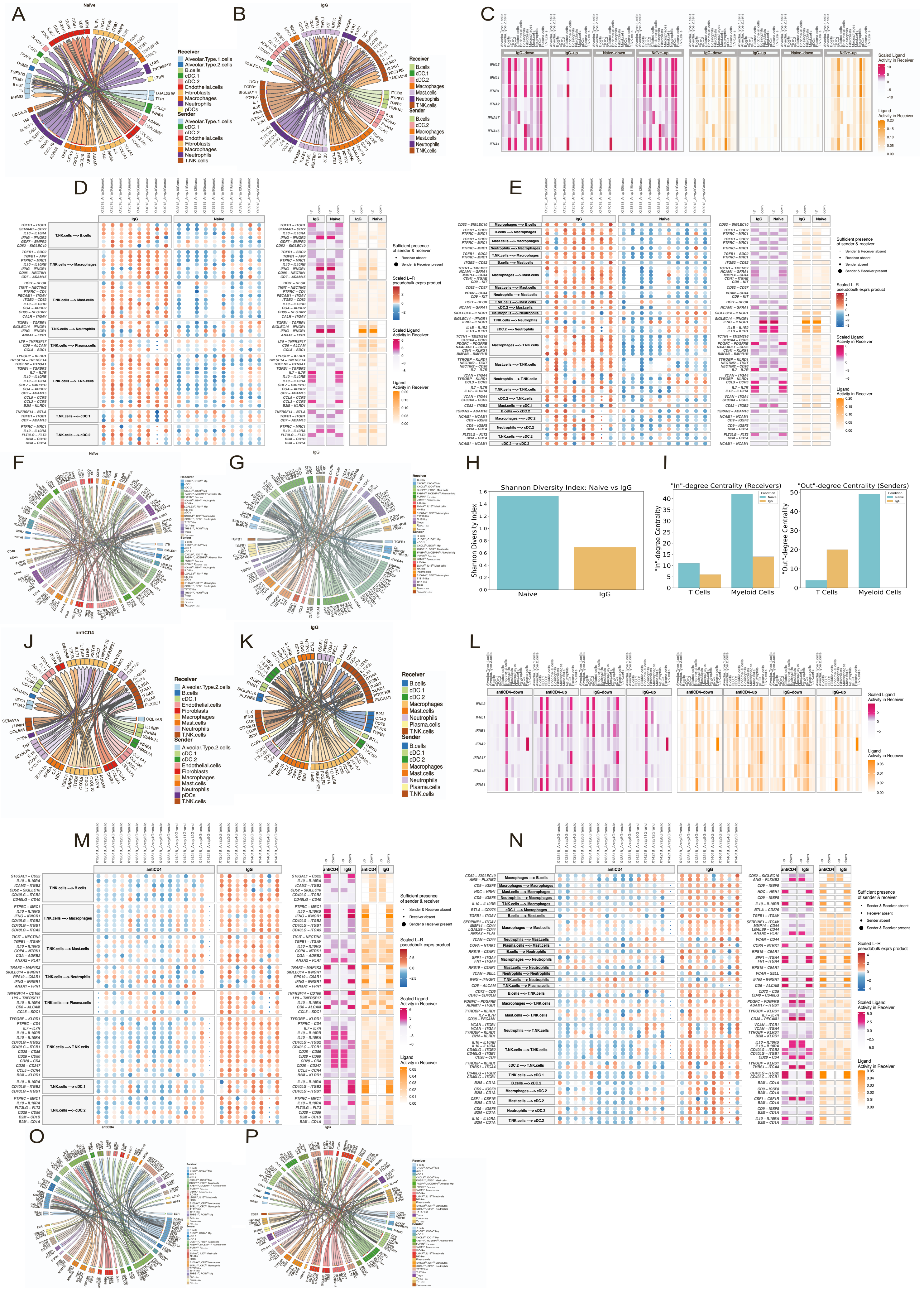
A
Naive
B
IgG
C
D
E
F
Naive
G
IgG
H
Shannon Diversity Index: Naive vs IgG
Shannon Diversity Index
Naive
IgG
I
"In"-degree Centrality (Receivers)
"In"-degree Centrality
"Out"-degree Centrality (Senders)
"Out"-degree Centrality
T Cells
Myeloid Cells
J
antiCD4
K
IgG
L
M
antiCD4
IgG
N
antiCD4
IgG
O
antiCD4
P
IgG

Figure S5. Cell-cell and ligand-receptor interactions during *Mtb* reinfection, related to Figure 7. (A) Circos plot depicting differential network interactions among coarse-grain cell type annotations in naïve granulomas (naïve vs IgG). Ribbon arrows indicate directionality (sender to receiver) populations. The outer edge color among senders denotes the sender cell type, with the inner edge color representing the receiver cell type. Circos plot depicts the 50 top-prioritized linkages. **(B)** Circos plot depicting differential cell-cell interactions among IgG lesions – same as in (A) but comparing IgG to naïve. **(C)** Heatmap depicting type 1 interferon ligand-activity among coarse cell types depicting both scaled ligand activity and ligand activity in receiver cell types **(D)** Heatmap (left) depiction of scaled ligand-receptor expression among the top 50 prioritized linkages among T, NK cells – as putative "sender" populations – from IgG granulomas, relative to naïve. Dot size represents L-R presence or absence. Columns represent individual granulomas. Heatmap split by NHP group. Heatmaps (right) are representations of the scaled ligand activity and ligand activity among putative receiver populations. **(E)** Heatmap similar to (D) but depicting the top 50 prioritized linkages among all coarse-grain cell types (all cell types depicted as "sender" or "receiver," if among top 50 DE linkages). **(F)** Circos plot showing differential network interactions among immune cell subpopulations in naïve granulomas (naïve vs IgG). Top 100 prioritized linkages are plotted. **(G)** Circos plot depicting differential cell-cell interactions among all immune cell subpopulations IgG vs naïve. **(H)** Shannon Diversity Index among *IL10* "senders" – a larger index represents increased diversity. **(I)** Degree Centrality among *IL10* "senders" and "receivers" – elevated centrality indicates more "incoming" and "outgoing" communication events. Myeloid cell centrality is among all cellular subsets of myeloid lineage. (**J**) Differential cell-cell interactions among αCD4 lesions (αCD4 vs IgG). **(K)** Differential cell-cell interactions among IgG lesions (IgG vs αCD4). **(L)** Heatmap depicting type 1 interferon ligand-activity among coarse cell types, IgG vs αCD4. **(M)** Heatmap similar to (D) but displaying the top 50 prioritized linkages among T, NK cells (only T, NK cells are depicted as "sender" populations) from IgG granulomas, relative to αCD4. **(N)** Heatmap similar to (D) but depicting the top 50 prioritized linkages among all coarsely annotated cell types from IgG granulomas relative to αCD4. **(O)** Circos plot depicting differential network interactions among

immune cell subpopulations in αCD4 granulomas (αCD4vs IgG). Top 100 prioritized linkages are plotted. **(P)** Differential cell-cell interactions among all immune cell subpopulations IgG vs αCD4.

Supplemental references

[S1]. Yao, Y., Jeyanathan, M., Haddadi, S., Barra, N.G., Vaseghi-Shanjani, M., Damjanovic, D., Lai, R., Afkhami, S., Chen, Y., Dvorkin-Gheva, A., et al. (2018). Induction of Autonomous Memory Alveolar Macrophages Requires T Cell Help and Is Critical to Trained Immunity. Cell *175*, 1634–1650.e17. https://doi.org/10.1016/j.cell.2018.09.042.
